# Supplementary material for: Identification of Hydatigera Species in Wildcats (Felis silvestris) from Central Spain
Source: Animals (Basel). 2025 Nov 19;15(22):3340. doi: 10.3390/ani15223340 (PMC12649228; doi:10.3390/ani15223340)

Supplementary file S1. Alignment of complete mitochondrial sequences of species of *Hydatigera* downloaded from Genbank. Base positions are referred to the sequence ON055368 (*H. taeniaeformis*). Gene positions are marked in color according to the annotation in sequence ON055368. Primers used in this work are located in the alignment and their positions in a graphical view of the mitochondrial DNA are indicated at the end of the alignment. The mitochondrial DNA is circular and the initial position of some of the original sequences were different; to have all the sequences in the alignment starting in the same position, the 5′ end of some sequences were moved after the 3′, according to this table:

ON055368: no positions were moved

FJ597547: positions 1-2323 were moved at the 3′ of the sequence

JQ663994: no positions were moved

NC037071: positions 1-2526 were moved

PP104554: positions 1-7619 were moved

LC008533: positions 1-2507 were moved

NC061206: no positions were moved

MW808981: no positions were moved

NC021141: no positions were moved

NC021142: positions 1-2470 were moved

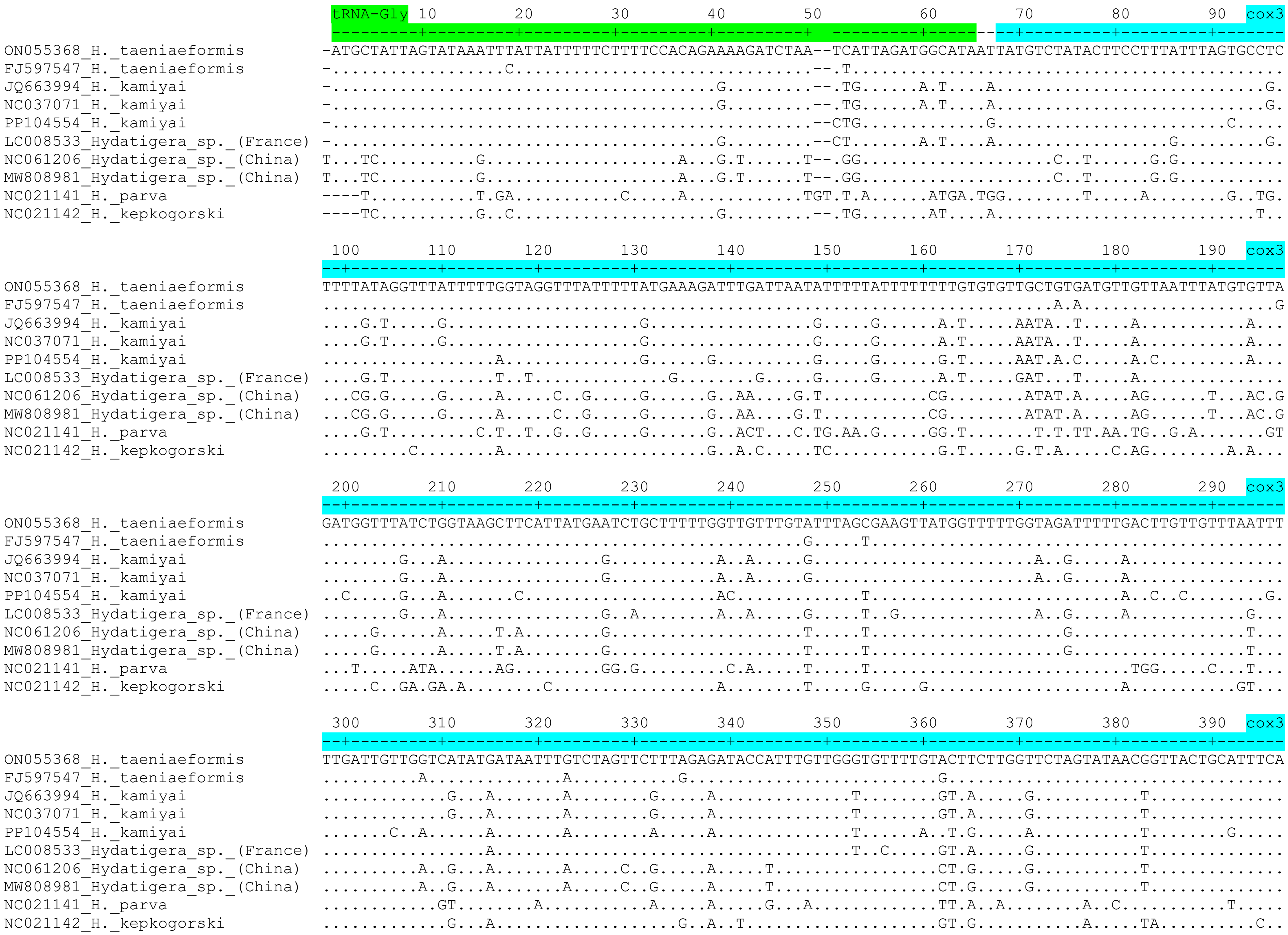
tRNA-Gly
cox3
ON055368_H._taeniaeformis
FJ597547_H._taeniaeformis
JQ663994_H._kamiyai
NC037071_H._kamiyai
PP104554_H._kamiyai
LC008533_Hydatigera_sp._(France)
NC061206_Hydatigera_sp._(China)
MW808981_Hydatigera_sp._(China)
NC021141_H._parva
NC021142_H._kepkogorski

```
                                   400       410       420       430       440       450       460       470       480       490   cox3
                                   --+---------+---------+---------+---------+---------+---------+---------+---------+---------+-------
ON055368_H._taeniaeformis          TCATTTGTTAGGTTGACGATATTGTGATTTTTTTTTACTTTTAACTATAGTGTTAGGGTTGGGGTTTGTTGTTTTACAAGTTGCTGAGATAGAGGATATA
FJ597547_H._taeniaeformis          ...............G................................T.........................................C.........
JQ663994_H._kamiyai                .......A.G.......A..................G..............T........A..T.....AA................A..T.........
NC037071_H._kamiyai                .......A.G.......A..................G..............T........A..T.....AA................A..T.........
PP104554_H._kamiyai                .................AG.......................G........T...........T.....A.....G...........A..T..A......
LC008533_Hydatigera_sp._(France)   ......A..........AG..............C........G........T.....A.....C.....AA.A..............A..T..A......
NC061206_Hydatigera_sp._(China)    ......A.....C....AG..C............C.GT.G..G........T.....T.....A.....G....................G..A..C..C
MW808981_Hydatigera_sp._(China)    ......A.....C....AG..C............C.GT.G..G........T.....T.....A.....G....................G..A..C..C
NC021141_H._parva                  ......A.........TAT.................GT.G.....G...A.T..G...C....T.....GT..........A.....A..T........T
NC021142_H._kepkogorski            ......AC....A....A.................................TC....AA.C..T.....A........GA.A..C..A..G..A......

                                   500       510       520       530       540       550       560       570       580       590   cox3
                                   --+---------+---------+---------+---------+---------+---------+---------+---------+---------+-------
ON055368_H._taeniaeformis          GGATTTAATTTAGTTGATTCTAGATTTTACTCAAGAAGTTTTTGTACGGTTGGTTTACATTTTAGTCATGTTTTATTAGGAGTAATAGGTTTGATTACTA
FJ597547_H._taeniaeformis          ......................................C........A...................................G................
JQ663994_H._kamiyai                ..G........G..G........T.....T..T.....A........T......C......................................G.G....
NC037071_H._kamiyai                ..G........G..G........T.....T..T.....A........T......C......................................G.G....
PP104554_H._kamiyai                ..G...........A........T.....T..T.....A........T...................................G........AG......
LC008533_Hydatigera_sp._(France)   ...........G.................T..T..G..A........T...........................C.................G.A....
NC061206_Hydatigera_sp._(China)    ..G....GCC....G........T.....T..T.....G........T.....C...........C..............T..T.........G....C.
MW808981_Hydatigera_sp._(China)    ..G....GCC....G........T.....T..T.....G........T.....C...........C..............T..T.........G....C.
NC021141_H._parva                  ..G.................A..T..C..T..T.....C........T..A..............C.....G.....G..C..TG.T.....ATCGGTG.
NC021142_H._kepkogorski            T.T...........A........T.....T..T..T...........T..A..A....................G..G.....T.........G....C.

                                   600       610       620       630       640       650       660       670       680       690   cox3
                                   --+---------+---------+---------+---------+---------+---------+---------+---------+---------+-------
ON055368_H._taeniaeformis          TATTTTGTGTTGGTAGCGTTAGCTTTGGGGTTTATCGCTGTACTGTTTTAACTTGATATTGACATTTTGTAGATTATGTTTGATTAATAGTTTATACTAT
FJ597547_H._taeniaeformis          .............................................................G......................................
JQ663994_H._kamiyai                ................T....AT.....T........T.......................G.....C..G......A......................
NC037071_H._kamiyai                ................T....AT.....T........T.......................G.....C..G......A......................
PP104554_H._kamiyai                ......................A.....A........T.....A................................C.......................
LC008533_Hydatigera_sp._(France)   .......C........T....AG.....T..A.....T.......................G........G.............................
NC061206_Hydatigera_sp._(China)    ..C....C........T....AG..............T...........G...........G..C..............A.................G..
MW808981_Hydatigera_sp._(China)    ..C....C........T....AG..............T...........G...........G..C..............A.................G..
NC021141_H._parva                  ..A.GCTG..G.....TAG..AA........G.....T..............A.................G........A...C..T.......C..C..
NC021142_H._kepkogorski            ..........A.....G.....A.....A........T..............A.................T......A.....C.T...........A..

                                   700 cox3  710       720 tRNA- 730 His   740       750       760       770          780       790
                                   --+---------+-------------+---------+------ ---+---------+---------+---------+   ---------+------+---
ON055368_H._taeniaeformis          TGTATATGTTTGTTAATAGTTTACTAATAGGTTATGAAA-TAAACTAATAGATTGTGGTTCTGTTGTATACTTT---TAGTATTAGTAAATTTATGTTTA
FJ597547_H._taeniaeformis          .......................................-..................................---.......................
JQ663994_H._kamiyai                ...G..............A-...................-..................................---...................C...
NC037071_H._kamiyai                ...G..............A-...................-..................................---...................C...
PP104554_H._kamiyai                ...............G.GA-...................-..................................---.......................
LC008533_Hydatigera_sp._(France)   .........A......GG.-...................-..................................---...................C...
NC061206_Hydatigera_sp._(China)    ...T.............-.-...............AT.T-......G..........................GA--.G..........C..G...A...
MW808981_Hydatigera_sp._(China)    ...T.............-.-...............AT.T-......G..........................GA--.G..........C..G...A...
NC021141_H._parva                  ...T............G---....CT...T.....-----......T....T......CG..A.A...C...G.GTT.G..G.GG......-G...A...
NC021142_H._kepkogorski            ...G...............G...............A...A......................A..........A----G............AG.......
```

```
                                 cytb 800       810       820       830       840       850       860       870       880       890
                                 ------+---------+---------+---------+---------+---------+---------+---------+---------+- --------+--
ON055368_H._taeniaeformis        ATTTATTGCGTTGTAATTTGATGGATTTGCCTATTAGTTTTTCTATAAATTATTATTGAAGTTATGGTTTTGTTCTTTCTATGTTTAT-GTGTGTTCAAA
FJ597547_H._taeniaeformis        ............................A...........C...............................................-....A......
JQ663994_H._kamiyai              .......A...C................A..A...........AG.T....................A.....A..G..A........-....A......
NC037071_H._kamiyai              .......A...C................A..A...........AG.T....................A.....A..G..A........-....A......
PP104554_H._kamiyai              G......A...C.......A..A........A.........................................G..C..AG.......-....A.....G
LC008533_Hydatigera_sp._(France) G......A...C.......A..A.....A..G...........A..T..........................G.....A........-....A......
NC061206_Hydatigera_sp._(China)  G...G..A...C.A........T.....A.....C..C......G..........C...........A....................-....A.....G
MW808981_Hydatigera_sp._(China)  G...G..A...C.A........T.....A.....C..C......G..........C...........A....................-....A.....G
NC021141_H._parva                G.G.G..A..AAAG.....AG.T.....A..A..A....A....T.G.......T...G..AA....G.....GT.A..A......G.T....-......
NC021142_H._kepkogorski          .......T....TG.....A..A.....A..A.......A................................................-....A.C....

                                 cytb 900       910       920       930       940       950       960       970       980       990
                                 -------+---------+---------+---------+---------+---------+---------+---------+---------+---------+--
ON055368_H._taeniaeformis        TTATTACTGGTGTAATATTATCTTTTTTGTACGTTAGTAATTTTGATGTTAGATTTTCAATTGTTACTAGGTTTTCCAAAGATTCTTTTTTTACTTGATG
FJ597547_H._taeniaeformis        ......................C.....T...C...........C..A......................A.....T.......................
JQ663994_H._kamiyai              ..............G.G..G...........T..CG..G...C..C......T......G.A........A.....T.......................
NC037071_H._kamiyai              ..............G.G..G...........T..CG..G...C..C......T......G.A........A.....T.......................
PP104554_H._kamiyai              ...........A..G.C..G........A..T......AG..C.AT......G.................A.............................
LC008533_Hydatigera_sp._(France) ..............G.G..............T......G...C.AC......T......G.A....T...A.....T........C..............
NC061206_Hydatigera_sp._(China)  ..........C..GG.GC....A..CC..........G....C.A.C..........TTG.G........T..G..T.................A.....
MW808981_Hydatigera_sp._(China)  ..........C..GG.GC....A..CC..........G....C.A.C..........TTG.G........T..G..T.................A.....
NC021141_H._parva                .A...T....GA.TT.T..............TA.C..G....C..TAA....CT...TT.C.........A.....A......GAA..............
NC021142_H._kepkogorski          ....A........TG.......A........TA....GG...CAGC......G....TT.A.........AC....T..T....................

                                 cytb 1000      1010      1020      1030      1040      1050      1060      1070      1080      1090
                                 -------+---------+---------+---------+---------+---------+---------+---------+---------+---------+--
ON055368_H._taeniaeformis        CTTACGATACTTTCATATAATCGGGGTTAATATTTTGTTTATCTTAGTTTTTATTCACATGGGTCGTTCTTTATATTATTCTAGATATAAAAAGATTAGT
FJ597547_H._taeniaeformis        .....................T...........A....................C.............................................
JQ663994_H._kamiyai              T..G.....T..............T........A........TC.T..A.....A..T..........................G....G....G.....
NC037071_H._kamiyai              T..G.....T..............T........A........TC.T..A.....A..T..........................G....G....G.....
PP104554_H._kamiyai              T.....G..T...........T..T........A..A.....T..GA........A..T...........A..G................GT...G.....
LC008533_Hydatigera_sp._(France) T.....G..T...........T..T......G.A........TC.TA.A.....A..T..............G...........T....GG.........
NC061206_Hydatigera_sp._(China)  T..G..T..T..A.....G..G..A..G...G.GC.A.....TC.GA.A...G....TT.AT......................T....GT...G....G
MW808981_Hydatigera_sp._(China)  T..G..T..T..A.....G..G..A..G...G.GC.A.....TC.GA.A...G....TT.AT......................T....GT...G....G
NC021141_H._parva                T..........GG.....GG.T.....A..A..A..A.....A...T.G...G....T...T.C...............GGG..T....G...AG..GCG
NC021142_H._kepkogorski          T.....T..T..A.....T..T.........G.A.....C..TC.T...........T..TT..........G...........T..C.CC...G.AG..

                                 cytb 1100      1110      1120      1130      1140      1150      1160      1170      1180      1190
                                 -------+---------+---------+---------+---------+---------+---------+---------+---------+---------+--
ON055368_H._taeniaeformis        GTTTGGAAAGTTGGATTTCTTTTATATTTATTAATAATGGGTGAAGCTTTTACCGGGTATATTTTGCCATGGCATCAAATGTCTTACTGAGCAGCTACTG
FJ597547_H._taeniaeformis        .............................G..........................A..............A.....G........T.............
JQ663994_H._kamiyai              .....A............A.......C..G...G...................T...........A..G.................T.....G.......
NC037071_H._kamiyai              .....A............A.......C..G...G...................T...........A..G.................T.....G.......
PP104554_H._kamiyai              ..................G..............G.............G.....T...........A.....A..............T.....G.......
LC008533_Hydatigera_sp._(France) ..A..A............A..............G.G........G........T..............G..A..............T.............
NC061206_Hydatigera_sp._(China)  ..A..A..T.....T...A..A.G.....G...G.T.....C..G...........C........A..G..A..............T.....G.......
MW808981_Hydatigera_sp._(China)  ..A..A..T.....T...A..A.G.....G...G.T.....C..G...........C........A..G..A..............T.....G.......
NC021141_H._parva                .....A..T..G......G.A.....C...A....G...AT............A..T.....A..A.................A.....G..G..A....
NC021142_H._kepkogorski          .........A........A.C.....C.....G..G.................T........A.....T..............A..T.............
```

```
                                  cytb 1200      1210      1220      1230      1240      1250      1260      1270      1280      1290
                                  -------+---------+---------+---------+---------+---------+---------+---------+---------+---------+--
ON055368_H._taeniaeformis         TGTTGACTTCGATAGTTGATAGTTTGCCAGTTGTGGGTAGGATTATTTATAAGTATGTTGTAGGTGGTTTTTCTATTACTGGCGATACATTAATGCGTGT
FJ597547_H._taeniaeformis         ........................................T..............C.....G....................T.................
JQ663994_H._kamiyai               .A..A..C..T.......................T.....TG...................T..A..A..............T.................
NC037071_H._kamiyai               .A..A..C..T.......................T.....TG...................T..A..A..............T.................
PP104554_H._kamiyai               ....A..A..T..............A...A....T.....T....................G..A..A..............T.................
LC008533_Hydatigera_sp._(France)  ..........T..................A....T.....TG...................T..A..G..............T.................
NC061206_Hydatigera_sp._(China)   .T........T.......G...CC....TA....T.....TG........G.......G..T.....A.....A..A.....T.....T..G......A.
MW808981_Hydatigera_sp._(China)   .T........T.......G...CC....TA....T.....TG........G.......G..T.....A.....A..A.....T.....T..G......A.
NC021141_H._parva                 .T.....A..A..TA..AG...A..A....GGT.T.....T..AG.....G..........T..C..G.........T....T..A..TC.GGCT..G..
NC021142_H._kepkogorski           ....A.................G..A........A...CAC.GC.......................G...........A..T..C..G..G.GA.....
                                                                                                  HD (FORWARD)
                                  cytb 1300      1310      1320      1330      1340      1350      1360      1370      1380      1390
                                  -------+---------+---------+---------+---------+---------+---------+---------+---------+---------+--
ON055368_H._taeniaeformis         ATTATCTGTACATGTGTGTTTAGGTTTTATTATAATAATGTTAATGGTCATTCATTTGTTTTATCTTCACAAGGATGGTAGAAGTAATCCTTTATATAAT
FJ597547_H._taeniaeformis         .....................................................................T....................C.........
JQ663994_H._kamiyai               G.....C........A........A.....................A.TG.......A...........T..T.G...G..T..............C.G.
NC037071_H._kamiyai               G.....C........A........A.....................A.TG.......A...........T..T.G...G..T..............C.G.
PP104554_H._kamiyai               ......C..G...........G..A.........G...........A.T........A.....C.....T..AA....G..T.....C............
LC008533_Hydatigera_sp._(France)  ...............A........A.......................TG.......A...........T..T.G...A....................A
NC061206_Hydatigera_sp._(China)   .C.......T..CA.A..C..G..G...G.AG.C..C.....T.....TG.......A...........T.....G...........C.........TG.
MW808981_Hydatigera_sp._(China)   .C.......T..CA.A..C..G..G...G.AG.C..C.....T.....TG.......A...........T.....G...........C.........TG.
NC021141_H._parva                 T..T...A.T.....T...C.G......T.G..GT.GGGAG.G..TA.AG.A...A..CAA...T.A..TTCTAG......T..A............TT.
NC021142_H._kepkogorski           G......A.T...A.A.....G..G.....AG.T..............TG.......A......T.A..T..............A...T.........G.

                                  cytb 1400      1410      1420      1430      1440      1450      1460      1470      1480      1490
                                  -------+---------+---------+---------+---------+---------+---------+---------+---------+---------+--
ON055368_H._taeniaeformis         TATAAAGGTTTATCTGATGTAGTTTATTTTCACTCATATTTTACAGTGAAGGATTTATTTTTATTTGTGAGTATTCTAACTGGAGTATTAAGTTGATTGC
FJ597547_H._taeniaeformis         ................................T.............C.....................................................
JQ663994_H._kamiyai               .T......G..G........GA..........T..............A.....................T..........A.....G.....C.....AT
NC037071_H._kamiyai               .T......G..G........GA..........T..............A.....................T..........A.....G.....C......T
PP104554_H._kamiyai               ........A............A..........T..............A.....................T..........A..................T
LC008533_Hydatigera_sp._(France)  .T....A.G.....C...A.............T...........GA.A...........A.........T..........A.....G............T
NC061206_Hydatigera_sp._(China)   .......T...G........TA....C.....T.....C.....TA.............G......A..T.C........A........T......C..T
MW808981_Hydatigera_sp._(China)   .......T...G........TA....C.....T.....C.....TA.............G......A..T.C........A........T......C..T
NC021141_H._parva                 .T...CATGCAT......A..A.A........T.......................G...C.T..AA...T.........A.....GC...........T
NC021142_H._kepkogorski           ....GGATG.....A.....T........C..T..T........T..A..................A..........T..A.....G............T

                                  cytb 1500      1510      1520      1530      1540      1550      1560      1570      1580      1590
                                  -------+---------+---------+---------+---------+---------+---------+---------+---------+---------+--
ON055368_H._taeniaeformis         TATTAAGTCCAGATTTATTAGTAGATATAGAATCTTATCTTGAAGCTGATCAACTTAATACTCCAGTTAGAATAAAGCCAGAATGATACTTTTTAGCTTT
FJ597547_H._taeniaeformis         ................G.....T..............C..............................................................
JQ663994_H._kamiyai               ....G.C...T..C........T...........A.................G.....C.....T..............T.....G..............
NC037071_H._kamiyai               ....G.C...T..C........T...........A.................G.....C.....T..............T.....G..............
PP104554_H._kamiyai               .G..G.C...T......C....T........G...........G....................T..............T........T........A..
LC008533_Hydatigera_sp._(France)  ....G.C...T...........T...........A.......................C.....T..............T........T...........
NC061206_Hydatigera_sp._(China)   ..........T.....T.................G........G...........A........T...........C..T.....G..............
MW808981_Hydatigera_sp._(China)   ..........T.....T.................G........G...........A........T...........C..T.....G..............
NC021141_H._parva                 .T...T....T......A.G..T........G.....CA....G.............GC.....T..............T........T......AG...
NC021142_H._kepkogorski           .G........T.....GC.G..T..C...........C..A..G..............C.....T..A...........T........T.....G.....
```

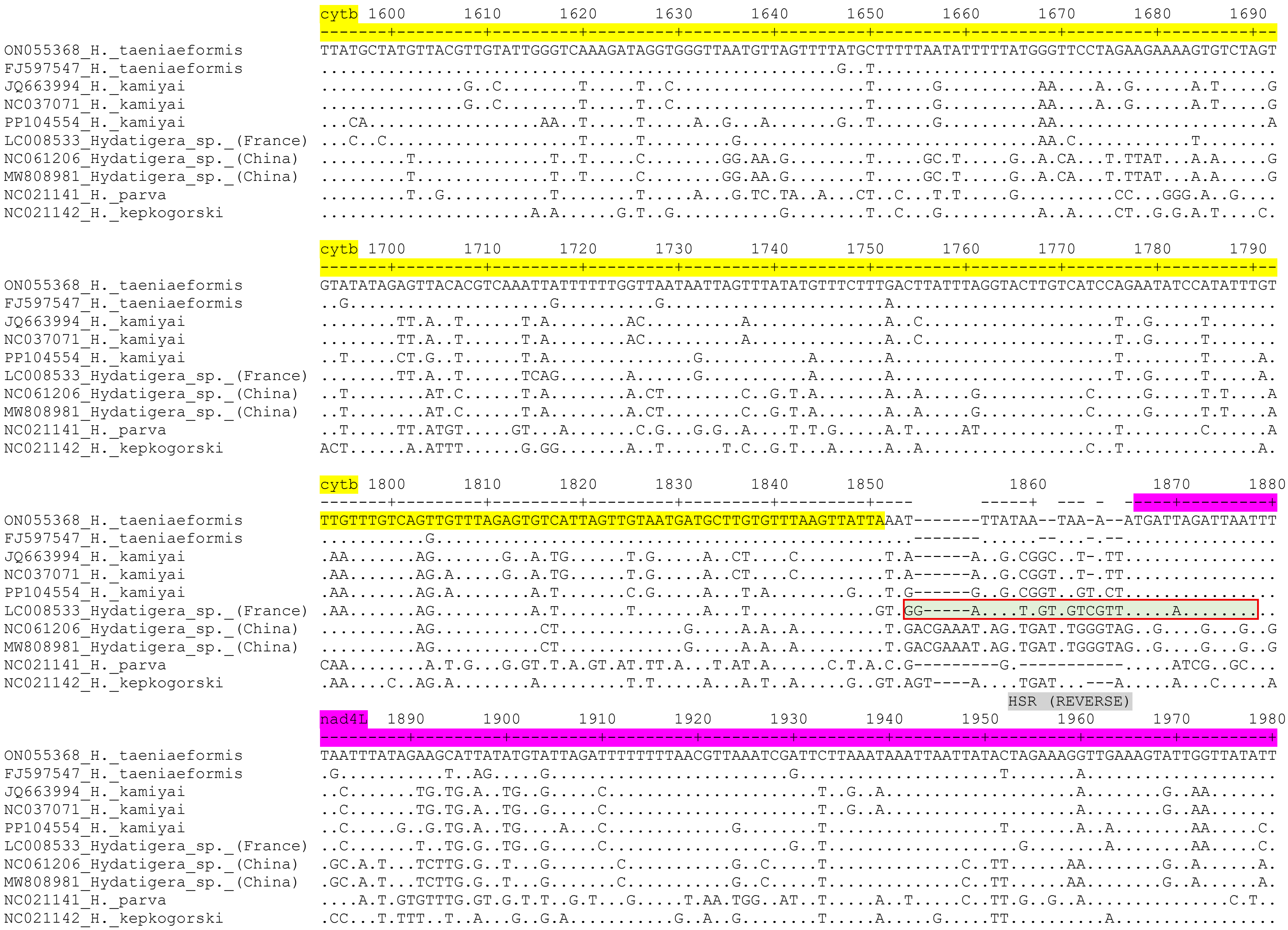
cytb
nad4L
HSR (REVERSE)
ON055368_H._taeniaeformis
FJ597547_H._taeniaeformis
JQ663994_H._kamiyai
NC037071_H._kamiyai
PP104554_H._kamiyai
LC008533_Hydatigera_sp._(France)
NC061206_Hydatigera_sp._(China)
MW808981_Hydatigera_sp._(China)
NC021141_H._parva
NC021142_H._kepkogorski

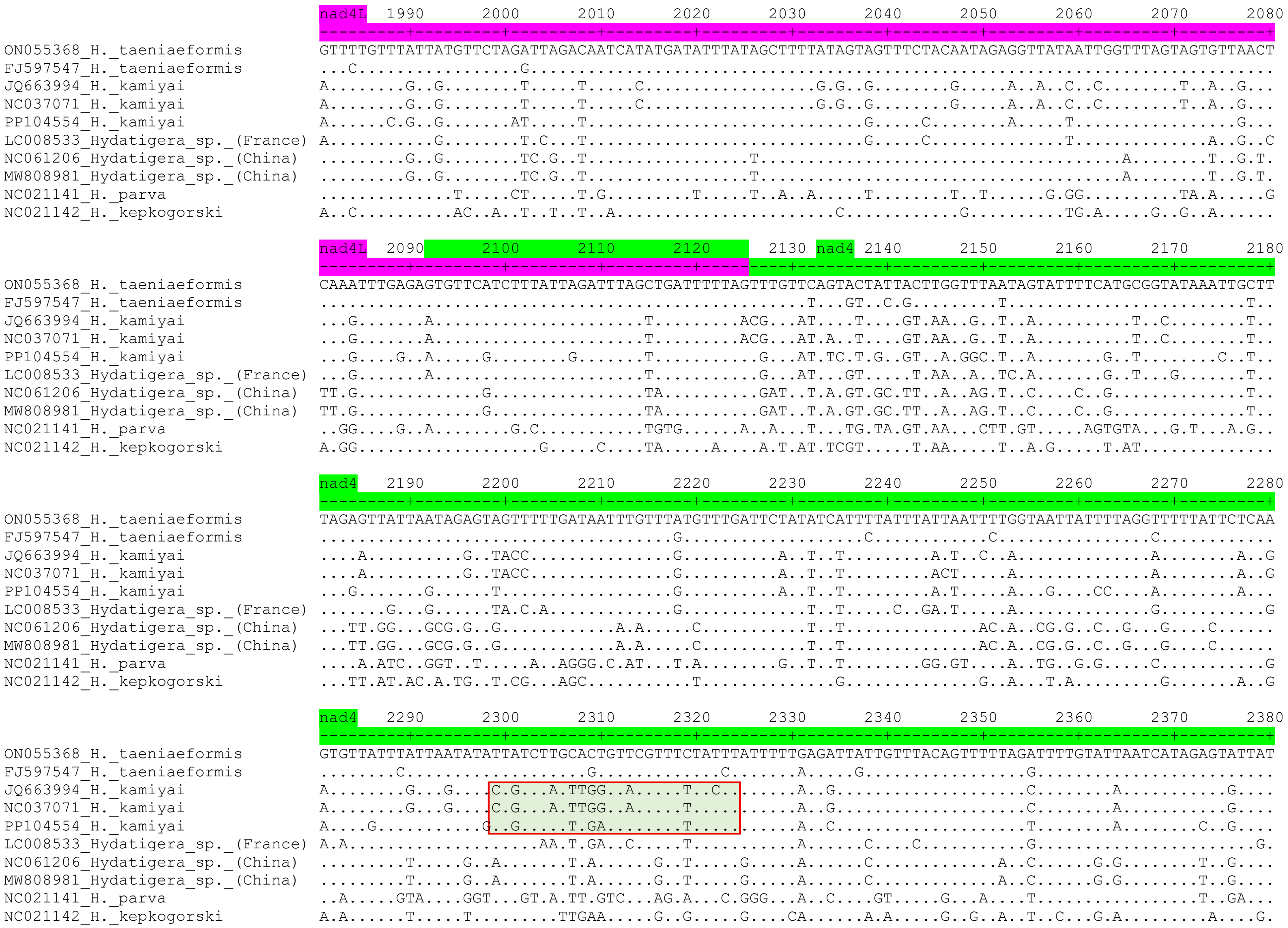
nad4L
1990 2000 2010 2020 2030 2040 2050 2060 2070 2080
nad4L
2090 2100 2110 2120 2130
nad4
2140 2150 2160 2170 2180
nad4
2190 2200 2210 2220 2230 2240 2250 2260 2270 2280
nad4
2290 2300 2310 2320 2330 2340 2350 2360 2370 2380
ON055368_H._taeniaeformis
FJ597547_H._taeniaeformis
JQ663994_H._kamiyai
NC037071_H._kamiyai
PP104554_H._kamiyai
LC008533_Hydatigera_sp._(France)
NC061206_Hydatigera_sp._(China)
MW808981_Hydatigera_sp._(China)
NC021141_H._parva
NC021142_H._kepkogorski

```
                                nad4      2390      2400      2410      2420      2430      2440      2450      2460      2470      2480
                                ---------+---------+---------+---------+---------+---------+---------+---------+---------+---------+
ON055368_H._taeniaeformis       TTTGAATAAGATATGAATTATCAATGCTTCCATTATTATTTTAATATTTAGTGAGTCTCCTTATTCAGAACGTTTTTTAGCAGGGTGGTATTTTAGTGG
FJ597547_H._taeniaeformis       ................................C.G.................................................................
JQ663994_H._kamiyai             ....G..............G..G........T..G..G.A...G..........................G...........C..A.........T....
NC037071_H._kamiyai             ....G..............G..G........T..G..G.A...G..........................G...........C..A.........T....
PP104554_H._kamiyai             .....G................G........T...A...A...G........G........A....................T..A..A......T....
LC008533_Hydatigera_sp._(France) ....GG....G..C..G..............T..G....AC...........................G.G...........T..A.........T....
NC061206_Hydatigera_sp._(China) ....GT....G..C..............C..........A.....................G..C.....G...........T..T..A..C.....C..
MW808981_Hydatigera_sp._(China) ....GT....G..C..............C..........A.....................G..C.....G...........T..T..A..C.....C..
NC021141_H._parva               .....T...CT.....G.....G...........GC.T.AC..........AG..T.....A.....T..G..G........T..T.........TTA..
NC021142_H._kepkogorski         ..........G..C..G.........T....T..G....A............C..A.....G..C..T..G...........T..T..A...........

                                nad4      2490      2500      2510      2520      2530      2540      2550      2560      2570      2580
                                ---------+---------+---------+---------+---------+---------+---------+---------+---------+---------+
ON055368_H._taeniaeformis       TTATCTTATAAGTACTAGTTTACCTTTGATTTTAGTATTGTTGTACTTATCATTTGTAAATAATTCATTTTTTTTTAGAGATTGAAGCATGTCTCATAAC
FJ597547_H._taeniaeformis       ...............A..........................A........C...A.....G......................................
JQ663994_H._kamiyai             .....................G.....A.................T.....T...T....AGGC..G..........TG.G....T.TGA..TCA..GGT
NC037071_H._kamiyai             .....................G.....A........G........T.....T...T....AGGC..G..........TG......T.TGA..T.A..GGT
PP104554_H._kamiyai             ..................A..G.....A........G..A.....T..G..T...T....AGG...T..........ATA....GTATG.A.T.G.C.GT
LC008533_Hydatigera_sp._(France) ....T.A............CG......A........GC.......T..G..T...T....AGGA.............TG.....GTATGC..T.G..GGT
NC061206_Hydatigera_sp._(China) ....T.G........A..C..G...C.T......A....A..A..T.....T..CT.G...G....G...........TA....GTAT.AT.G.G....G
MW808981_Hydatigera_sp._(China) ....T.G........A..C..G...C.T......A....A..A..T.....T..CT.G...G....G...........TA....GTAT.AT.G.G....G
NC021141_H._parva               ....T.G..GGT...G..A...........A.....T..A..A..T.......A......A.....T.....A............TTGT..GGAG..G.T
NC021142_H._kepkogorski         ....T.A.....................G.....A.T..A..A..T........CT....AG.......C........G......CAT..AGT.GG..GT

                                nad4      2590      2600      2610      2620      2630      2640      2650      2660      2670      2680
                                ---------+---------+---------+---------+---------+---------+---------+---------+---------+---------+
ON055368_H._taeniaeformis       TGCTTAAAAATTATTTATTATTTGATATCTTTTATATTTTTTACTAAGGTTCCTTTGCTTCCATTTCATACATGATTACCTGTAGTACATGCTGAAGCTA
FJ597547_H._taeniaeformis       ..T....................A....................C..............C.....................A..................
JQ663994_H._kamiyai             ..TCCT..G..A...........A.....A..............A...........AA...................G..AA....G...........C.
NC037071_H._kamiyai             ..TCCT..G..A...........A.....A..............A...........AA...................G..AA....G...........C.
PP104554_H._kamiyai             ..TCCT..G..A...........A....................A...........AG.......................A.......C........C.
LC008533_Hydatigera_sp._(France) ..TCCT..G..A.....C.....A.....A..............A...........AG......................AA.......C..........
NC061206_Hydatigera_sp._(China) CATGG..T.G..G.....GT...A...........T........C............A....T..............G..GA.T..T........G....
MW808981_Hydatigera_sp._(China) CATGG..T.G..G.....GT...A...........T........C............A....T..............G..GA.T..T........G....
NC021141_H._parva               A.A..G..GG..G.G.........T.G........T....................AT....G...........G..G...A....T...........AT
NC021142_H._kepkogorski         .CT.ATT.T..GG..........AG.G........T.................G...G..........C..G........AA..........A.......

                                nad4      2690      2700      2710      2720      2730      2740      2750      2760      2770      2780
                                ---------+---------+---------+---------+---------+---------+---------+---------+---------+---------+
ON055368_H._taeniaeformis       CTAGTATAGTATCAATTTTTTTGAGTGGATATATTATGAAGTTGGGTTTATTAGGAGTATATCGTTGTTCATATTTTATATTTGATCCTAATTTTATGTG
FJ597547_H._taeniaeformis       ..................................C..............................................................A..
JQ663994_H._kamiyai             .............T..C.....A....................A...C.......T..T...........................T..G.A...T.AG.
NC037071_H._kamiyai             .............T..C.....A....................A...C.......T..T...........................T..G.A...T.AG.
PP104554_H._kamiyai             ....A........T........A..A........C........A...........T..T...........................TT.G.G...T.AG.
LC008533_Hydatigera_sp._(France) .............T........A.....G..............A.....G........T..C........................TT.G.C...T..G.
NC061206_Hydatigera_sp._(China) ....G.....T..T........A..G....................CA..C....C..T..............C............TA...A..AT.AAA
MW808981_Hydatigera_sp._(China) ....G.....T..T........A..G....................CA..C....C..T..............C............TA...A..AT.AAA
NC021141_H._parva               ....A.....G..T..A.....A.....G.....A........A........G..T..G.........A.T.T....G.G......ATA........AAT
NC021142_H._kepkogorski         ....A.....G..C........A..A..G..............A..............T.............GA............TA.C.G..AT.AG.
```

```
                                   nad4      2790      2800      2810      2820      2830      2840      2850      2860      2870      2880
                                   ---------+---------+---------+---------+---------+---------+---------+---------+---------+---------+
ON055368_H._taeniaeformis          ATATCTTGCTTTTTGTTGTTTGGCTTGTATAGGCTTTTTAGTAACAGCATGTACAGAATTAGATGGAAAGCGGTGATTGGCATTTTTAAGTTTAGCTCAT
FJ597547_H._taeniaeformis          ...............C.................T............................................A.................C...
JQ663994_H._kamiyai                T.......T............A..C........T............T.......T..G................................A.........
NC037071_H._kamiyai                T.......T............A..C........T............T.......T...................................A.........
PP104554_H._kamiyai                T.....CAT............A..A........A..C.........T.......T..G..............A........G.....G..G..G......
LC008533_Hydatigera_sp._(France)   T....................A..C........T............T.......T..G..............A........G........A.........
NC061206_Hydatigera_sp._(China)    T...........G........A..C...G.G..G......A.T..T..G..C..............T.....A..G..A..............G..C...
MW808981_Hydatigera_sp._(China)    T...........G........A..C...G.G..G......A.T..T..G..C..............T.....A..G..A..............G..C...
NC021141_H._parva                  T...A..TT.G.......CG.T.TGA..G.TT.T.....TA.TGTGT.G....GT..G..............A........T........G...T.A...
NC021142_H._kepkogorski            C.......T...A.....C..AA.A........T......A..GT...G.....T...........G.....T..G..A...........A..G.....C

                                   nad4      2890      2900      2910      2920      2930      2940      2950      2960      2970      2980
                                   ---------+---------+---------+---------+---------+---------+---------+---------+---------+---------+
ON055368_H._taeniaeformis          ATTGTTGTTCCTTTTATTGGCTTTTATATAAGTGATTGAAGGTCAGTTAACTATATATTTTTTTACTGTTTGGGGCATGGTTTAAGTGCAGGTATTGTAT
FJ597547_H._taeniaeformis          ....................T..C.....C....................T..............................C..................
JQ663994_H._kamiyai                ..A.................T..........A........AC........T..............T.....A.................T.....C..T.
NC037071_H._kamiyai                ..A.................T...................AC..G.....T..............T.....A.................T.....C..T.
PP104554_H._kamiyai                ..A..A..C...........T..............C..G.AC........T.G....................................T..........
LC008533_Hydatigera_sp._(France)   ..A.................T...................AC........T...........C..T.....A..A..............T........T.
NC061206_Hydatigera_sp._(China)    ...A.CA....A...G....T..A......T.C........A..T....GT...T.G..G.....T.....A........A.................G.
MW808981_Hydatigera_sp._(China)    ...A.CA....A...G....T..A......T.C........A..T....GT...T.G..G.....T.....A........A.................G.
NC021141_H._parva                  ..A..C.....A...T.GT.T....T.G.TT..........T...A.A.G.....G.........T.....A..T.....G.....A..G........G.
NC021142_H._kepkogorski            .....A..A..A.....A..G...........A........T..TA.A..T...G.G........T.....A..T..C..G........G.....A..T.

                                   nad4      2990      3000      3010      3020      3030      3040      3050      3060      3070      3080
                                   ---------+---------+---------+---------+---------+---------+---------+---------+---------+---------+
ON055368_H._taeniaeformis          TTGGATTATTATGATTATTTTATGATCTATGTCATACTCGAAAATGAATTTTGTTGAAGTCTAGCGTAAATGGAATTGGTTATATGATAAGAGTCATTTT
FJ597547_H._taeniaeformis          ....G....................................................................G..........................
JQ663994_H._kamiyai                ....G..G..G..GC.G.........T..........C..T...........A...........TA.......TG..A........T.GT.T..GG.AA.
NC037071_H._kamiyai                ....G..G..G..GC.G.........T..........C..T...........A...........TA.......TG..A........T.GT.T..GG.AA.
PP104554_H._kamiyai                .......G..G.....G.........T.G...........T.....................G.TA.......TG..AA.......T..T.T..GG..A.
LC008533_Hydatigera_sp._(France)   ....T.....G..G............T.G........C..T..............A......G.TA.......T.C.A........C..T.T..AG..A.
NC061206_Hydatigera_sp._(China)    ....CA.G.....G..G.........T.....A.C..A..T..T..GG....A..A........AA.......TG..A...TA.....GCTT..G..AG.
MW808981_Hydatigera_sp._(China)    ....CA.G.....G..G.........T.....A.C..A..T..T..GG....A..A........AA.......TG..A...TA.....GCTT..G..AG.
NC021141_H._parva                  ..........G....GTA........A.T.C.A.C........T...G.A..............AA.T.GG.ATG..AAA.T....T...TT..A...G.
NC021142_H._kepkogorski            .............G..G.........T.....A.C........T........A..A......G.TA.......T.......T....T...TT..GG.GG.

                                   nad4      3090      3100      3110      3120      3130      3140      3150      3160      3170      3180
                                   ---------+---------+---------+---------+---------+---------+---------+---------+---------+---------+
ON055368_H._taeniaeformis          TAGGTTATTAAGATTATGTTCATTTCCTACAACTATTCAATTTTTTTCTGAAGTGAATTTGGTTATACAAAGGTCTGGTTTAATTATTTATATATTATTT
FJ597547_H._taeniaeformis          ............G...........................................C.......G...................................
JQ663994_H._kamiyai                ...................................................G..A..A........................G........G.G......
NC037071_H._kamiyai                ...................................................G..A..A........................G........G.G......
PP104554_H._kamiyai                ......G..G.......................C....................A..A........................G........G.G......
LC008533_Hydatigera_sp._(France)   ............G.........................................A.....A...........A.........G.G......GC.......
NC061206_Hydatigera_sp._(China)    ...AA.G..G..G........C........T........G...........G..T...........C..G........GC.............T......
MW808981_Hydatigera_sp._(China)    ...AA.G..G..G........C........T........G...........G..T...........C..G........GC.............T......
NC021141_H._parva                  G.....G........G.....T......C....GG....G.......GC..G..A.....AA..GGTTG.T.TGT.AA.A.TT.AT.A........G...
NC021142_H._kepkogorski            ...AA.G.....T........T........T....................G..A..G..A..A.C...G..TG..A........G.....T....G...
```

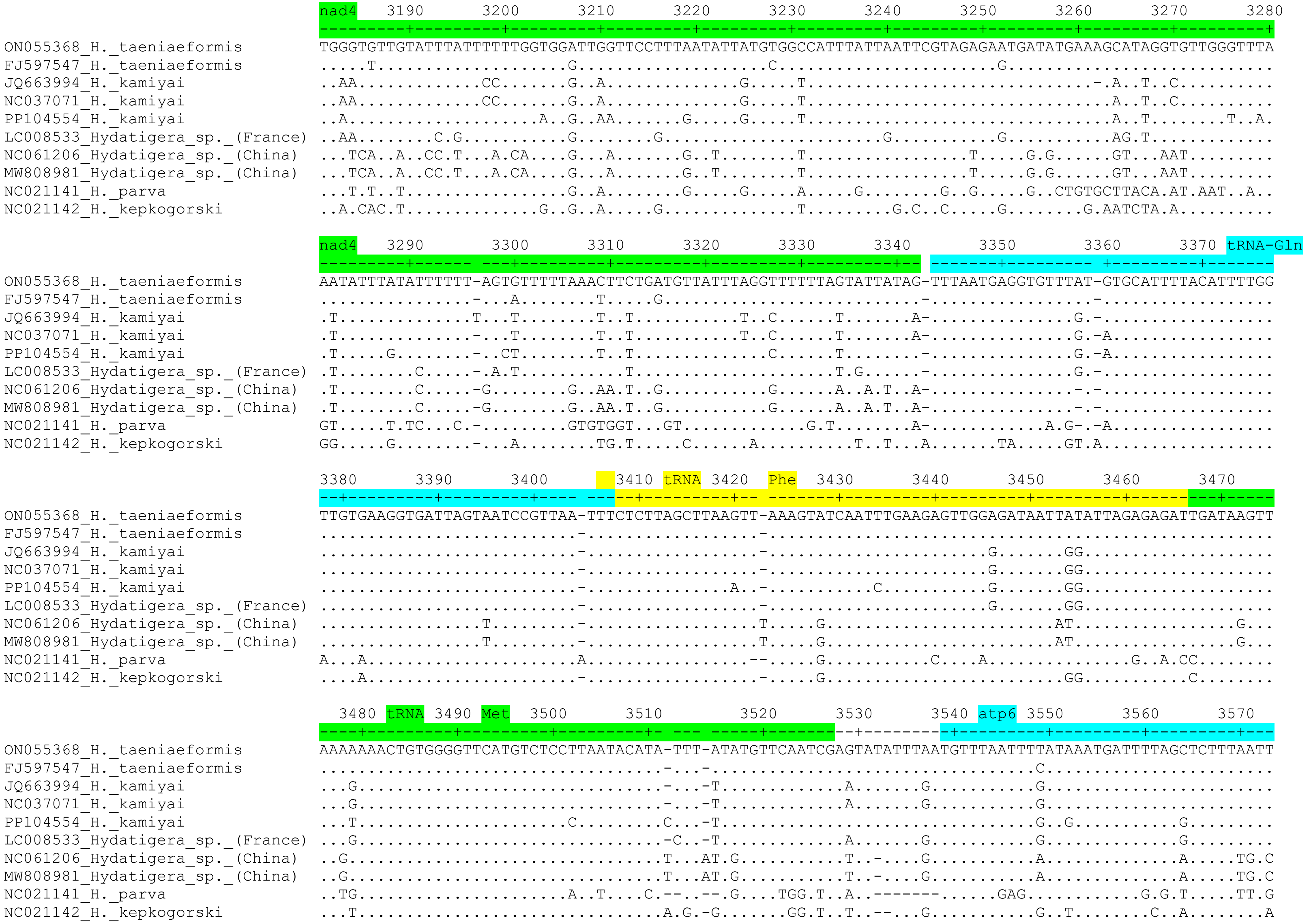

nad4
3190 3200 3210 3220 3230 3240 3250 3260 3270 3280
ON055368_H._taeniaeformis
FJ597547_H._taeniaeformis
JQ663994_H._kamiyai
NC037071_H._kamiyai
PP104554_H._kamiyai
LC008533_Hydatigera_sp._(France)
NC061206_Hydatigera_sp._(China)
MW808981_Hydatigera_sp._(China)
NC021141_H._parva
NC021142_H._kepkogorski
3290 3300 3310 3320 3330 3340 3350 3360 3370
tRNA-Gln
3380 3390 3400 3410 3420 3430 3440 3450 3460 3470
tRNA
Phe
3480 3490 3500 3510 3520 3530 3540 3550 3560 3570
Met
atp6

```
                                 atp6 3580      3590      3600      3610      3620      3630      3640      3650      3660      3670
                                 ------+---------+---------+---------+---------+---------+---------+---------+---------+---------+---
ON055368_H._taeniaeformis        AGAATTTTGTATAACAAGATAATAAAAAGTGATATGGTTTACTATTATTTAAGCATAATTTTGTGAACTATGTTATTATTTGTAAGTTATCGTTTTCCTT
FJ597547_H._taeniaeformis        ............................................................C....................A................C.
JQ663994_H._kamiyai              ..TG..........T........T......AG....A....T.....C.....T..T........G........G..G...A................A.
NC037071_H._kamiyai              ..TG..........T........T......AG....A....T.....C.....T..T........G........G..G...A................A.
PP104554_H._kamiyai              ..TG..C.A.....T........T...G.AAG.........T.....C.....G..T..C............C....G...T..................
LC008533_Hydatigera_sp._(France) ..TG....A..C.GT...G....T...G..AGC........T...........T..T..C..A..................A..................
NC061206_Hydatigera_sp._(China)  T.C.C.A.T.....T........T..TG..TCA..A..C..T...........T..................C........T.......C..A.......
MW808981_Hydatigera_sp._(China)  T.C.C.A.T.....T........T..TG..TCA..A..C..T...........T..................C........T.......C..A.......
NC021141_H._parva                .AT.AGA.T...TC---.T.TG.T.TGGAAA.GCG...A..T........T.AT.....AA..ATTGT.T.......G...T...C......G.....C.
NC021142_H._kepkogorski          ..CG..A.T..CTCT.....T..C...G..AGA..T.....T...........T........A..G..A......C.T...T...C............A.

                                 atp6 3680      3690      3700      3710      3720      3730      3740      3750      3760      3770
                                 ------+---------+---------+---------+---------+---------+---------+---------+---------+---------+---
ON055368_H._taeniaeformis        ATTGCTATAGTCCTTGATTGTTTATTGTATGATTAATAGGTATTGTTTTTGTATGCTTTGTTAGATTATGGTTGAATCGTTTATTAGATAATGTTAATAC
FJ597547_H._taeniaeformis        .C..T.....C.............................G..............T.......................A....................
JQ663994_H._kamiyai              ....T..............A...G................GG.............T.....A.....G..A..A.....G.....G......AGA.G.G.
NC037071_H._kamiyai              ....T..............A...G................GG.............T.....A.....G..A..A.....G.....G......AGA.G.G.
PP104554_H._kamiyai              ....T..................G....G.........A.G...........C.................AC.A.....A.......TCG..AG..GCG.
LC008533_Hydatigera_sp._(France) .C..T..............A........G......T.G..G....................G.....G...C.A.....A............AG.G..GT
NC061206_Hydatigera_sp._(China)  .C.....................G.G.CG.AT..GGGT..G...A..........T...A....G.....T..A.CC........G.GGG.......A..
MW808981_Hydatigera_sp._(China)  .C.....................G.G.CG.AT..GGGT..G...A..........T...A....G.....T..A.CC........G.GGG.......A..
NC021141_H._parva                ....T..........ATA.A...G.AA.G.TT..GG.TT..G........TCG..T...A....T....TT...GG....G..G.GAG.G....G....G
NC021142_H._kepkogorski          .C..T..C.......T.A.A...G..A...AT...G.T..AG.A........G..T........T.....A..A........G..TA.......AG...G

                                 atp6 3780      3790      3800      3810      3820      3830      3840      3850      3860      3870
                                 ------+---------+---------+---------+---------+---------+---------+---------+---------+---------+---
ON055368_H._taeniaeformis        TTTTTTTGCTAGATTTGTGCCATTGGGAACGCCACTTTATATTTGTCCTTTAGTATGTATAGCTGAGTCTATAAGTTATATCATACGTCCTTTTGTATTG
FJ597547_H._taeniaeformis        .................................T....................G..........................T..................
JQ663994_H._kamiyai              ..................C.....A..G......A................G....................T........A..............G..A
NC037071_H._kamiyai              ..................C.....A..G......A................G....................T......G.A..............G..A
PP104554_H._kamiyai              ...........................G..A..TA....C................................T..C...G.A.................A
LC008533_Hydatigera_sp._(France) .....................G..A..T..A...A...T.........AC....G...........A.....T........A..............G..A
NC061206_Hydatigera_sp._(China)  ..................T..G..A.....A..C....T.....................T......CT......G...G.T..............C..A
MW808981_Hydatigera_sp._(China)  ..................T..G..A.....A..C....T.....................T......CT......G...G.T..............C..A
NC021141_H._parva                A........A..T.....A...G.A..T..T...T.A.TC.................C.........CTGG.T..A...TGT.........G....T..A
NC021142_H._kepkogorski          ............T.........G.A..C..A...A.A.T.............................TG.....A...G.T........A.....T..A

                                 atp6 3880      3890      3900      3910      3920      3930      3940      3950      3960      3970
                                 ------+---------+---------+---------+---------+---------+---------+---------+---------+---------+---
ON055368_H._taeniaeformis        ATACTGCGGCCATTTATTAATATAAGTTTAGGTTGTTTTGGTGCTGCTTTTATTGGAGGAGCAGTAAATGGTAATTATTTGTGATTTTTAATATTGGTGT
FJ597547_H._taeniaeformis        ...........................................................G.........A.....................C....A.A.
JQ663994_H._kamiyai              ...........G.....A...........G..............C......G....C..G.T.....GC.T............G......G....A...G
NC037071_H._kamiyai              ...........G.....A...........G..............C......G....C..G.T.....GC.T............G......G....A...G
PP104554_H._kamiyai              ...T..........................................T....G.....A.G.T.....G.AT..................GG.T..A..A.
LC008533_Hydatigera_sp._(France) ............................................C......G....TA.G.TT....G..T.......C...........G........A
NC061206_Hydatigera_sp._(China)  ..TT.A.....G........C..T.....G..C........G....TA...G....T..GTTG..G...AT.....GA.GA......A.CG.....A.TG
MW808981_Hydatigera_sp._(China)  ..TT.A.....G........C..T.....G..C........G....TA...G....T..GTTG..G...AT.....GA.GA......A.CG.....A.TG
NC021141_H._parva                ..TA.A..T..T...G................A.............T.GC.T.G..TA.TTT.A..G.A.T...AGTA.GA..G.GAA..T.G..AT.TG
NC021142_H._kepkogorski          .................A..C...........A........C....T..A.G....C..TAT....T...T........GA..T...A..C.T.....CA
```

```
                              atp6 3980      3990      4000      4010      4020      4030      4040      4050        4060      4070
                              ------+---------+---------+---------+---------+---------+---------+---------+---------  -+---------+
ON055368_H._taeniaeformis     TATTATTTTTTTATGAAGTTTTTGTGGCTTTAGTACATTGATTTATTGTTCTTAGCATATTATTATTTTCTGAAGATCATTAGTG---TGTAATGTTTAT
FJ597547_H._taeniaeformis     .......................................................T.....G.......................---.A.G........
JQ663994_H._kamiyai           .........................A........T....................T.....GCAG.........A.........------........G.
NC037071_H._kamiyai           .........................A........T....................T.....GCAG.........A.........------........G.
PP104554_H._kamiyai           ...................A...................................T.....G.......................---.-..........
LC008533_Hydatigera_sp._(France) C........................A........T....................T.....GC.G....................---.-.G.....A..
NC061206_Hydatigera_sp._(China) CG..........................C..G..T.....G..C...........G...C........................TAGA.A.G..AG...C
MW808981_Hydatigera_sp._(China) CG..........................C..G..T.....G..C...........G...C........................TAGA.A.G..AG...C
NC021141_H._parva             .G.................G.....A...G.G..T...........A..G..C..T.....G..G.....C...A.C......A------.GG....GT.
NC021142_H._kepkogorski       .TC.T..................A.T........T..........................G.........AGG..........A---.A.-...A..G.

                              nad2    4080      4090      4100      4110      4120      4130      4140      4150      4160      4170
                              ---------+---------+---------+---------+---------+---------+---------+---------+---------+---------+
ON055368_H._taeniaeformis     AAATAGTCGATTAAATTTTAATACTGTATTTTTTTTTTCTTTTTTTTTTTCTGGCTTTTTTTGTTTATTGTGTTGTATTGTTGATAAATTAATAGGCTTT
FJ597547_H._taeniaeformis     .....................................CT...........................G.............................T...
JQ663994_H._kamiyai           T.G.........T..A....G...........................C...T.T...........G..A......G.A.................T...
NC037071_H._kamiyai           T.G.........T..A....G...........................C...T.T...........G..A......G.A.................T...
PP104554_H._kamiyai           T.........A....A....................................ATT..A.........C........GCG.................T...
LC008533_Hydatigera_sp._(France) C.G.........T..A.........A..........................A.TC.............A......G.G...........G.....T...
NC061206_Hydatigera_sp._(China) T....A......T.GG.........A.T.C....................G.T....G......C.GC....C........C........G..T..G...
MW808981_Hydatigera_sp._(China) T....A......T.GG.........A.T.C....................G.T....G......C.GC....C........C........G..T..G...
NC021141_H._parva             TCG.-..TA..---GA.A...ATA.T.T.A......C.T..A......C.T.A.T....A.AT...G....--.....G.......GG..G..T..G...
NC021142_H._kepkogorski       TT...A....A..GGA.......GAAA.A.........G...G......G.CT.T...........G.....C...G.A.................G...

                              nad2    4180      4190      4200      4210      4220      4230      4240      4250      4260      4270
                              ---------+---------+---------+---------+---------+---------+---------+---------+---------+---------+
ON055368_H._taeniaeformis     TGAATATTTTTAGAATTATGTGGTTTATCAATTATACCGTCATTTTTTTATAGTAAAAAGATTGGTTTATATAAATTTTATAGGTGTGTTTTGAGATATA
FJ597547_H._taeniaeformis     ............................................................G.......G..........................G..C.
JQ663994_H._kamiyai           .....T.................G.....T..A.....A..T..........A.......G..A...................TA..........T....
NC037071_H._kamiyai           .....T.................G.....T..A.....A..T..........A.......G..A...................TA..........T....
PP104554_H._kamiyai           ...G.............G.....G.....T..A.....A..T..........A.......G..A....G..............A..........CT....
LC008533_Hydatigera_sp._(France) ..GG.T.....G..G........G.....T..A.....A..T..........A.......G.............T.................A..G...G
NC061206_Hydatigera_sp._(China) ...G.G.....G.....G..C..GC....T..AG.G..T..C..C....T..ACCCT...G..AA.A.G............G.T.....AC.ATAC....
MW808981_Hydatigera_sp._(China) ...G.G.....G.....G..C..GC....T..AG.G..T..C..C....T..ACCCT...G..AA.A.G............G.T.....AC.ATAC....
NC021141_H._parva             ...G.T...........G.....GC.............T..T..........AATTT..AC..A..GGG..C...A.A...G...C.A..C.TTCT...G
NC021142_H._kepkogorski       .................G........G..T..AG.T.....T..........AA.....AG..A..C.........................A.......

                              nad2    4280      4290      4300      4310      4320      4330      4340      4350      4360      4370
                              ---------+---------+---------+---------+---------+---------+---------+---------+---------+---------+
ON055368_H._taeniaeformis     TTATAGTGTCTGGTTTATCTTCAGTTTTAGTGGTATTGGGGTTAATGTTTTTGGGTTTGTATTATTTTATTTATTTTGGGTTTGCGTTAAAGTTCGGTTT
FJ597547_H._taeniaeformis     ..............C................A....................................G.........................T.....
JQ663994_H._kamiyai           .A.....A........G......A.A..GA.A..G..A..T..G........A..G.............................A........T..G..
NC037071_H._kamiyai           .A.....A........G......A.A..GA.A..G..A..T..G........A..G.............................A........T..G..
PP104554_H._kamiyai           .A.....A.....A.........A.A...A.A..CC.A..T...........A.........................................T.....
LC008533_Hydatigera_sp._(France) .A.....A.....G........TA.A...A.A........T...........A...............G.........................T..A..
NC061206_Hydatigera_sp._(China) ...................C..T.....TA.A..G..A..T...........AA..........C...G................TC.G.....T..G..
MW808981_Hydatigera_sp._(China) ...................C..T.....TA.A..G..A..T...........AA..........C...G................TC.G.....T..G..
NC021141_H._parva             .A....................GA.A..TA.A.....A..T...T.AA...CTA.A..A.........G..CT......T...ATA..G...C.T.....
NC021142_H._kepkogorski       ..G..A.T.....AC.G.....T.....TA.A.....A..............A.AG.....C......G..........A.....T..T.....T..G..
```

```
                                 nad2  4380      4390      4400      4410      4420      4430      4440      4450      4460      4470
                                 ---------+---------+---------+---------+---------+---------+---------+---------+---------+---------+
ON055368_H._taeniaeformis        ATTCCCATTTTTGTTGTGGGTGTATCGAGTGTTTAGTGTAGGAAATTGGTTATTTATTTTTTTTTTAAGAGTTTATTAAAGTTTCCGGTTTTATTTTTTT
FJ597547_H._taeniaeformis        ...T...............A.A........A........T.............................G.................A............
JQ663994_H._kamiyai              G..T..G.......................A.....G..T..T.....A....................T........G........C..A..G......
NC037071_H._kamiyai              G..T..G.......................A.....G..T..T.....A....................T.................C..A..G......
PP104554_H._kamiyai              G..T..T...CAA..A..A..A..............G..T..T..C..A........A........G....................A............
LC008533_Hydatigera_sp._(France) G..T........A.......................G..T..T..C.....G...G..........G..T.................A.....G......
NC061206_Hydatigera_sp._(China)  G..T..T.....A..A.....T..C.A...T......A.T..T........G.....C...................................G......
MW808981_Hydatigera_sp._(China)  G..T..T.....A..A.....T..C.A...T......A.T..T........G.....C...................................G......
NC021141_H._parva                ...T..T...A...GA.....T...T....A...GCGAGCA.T..A.....GC....A......A........G..A.G........AA....G......
NC021142_H._kepkogorski          G...........T..A..A..T....A...T.....AA....T.....A..G..C........C.....G...G....G........A............

                                 nad2  4480      4490      4500      4510      4520      4530      4540      4550      4560      4570
                                 ---------+---------+---------+---------+---------+---------+---------+---------+---------+---------+
ON055368_H._taeniaeformis        TGTTTTTTATACCAAAATGTTAATGTTAAATTGGTTTATGTTGATTGTTTTTTGACTATTCTTCTTTGTTCTATTTTTATTTGATTTTTTAGCTTATCTT
FJ597547_H._taeniaeformis        ...........................................................................C................T.......
JQ663994_H._kamiyai              ......C.T..T....................A....................A......T..T.A........C........G........TC.T....
NC037071_H._kamiyai              ......C.T..T..G.................A....................A......T..T.A........C........G........TC.T....
PP104554_H._kamiyai              ......C.T.......................A......A.......C.....T......T..T........G.....G.A...........TC.T....
LC008533_Hydatigera_sp._(France) ......C.T..T..G...A.....A.......A.....CA.............A......T..T.G......G...................TC.T....
NC061206_Hydatigera_sp._(China)  ......C.T..T..G.CA.A....A.A..G.....C...A.............A...T.G..GA.......A.....GG.............T...A...
MW808981_Hydatigera_sp._(China)  ......C.T..T..G.CA.A....A.A..G.....C...A.............A...T.G..GA.......A.....GG.............T...A...
NC021141_H._parva                ....A......TG.GTTGAGG..A..G..GA.T.....CA.A...A.A...C.C......T.GA.A......T.AG..T....G........T..G..G.
NC021142_H._kepkogorski          ......C.C..T....TG.A....AGA..G..A....T...A..................T.G..A...........C..............AC......

                                 nad2  4580      4590      4600      4610      4620      4630      4640      4650      4660      4670
                                 ---------+---------+---------+---------+---------+---------+---------+---------+---------+---------+
ON055368_H._taeniaeformis        GAGAATATGTTTGGTGTCATATTTCTTTGTCTTCTATTGCTACTCTGGTGGTAGCATGCTTTTGTAGAAGAGTTGAAATTTGTTTTTTTATATATTTGTA
FJ597547_H._taeniaeformis        ....G...........C...........................T.............T........T................................
JQ663994_H._kamiyai              ..A.G..C.....A..C........A.........G........T.A..A..T.....T........T..G..........................A..
NC037071_H._kamiyai              ....G........A...........A.........G........T.A..A..T.....T........T..G..........................A..
PP104554_H._kamiyai              ....G........A...........A..A......G........T.A..A..T..C..T........T..GA............................
LC008533_Hydatigera_sp._(France) .G..G........A...........G.........G........T.A.....T.....T........C..G....................T.....A..
NC061206_Hydatigera_sp._(China)  ....G.....G...........A..A.........G.......GT.......G.....T........C..TA.A.....C................GA..
MW808981_Hydatigera_sp._(China)  ....G.....G...........A..A.........G.......GT.......G.....T........C..TA.A.....C................GA..
NC021141_H._parva                .G..G.....A..A..C........G..A..A..AG........T.AA.A.....G..T........G.....A..G.......G..........GGT..
NC021142_H._kepkogorski          ....G........A........A..A..A......G.............A........T........T..GT.......A..C............G....

                                 nad2  4680      4690      4700      4710      4720      4730      4740      4750      4760      4770
                                 ---------+---------+---------+---------+---------+---------+---------+---------+---------+---------+
ON055368_H._taeniaeformis        CTATTTTTTTTGAGCTACACTTACCATAATTTATTTGAAAGTTATTTCTGATATGGTTGATTTGAAGAGTTTTATGTTTTGAGGATTTTGTTTTTTATTA
FJ597547_H._taeniaeformis        ............G..................................................A....................G.....C.........
JQ663994_H._kamiyai              T...........G..A..GT....T...G.........GTA..G........................................T...........G...
NC037071_H._kamiyai              T...........G..A..GT....T...G.........GTA..G........................................T...........G...
PP104554_H._kamiyai              ............G..A......GTT...T..........TA..G...................A....................G...........G...
LC008533_Hydatigera_sp._(France) T...........G...........T...G.........GTA..............A.......A....................G...............
NC061206_Hydatigera_sp._(China)  ............G..CT.TT.AGTT...........A.GGA...........................................T...............
MW808981_Hydatigera_sp._(China)  ............G..CT.TT.AGTT...........A.GGA...........................................T...............
NC021141_H._parva                T......G.G....G.TGTG....A...G....C..T..CT.A...G.....G.TA.G..AA.T.......A.C.......G.TT.....C........G
NC021142_H._kepkogorski          T.......................T..T........A.G.A..............A.A.....A.................G.TG.....C.....G...
```

```
                                 nad2   4780      4790      4800      4810      4820      4830      4840      4850      4860      4870
                                 ---------+---------+---------+---------+---------+---------+---------+---------+---------+---------+
ON055368_H._taeniaeformis        TTAATAACTCCAGTTTCTGTACCTTTAATCTATAAGATTAGAGTGTGTATTGGTATATTATACTCTTCAATATATTTGCTGTTGATATGAAGAGTTTATA
FJ597547_H._taeniaeformis        ............A.....................................C.....T.....................T....A........G.......
JQ663994_H._kamiyai              ..G........TA.......T...C....T...........G...A.....A.......G..T.....TG.....A.AT....A..T........G....
NC037071_H._kamiyai              ..G........TA.......T...C....T...........G...A.............G..T.....TG.....A.AT....A..T........G....
PP104554_H._kamiyai              ...........CA.......T........T....................A........G..T.....CG.G...A..T.A..A..T........G....
LC008533_Hydatigera_sp._(France) ...........TA.......T........T........C..G.................G..T.....TG.G...A.A..A.....T..G.....A....
NC061206_Hydatigera_sp._(China)  ............A.......TT.A...G.............G..T...G.......T.....T......G....C...T.A..AG.C..G..........
MW808981_Hydatigera_sp._(China)  ............A.......TT.A...G.............G..T...G.......T.....T......G....C...T.A..AG.C..G..........
NC021141_H._parva                ..GG.......TT.A...A.G..A..G..T............A.......A..C..T..G..T........T...A.AT....AG.G.....T..G....
NC021142_H._kepkogorski          ..G........T........T..A.................G......G.A......C.T..T..A..GG.T...A.ATA..A................

                                 nad2   4880      4890      4900      4910      4920      4930      4940      4950            4960
                                 ---------+---------+---------+---------+---------+---------+---------+---------+--      -------+----
ON055368_H._taeniaeformis        GTTTTTCTGAACAGTTTTTCTTATATAAGCTAGCTAGTGGTTATTTTTTTAGTGATGTTTATAAAAATTGGTTAAATTAATT------GTAAAGTAGTTT
FJ597547_H._taeniaeformis        ......................................................G........................G..------............
JQ663994_H._kamiyai              ...................T.........T........A.............AAGG........T.G...A..G..A..G..------..G.T.......
NC037071_H._kamiyai              ...................T.........T........A.............AAGG........T.G...A..G..A..G..------..G.T.......
PP104554_H._kamiyai              .............................T.G..C....A............GAGG........T.G......G.....G..------....T.......
LC008533_Hydatigera_sp._(France) ................C..T..G......T......................AAGA........T.G...A.....A..GA.------....T.......
NC061206_Hydatigera_sp._(China)  .G..............C..TC.G......T.....G....G.G.........AATG........T..........G.GG..G..------....T.......
MW808981_Hydatigera_sp._(China)  .G..............C..TC.G......T.....G....G.G.........AATG........T..........G.GG..G..------....T.......
NC021141_H._parva                .A........G........T.........T.G.......AG..C....A.GTGA.A....T....GG...AG.T......A.-TTAGG....T.......
NC021142_H._kepkogorski          ....C...........C..T.....C...T.....................AGA.A.....C..T..C..A....GA....-------....T.......

                               tRNA 4970  Val  4980      4990         5000      5010      5020   tRNA 5030  Ala 5040      5050
                                 -----+- --------+---------+--------   -+---------+---------+--  -------+---------+---------+--------
ON055368_H._taeniaeformis        ATAAAAA-TGTCTGTTTTACACACAGGAGAACTCG---TTTGAGCTTTACTATGATTTAGTT--TAACAAAATAGTTTAATGAAAAAATATTGGGTTTGC
FJ597547_H._taeniaeformis        .......-...........................---................G.......--....................................
JQ663994_H._kamiyai              ..T....A..........................A---..............A-G....A..GT.................-...........A......
NC037071_H._kamiyai              ..T....A..........................A---..............A-G....A..GT.................-...........A......
PP104554_H._kamiyai              ..T....-..............G...........A---..............A-....TA..GT.................-...........A......
LC008533_Hydatigera_sp._(France) ..T....A..........................A---..............AAG....A..GT.................-...........A......
NC061206_Hydatigera_sp._(China)  ..T....-.A.T..............A.......T---..............G--C...AC.---.....G..........T..................
MW808981_Hydatigera_sp._(China)  ..T....-.A.T..............A.......T---..............G--C...AC.---.....G..........T..................
NC021141_H._parva                ...T...A...T.............A........TAGTCA..........G..AG---..A.---.....T........-.-G......G..........
NC021142_H._kepkogorski          .......-..............G...........A---..G.............A------A..---..............-G..........A......

                                5060      5070      5080       5090        5100      5110  tRNA 5120  Asp 5130      5140        5150
                                 -+---------+---------+--------- +----  -----+---------+ ---------+---------+---------+----  -----+--
ON055368_H._taeniaeformis        GTCTCAAAGATGGATTGTGGTTCTGTTGTTA-TATTT--GTAATTTTAGTTTAAG-AATAAAATGATGATTTGTCTAGTCATAGATGATA--TAAATATC
FJ597547_H._taeniaeformis        ...............................-.....--...............-........AG........................--........
JQ663994_H._kamiyai              ...............................G.....--...............T........AG........................--........
NC037071_H._kamiyai              ...............................G.....--...............T........AG........................--........
PP104554_H._kamiyai              ...............................AC....--...............TG.......AG.......................G..--......C.
LC008533_Hydatigera_sp._(France) ...............................G.G...--...............T........AG........................--........
NC061206_Hydatigera_sp._(China)  ...............AAATA...........-.GC..--..G.C..........T--G.......G..G.........C..........---.T.T....
MW808981_Hydatigera_sp._(China)  ...............AAATA...........-.GC..--..G.C..........T--G.......G..G.........C..........---.T.T....
NC021141_H._parva                .......G....A.G...TT...........-.....AA..G.C..........CT.--.....................G.T...A..CTTGTG.G..T
NC021142_H._kepkogorski          ................TACA...........A..C.---...............---......TG....C...................TA.G......
```

```
                                      5160      5170 nad1 5180      5190      5200      5210      5220      5230      5240      5250
                                 --------+- -------+---------+---------+---------+---------+---------+---------+---------+---------+-
ON055368_H._taeniaeformis        AAGTTACTT-TAGTAATGATTATTTTTACTTTTATATCTGGTTTGTTTGGATTATTGATAAGTTTATTGGTGATAGCTTTTTTTATATTAGGTGAACGTA
FJ597547_H._taeniaeformis        .........-.G................................A.......................A...............................
JQ663994_H._kamiyai              ........CA..AC...................T.G........A.....G..G...................................G.....G....
NC037071_H._kamiyai              ........CA..AC...................T.G........A.....G..G...................................G.....G....
PP104554_H._kamiyai              ..A..G...A.---T......G.............G..............G..G..A........G..A....................G.....G....
LC008533_Hydatigera_sp._(France) .........A..A............................C........G..G........C.....AA.T.................G.....G....
NC061206_Hydatigera_sp._(China)  .G.......G.---T...T.GG.G...GT...............A.....G...C.T.....G..G..AA.A.....C......................
MW808981_Hydatigera_sp._(China)  .G.......G.---T...T.GG.G...GT...............A.....G...C.T.....G..G..AA.A.....C......................
NC021141_H._parva                .....G...------...T.AC.G...GAA..AT.T......A.TA....T........T.....G..A..A..............TC.T..G..G....
NC021142_H._kepkogorski          .........AA---.G..G.AT.G...GTC..............A..A.....GC.TG..........A..T..............T........G....

                                 nad1 5260      5270      5280      5290      5300      5310      5320      5330      5340      5350
                                 --------+---------+---------+---------+---------+---------+---------+---------+---------+---------+-
ON055368_H._taeniaeformis        AGATATTAGGATATTCTCAATATCGTAAGGGTCCTAATAAGGTTGGTTTTTTAGGATTGTTTCAAAGTTTTGCTGATTTATTGAAGTTAATTGTGAAGTT
FJ597547_H._taeniaeformis        ..........G....................C................................G..............G....................
JQ663994_H._kamiyai              .....C.T..G...................................GA..A....G...........C..............A...........C.....
NC037071_H._kamiyai              .....C.T..G...................................GA..A....G..........................A...........C.....
PP104554_H._kamiyai              .....C.T...........G..C.......................AA..A....G..A.......................A.................
LC008533_Hydatigera_sp._(France) .....C.T..G....................A...............A..A.......A.......................A...........T.....
NC061206_Hydatigera_sp._(China)  .....C....G........G...........C.....A........AG....G..T..A.....G..G.....A.....G..A.....GG....T.....
MW808981_Hydatigera_sp._(China)  .....C....G........G...........C.....A........AG....G..T..A.....G..G.....A.....G..A.....GG....T.....
NC021141_H._parva                ....T.....T..........T.........A..C...........GA..G.T..G........G..G.....A...............G....T.....
NC021142_H._kepkogorski          ....T..G..G....................A.....A.........A.C..G..T..A........G..............A......G....T.....

                                 nad1 5360      5370      5380      5390      5400      5410      5420      5430      5440      5450
                                 --------+---------+---------+---------+---------+---------+---------+---------+---------+---------+-
ON055368_H._taeniaeformis        TAAGTGGTTTTATTTTCAAAGTCGTAATTTTGTTGGTTTAATTGGTGTAATGTTATTAGTTTTATTATCTATATTTTATTGTTTTGTTTATGGTGATTAT
FJ597547_H._taeniaeformis        ........................................G.............G.............................................
JQ663994_H._kamiyai              ..........................G....A.A..C..........................G..G...G.GC...........A..............
NC037071_H._kamiyai              ..........................G....A.A..C..........................G..G...G.GC...........A..............
PP104554_H._kamiyai              C.....A..............C....GA...A.......T.................G............G.GC...........A..............
LC008533_Hydatigera_sp._(France) ......A...........G.......G....A......................G........G......G.GC...........A..............
NC061206_Hydatigera_sp._(China)  ....CA..A...C.....T.......C.A....G......T..............C.GT.AA.G........T.....C.....................
MW808981_Hydatigera_sp._(China)  ....CA..A...C.....T.......C.A....G......T..............C.GT.AA.G........T.....C.....................
NC021141_H._parva                ....ATT.A..T.......G......GAG.AA....GG.T..A..A..T...C.T..G.....T..GGT...T...........CT........A.A.T.
NC021142_H._kepkogorski          ....AAA.........A.........G....A....G....C....AC.T....GC.G...A.G..GA.A...............A............T.

                                 nad1 5460      5470      5480      5490      5500      5510      5520      5530      5540      5550
                                 --------+---------+---------+---------+---------+---------+---------+---------+---------+---------+-
ON055368_H._taeniaeformis        TATGGGTCAAAGATAGGTGAGTTTTCAATGCTTTGATTTTTGGTTATTACCAGTTTATGTGGTTATGCTGTGTTATGTGCTGGTTGAGGTAGTTTTAATA
FJ597547_H._taeniaeformis        ...................G...............G..............T.............................C.....G.............
JQ663994_H._kamiyai              .....AC.T...G.T...C..C.............G.........G....A..G..G...........C.........A.......G...G.........
NC037071_H._kamiyai              .....AC.T...G.T...C..C.............G.........G....A..G..G...........C.........A.......G...G.........
PP104554_H._kamiyai              ..C...G.T...G.T...C..C....C........G..............G..A..G.....A...............A.......G...........A.
LC008533_Hydatigera_sp._(France) .....AC.T..AG.T...C..C.............................A..G........A........A......A...........G..C....C.
NC061206_Hydatigera_sp._(China)  ............G.TA...G...G..T...T.G..G..............A...........G........AC.G...A...............G.....
MW808981_Hydatigera_sp._(China)  ............G.TA...G...G..T...T.G..G..............A...........G........AC.G...A...............G.....
NC021141_H._parva                ...A..GGTG.AT.GAA...TG.G...T..T.A............G.G..T.....G...A.A...T..A.A..G....TA.....G.......A.....
NC021142_H._kepkogorski          .....A..T...C.TAAA..TC....T...T.G..G..A..A........T..A........G...........G.A.ATA...........A.C.....
```

```
                                  nad1  5560      5570      5580      5590      5600      5610      5620      5630      5640      5650
                                  --------+---------+---------+---------+---------+---------+---------+---------+---------+---------+-
ON055368_H._taeniaeformis         ATTATTCATTTTTAAGTTGTATCCGTTGTAGGTTTAGATCTATAAGATTTGAAGCGTGTTTTATGAGTATAATTATCTTTAGGGGTTTATGTTATAAAAG
FJ597547_H._taeniaeformis         ...............................A.....G........G.............................T.......................
JQ663994_H._kamiyai               .............G..C.C...T........A.........G................C.................T....TA........C........
NC037071_H._kamiyai               .............G..C.C...T........A.........G................C.................T....TA........C........
PP104554_H._kamiyai               ..................C...T........A.........G....T.........A..............G.C..T.....A.....G..C........
LC008533_Hydatigera_sp._(France)  ......................T........A.....G...G..........G...T................A..T.....A.....G...........
NC061206_Hydatigera_sp._(China)   .......TC..A....A.C...T..............T...G....G.........T...........T..G....T.....T..G..G.....C..T..
MW808981_Hydatigera_sp._(China)   .......TC..A....A.C...T..............T...G....G.........T...........T..G....T.....T..G..G.....C..T..
NC021141_H._parva                 .......T.....G....CA..T......GCT...G.T...G.T............T.........G..T.......A...T.T.............TGTG.
NC021142_H._kepkogorski           .......T..............T........A..CG.G..A...........G...A.........G..T.T..A..T.....A..........TA.GTG.

                                  nad1  5660      5670      5680      5690      5700      5710      5720      5730      5740      5750
                                  --------+---------+---------+---------+---------+---------+---------+---------+---------+---------+-
ON055368_H._taeniaeformis         ATATAAATTGAGGGATTTAATAAATGTTGATTGAGTTTCTTTATTTATATTTCCTTGTGCTTATATAGTATTTTTAATAGGTATATTATGTGAAACTAAT
FJ597547_H._taeniaeformis         .........A.........................C...............................A................................
JQ663994_H._kamiyai               G........T..A.....G....G...........C.....................C......G.TA.......GG.T......C..............
NC037071_H._kamiyai               G........T..A.....G....G...........C.....................C......G.TA.......GG.T......C..............
PP104554_H._kamiyai               G..C.....T........G....G..C.......A........C....................G.TA.T......G.T.....G..G............
LC008533_Hydatigera_sp._(France)  G........T..T.....GG...G.AC.......A.G......A....................G.TA.......GG.T.....G..G............
NC061206_Hydatigera_sp._(China)   G....GT..A.........TG.............A.......G.................C...G..A....C..G.TA...........C.........
MW808981_Hydatigera_sp._(China)   G....GT..A.........TG.............A.......G.................C...G..A....C..G.TA...........C.........
NC021141_H._parva                 G.....T..ATT......T.GC.GAAG..GG..GTC....G.T.A.T.......A....T......TC.T.....GG.G..G..G..G.......A...
NC021142_H._kepkogorski           G.........GTT...G...G.............G..GC....G..G..T.....A...AT.............C.G...A.A........C.........

                                  nad1  5760      5770      5780      5790      5800      5810      5820      5830      5840      5850
                                  --------+---------+---------+---------+---------+---------+---------+---------+---------+---------+-
ON055368_H._taeniaeformis         CGTACTCCGTTTGATTATGGTGAAGCTGAAAGAGAATTGGTTAGTGGATTTAATGTTGAATATAGTGGTATATATTTTACTTGTTTGTTTGCTTGTGAAT
FJ597547_H._taeniaeformis         ............................................C.......................G................A...........G.
JQ663994_H._kamiyai               ................................G..G.......................G........................................
NC037071_H._kamiyai               ................................G..G.......................G........................................
PP104554_H._kamiyai               ........A..C....................T..G..A.....C..............G.....G..................................
LC008533_Hydatigera_sp._(France)  ..........................G.....G..G.......................G..C...................................G.
NC061206_Hydatigera_sp._(China)   ........T.................C..G.....G........A..G........A......T....A..T....................C.......
MW808981_Hydatigera_sp._(China)   ........T.................C..G.....G........A..G........A......T....A..T....................C.......
NC021141_H._parva                 ..C..G..A...........A........G.....G..A........T........G..G...........T........G..C........A.....G.
NC021142_H._kepkogorski           ..G.....A...........G..G..A........G..A..A..A...........A..G...........T......................C....

                                  nad1  5860      5870      5880      5890      5900         5910      5920      5930      5940
                                  --------+---------+---------+---------+---------+---   ------+---------+---------+---------+--------
ON055368_H._taeniaeformis         ATATAATTATATTTATTTTTTCTTGAATAGGGGTGATTTTATTTTTAGGTGA---CAGTATATTTAGCATGATTTTATTATTTATAAATTTGTTATTTTT
FJ597547_H._taeniaeformis         ......................................A..........G..---T.......................G....................
JQ663994_H._kamiyai               ....T..C..................G.T..A..AG.G...........A.G---GG..G.....G.G...G.......G...T.T......AC.......
NC037071_H._kamiyai               ....T..C.....C............G.T..A..AG.G...........A.G---GG..G.....G.G...G.......G...T.T......AC.......
PP104554_H._kamiyai               ....T...................GG.T..C..AG.A..G........A..---AG.CG....AG.TT.A.....G..G...T.T........T.....
LC008533_Hydatigera_sp._(France)  ....T.....................G.T.....AG.G...........G..---AG..G.......T...........G...T.T.....A..G.....
NC061206_Hydatigera_sp._(China)   ..G.T..A........A..............T..TG.G.......GT...A.---A..GT....TCGGAAG..C..C......T.T..C.A.........
MW808981_Hydatigera_sp._(China)   ..G.T..A........A..............T..TG.G.......GT...A.---A..GT....TCGGAAG..C..C......T.T..C.A.........
NC021141_H._parva                 ....TG....GG..G...........G.T.....G........A..T..GAGTGTAG..T.G...G.TTGAG.GC.T..T...T.T......AC.T.....
NC021142_H._kepkogorski           ....T.....T...............GC.T..A..........C.GT...A.---AT..G..C....AGAAT.A...C.....T.T...C..........
```

```
                                  5950      5960      5970      5980      5990      6000      6010      6020      6030      6040    nad1
                                  -+---------+---------+---------+---------+---------+---------+---------+---------+---------+--------
ON055368_H._taeniaeformis         TATGTGGGCGCGTGCTACATTACCACGTGTACGTTATGATTTTTTTGTGAAATTTTTTTGAGAAATAGGTTTGTTAATGGTTATATTTAGCTTATTTTGT
FJ597547_H._taeniaeformis         ................................................T...............G.....C.............................
JQ663994_H._kamiyai               .........T..G..C..G...........T.................T..T...........G..T.....A..G....C......A..A..G..C...
NC037071_H._kamiyai               .........T..G..C..G...........T.................T..T...........G..T.....A..G....C......A..A..G..C...
PP104554_H._kamiyai               ......A..T..G........G..C.............................C.....G..GG.T..CC.............T.....G......A.C
LC008533_Hydatigera_sp._(France)  .........T..G.....G..G........C.................T..............G..T........G....C.........A.........
NC061206_Hydatigera_sp._(China)   ......AT.T........G.....G.....T..........A......A...C.......G.G.G.T..GG.T......T.G.....A..TC........
MW808981_Hydatigera_sp._(China)   ......AT.T........G.....G.....T..........A......A...C.......G.G.G.T..GG.T......T.G.....A..TC........
NC021141_H._parva                 ......A..T.................G.....G.......A....A.T.................T.....AAGTT.AT....T..A..A..T......
NC021142_H._kepkogorski           .........T..A.....T...........T................................GG.T.....A...T..C..G.G..A..T..C......

                                  6050      6060      6070   tRNA 6080 Asn  6090      6100      6110      6120      6130          6140
                                  -+---------+---------+--   -------+---------+---------+---------+---------+---------+-   --  ------+
ON055368_H._taeniaeformis         ATAGTAAATTAAGTCTATATAGAT---TAAATGTAAATCGTGATGCTGTTAACTTCAAGAAATAGTTGTTACTATTATAGTCGTTA---CA--TTCTTAT
FJ597547_H._taeniaeformis         ........................---...........................................................---..--.......
JQ663994_H._kamiyai               G....G..C..G............TAT.....A.....................................G...............GTTT---.......
NC037071_H._kamiyai               G....G..C..G............---.....A.....................................G...............GTTT---.......
PP104554_H._kamiyai               ........A..G............---...G.A...................................................G.GTT.G--.......
LC008533_Hydatigera_sp._(France)  G.G........G............---..........................................................GGTTT.--.......
NC061206_Hydatigera_sp._(China)   .....T..C..G............---.......................................AA..G.............GTG--.T--.......
MW808981_Hydatigera_sp._(China)   .....T..C..G............---.......................................AA..G.............GTG--.T--.......
NC021141_H._parva                 G.G..T.....G..T.G.C..TG.AGA.T.-.AA.............................G.A.T.AT.CG..G.......C.ATATG---......
NC021142_H._kepkogorski           ..T.....A..G............---..GGATA..........................G.....G.A..............AG.AATGGAT.......

                                tRNA Pro 6150      6160      6170      6180      6190      6200      6210      6220 tRNA 6230 Ile  6240
                                  ---------+---------+---------+---------+---------+---------+---------+---------+---------+---------+
ON055368_H._taeniaeformis         CTTAGTTTAAAAAGAATGATGGTTTTGGGGACCTTTGGTCTCAGTGAGAGATTTGGTTAATAGGGCTGCGAAGCAGGTTACTTTGATATAGTAAATAGTG
FJ597547_H._taeniaeformis         ....................................................................................................
JQ663994_H._kamiyai               .....................................................................AT.............................
NC037071_H._kamiyai               .....................................................................AT.............................
PP104554_H._kamiyai               ......................................................................T.............................
LC008533_Hydatigera_sp._(France)  ......................................................................T.............................
NC061206_Hydatigera_sp._(China)   .......................................................A.............TT.......C.............G...G...
MW808981_Hydatigera_sp._(China)   .......................................................A.............TT.......C.............G...G...
NC021141_H._parva                 ..........T............................................A.............AT.........................T...
NC021142_H._kepkogorski           .......................................................A.............AT.......C.............G.......

                                           6250      6260     tRNA-Lys 6270       6280      6290      6300        6310      6320
                                  ---------+---------+-       --------+--------  -+---------+---------+------- --  +---------+--------
ON055368_H._taeniaeformis         AATTGTTATTCCGTTAATATG-------ACTCATATATCTTATTA--AAAGTACTAGGTTCTTACCCTAGAGATGT-AT--TATACTATGGGTGGAGTAT
FJ597547_H._taeniaeformis         ....A................-------.................--.............................-..--...................
JQ663994_H._kamiyai               ....................T-------G...G...........T--.............................-..--AT...........T-....
NC037071_H._kamiyai               ....................T-------G...G...........T--.............................-..--AT...........T-....
PP104554_H._kamiyai               ....................T-------..C.GC..........T--.............................-..--.T....G......T-.A..
LC008533_Hydatigera_sp._(France)  ....................T-------G...............T--.............................-..--.T....G......T-....
NC061206_Hydatigera_sp._(China)   ...AT...............TT-----T......G.......A.T--.....G...............G.......-G.GT.TC...G......T-A...
MW808981_Hydatigera_sp._(China)   ...AT...............TT-----T......G.......A.T--.....G...............G.......-G.GT.TC...G......T-A...
NC021141_H._parva                 ....-...............TAGTTAGTGT....G.....A.G.TGT.......................GA....G..GTGTC..CG....CTT---..
NC021142_H._kepkogorski           ......A.............T-------......G........A.--.....GT...............A......-G.--.C...C...A...T-AA..
```

```
                                   6330      6340      6350      6360      6370      6380      6390      6400      6410      6420 nad3
                                   -+---------+---------+---------+---------+---------+---------+---------+---------+---------+--------
ON055368_H._taeniaeformis          GTTATTGGTTGGTTTTTCTTTATTTATTTTATTAAGTTTTGTTATTGGCTTTTTTTGTTGCAGGTTATTAAAAAAGATGGTTAGATTTGAAGGAGGATGG
FJ597547_H._taeniaeformis          ........C.........................G..A....................A...........G.....TG........G......
JQ663994_H._kamiyai                ...G..A...A.G...T............G.......GT......T..........TG.T..............TA.......A...T.A....A
NC037071_H._kamiyai                ...G..A...A.G...T............G.......GA......T..........TG.T..............TA.......A...T.A....A
PP104554_H._kamiyai                .A.........C....T............G...A..A........G..........TG.T..G............A...AG...A...TGA.....
LC008533_Hydatigera_sp._(France)   ....G.A...A.G...T...........G..G.....AA......T..........T..T...C.........AA....G...A..AT.A....A
NC061206_Hydatigera_sp._(China)    ......AA.G..G...ATA..G....G.......T.CA.A..G..A..T..........TG.T..G.....T...G.TA..GA....A...TTA.G..A
MW808981_Hydatigera_sp._(China)    ......AA.G..G...ATA..G....G.......T.CA.A..G..A..T..........TG.T..G.....T...G.TA..GA....A...TTA.G..A
NC021141_H._parva                  ....G.TAC.A.G....TGG.T....C...G..G.TGC.GA.A...TAT......AA..CTG.TA.T.....T....A.....TA.G..TTTTTCT...
NC021142_H._kepkogorski            ...T..AAGA.....AGTC..G....G...G..G.TGA..T.A.....T..........T..A...C.T...G.A...A...AGAC.A..ATTA.T...

                                   6430      6440      6450      6460      6470      6480      6490      6500      6510      6520 nad3
                                   -+---------+---------+---------+---------+---------+---------+---------+---------+---------+--------
ON055368_H._taeniaeformis          CCTAGATGTTATGAATGTGGATTTTTTAAAGGTTTGATGAAACTTAAATGTTTCAGATTTACTTACTTTGATTTGTTAGTAGTTTTTGTTATTTTTGATT
FJ597547_H._taeniaeformis          ....................G..............................T........................G..................C
JQ663994_H._kamiyai                T.A.............C..G.............A.....A.........T..G.......T.......A..G..G..........A.......
NC037071_H._kamiyai                T.A.............C..G.............A.....A.........T..G.......T.......A..G..G..........A.......
PP104554_H._kamiyai                T.A.................G........A....A.....A.........T..........T...G........GA..........A.......
LC008533_Hydatigera_sp._(France)   T.G.............C..G.............A.....A.........T..........T.......A....G..........A.......
NC061206_Hydatigera_sp._(China)    TT...........G....G..................T..........TG.TC.A.....T...A.........G..G.....AG.A.......
MW808981_Hydatigera_sp._(China)    TT...........G....G..................T..........TG.TC.A.....T...A.........G..G.....AG.A.......
NC021141_H._parva                  TG...T.A.....G....G.......T..CA.AGAA..TG.G..T.....T..G.......T...TC...A....GA........G.........
NC021142_H._kepkogorski            T....T.....C.......T......GG.....A................T..TG..........A.C.....A....G.....A..A.......

                                   6530      6540      6550      6560      6570      6580      6590      6600      6610      6620 nad3
                                   -+---------+---------+---------+---------+---------+---------+---------+---------+---------+--------
ON055368_H._taeniaeformis          TAGAAATATCTTTGTTGTTAAACATGCCTACTCAAGGGTTAATGTATTGAAGTTTTGTAGGGTATTATACATTTTTATGTATGTTGTTATTTGGATTTTT
FJ597547_H._taeniaeformis          ......................T............A.........G.......................................A..........G.....
JQ663994_H._kamiyai                ..........A..A..A..G..T.....G.....G..A.........G......TGT.....C....TG.....G........A...G.A..T.....
NC037071_H._kamiyai                ..........A..A..A..G..T.....G.....G..A..G......G......TGT.....C....TG.....G........A...G.A..T.....
PP104554_H._kamiyai                ..........A..A..........T..........G..A..T.......G......TGG..A..C..C.TG.....G..C.....AGC.G.....C.....
LC008533_Hydatigera_sp._(France)   ..........A.....A.....T.....A.....G..A..T..............TGC..A.......TG..................GA....T.....
NC061206_Hydatigera_sp._(China)    ....G..T..A..A....C.....T.....GGTA..G..T..............AA..CTGC.........TT...C..G.T.T.T..AGC.GCA..G...GC
MW808981_Hydatigera_sp._(China)    ....G..T..A..A....C.....T.....GGTA..G..T..............AA..CTGC.........TT...C..G.T.T.T..AGC.GCA..G...GC
NC021141_H._parva                  ....GG.C..A.....A..G..A.......TT....G.AT..GT.A.....G.A......T..T..C...TGC...A.TATAT.A......TA....T.....
NC021142_H._kepkogorski            ....G..........A............A.....................G......TGT........CGTG.....GA.A..T..A..GG.A..G.....

                                   6630      6640      6650      6660 nad3          6670 tRNA 6680 Ser  6690      6700      6710      6720
                                   -+---------+---------+---------+------ --       -+---------+---------+---------+---------+---------+-
ON055368_H._taeniaeformis          AATTGAGGTTTTTTTTGGTTATGTTAAGTGAATTTATTAG-------AGAAATGTATAAAGTTACTGCTAATAATTTTTTGTCAATTTGTTTTGGCTTTC
FJ597547_H._taeniaeformis          .......A......................G.........-------.........G..............................A..........
JQ663994_H._kamiyai                .......A....C.................G.........-------......A.G.G.............................A..........
NC037071_H._kamiyai                .......A....C.................G.........-------......A.G.G.............................A..........
PP104554_H._kamiyai                .......A......................G.........-------......A......A.........................GA..........
LC008533_Hydatigera_sp._(France)   G.............A...........T...G.........-------......A...G............................GA....A.....
NC061206_Hydatigera_sp._(China)    .G.A..AT.GC..AG.......A..CGA............-------G.....A..C...............................G..........
MW808981_Hydatigera_sp._(China)    .G.A..AT.GC..AG.......A..CGA............-------G.....A..C...............................G..........
NC021141_H._parva                  ...A..ATCGAGAGGG.........TGC..G..G......GAGGTAG.....AT.GA......................G........A......A.....
NC021142_H._kepkogorski            GG.....A..C.AAG.......A.A.....G..C..C..-------......A.G........................G........A......A.....
```

```
                                              6730 tRNA 6740  Trp 6750      6760      6770        6780      6790      6800      6810
                                 ------       --+---------+---------+---------+---------+--------  -+---------+---------+---------+--
ON055368_H._taeniaeformis        TCTTTA-------TAAGATTAAGTTAATATGGACTGTATGTTTTCAAAACATTTAGAGGTTTTA--CCATCTTATGATGATAAGTTTAAATTGACTATTG
FJ597547_H._taeniaeformis        ......-------...................................................--......................C........A
JQ663994_H._kamiyai              ....A.-------.......................A...........................--.................A...............A
NC037071_H._kamiyai              ....G.-------.......................A...........................--.................A...............A
PP104554_H._kamiyai              ....A.-------...............GGA.....A...........................--.................A......C...T.....
LC008533_Hydatigera_sp._(France) ....G.-------.......................G...........................--.................A..........T....A
NC061206_Hydatigera_sp._(China)  ....A.GTGT-TA.............G.G.................................A.AA.............T.............GT..C.A
MW808981_Hydatigera_sp._(China)  ....A.GTGT-TA.............G.G.................................A.AA.............T.............GT..C.A
NC021141_H._parva                ....ATACATACA.............T..--...........................AC...TG-T..........CA..G...A........T.G..A
NC021142_H._kepkogorski          ....GTGAGA--A...AG..........TGT.....A...........................--...CT.......................A....T

                                 cox1 6820      6830      6840      6850      6860      6870      6880      6890      6900      6910
                                 -------+---------+---------+---------+---------+---------+---------+---------+---------+---------+--
ON055368_H._taeniaeformis        AGTTGAATATTTACGTTAGATCATAAGCGTGTTGGTATTATTTATACTTTGTTAGGATTGTGGTCTGGTTTTGTAGGTTTAAGGTTTAGTTTATTAATTC
FJ597547_H._taeniaeformis        .................G............................................A.....................................
JQ663994_H._kamiyai              G.............A....................G..............A..GC....A....................G..T......C.G..G....
NC037071_H._kamiyai              G..................................G..............A..G.....A....................G..T......C.G..G....
PP104554_H._kamiyai              G....G............................................A........A..A.................G...........G.......
LC008533_Hydatigera_sp._(France) GA.................................G.................G.....A..A....................T........G.......
NC061206_Hydatigera_sp._(China)  G....GG.G......C....................G...................T...........G.....G..A.....C................
MW808981_Hydatigera_sp._(China)  G....GG.G......C....................G...................T...........G.....G..A.....C................
NC021141_H._parva                G.....C.T....G.C.T..C...............G.A........A...C.T..C...........A.....G.....G..C..A..G.....G....
NC021142_H._kepkogorski          ......G.......TC.T..........................C...........T........A.................A..............A.

                                 cox1 6920      6930      6940      6950      6960      6970      6980      6990      7000      7010
                                 -------+---------+---------+---------+---------+---------+---------+---------+---------+---------+--
ON055368_H._taeniaeformis        GGGTAAAATTTTTAGAACCTTATTATAAAGTTATACCATTAGATTGTTATAATTTTTTGGTTACTAACCACGGTATAATTATGATTTTTTTCTTTTTGAT
FJ597547_H._taeniaeformis        ...................................................................T..T.............................
JQ663994_H._kamiyai              ....G..T........G....................T..G...........C..............T..T.............................
NC037071_H._kamiyai              ....G..T........G....................T..G...........C..............T..T.............................
PP104554_H._kamiyai              .A..T..C........G....................T..G...........A.................T..........................A..
LC008533_Hydatigera_sp._(France) ....G..T.............................T................................T.............................
NC061206_Hydatigera_sp._(China)  .T..T...........G..C...............T.T.................C..A...........T..G.......................A..
MW808981_Hydatigera_sp._(China)  .T..T...........G..C...............T.T.................C..A...........T..G.......................A..
NC021141_H._parva                ....T..T...........G........C..AG.T..T..............A.....A..A.....T..T..A.....A....................
NC021142_H._kepkogorski          .A.....T........G.................T.................A.....A..A.....T..T..A..T....................A..

                                 cox1 7020      7030      7040      7050      7060      7070      7080      7090      7100      7110
                                 -------+---------+---------+---------+---------+---------+---------+---------+---------+---------+--
ON055368_H._taeniaeformis        GCCAATTTTAATTGGAGGATTTGGTAATTATTTATTGCCGTTATTAGGAGGGTTAGCAGATTTAAATTTGCCTCGATTAAATGCATTAAGAGCTTGATTA
FJ597547_H._taeniaeformis        .......C...............................A.............................A....................T.........
JQ663994_H._kamiyai              ...............T..T..............G.....T...........A..G..C...........A..............T.....T.....G...
NC037071_H._kamiyai              ...............T..T..............G.....T...........A..G..C...........A..............T.....T.....G...
PP104554_H._kamiyai              ...............C..T....................T...........A...........G...C.A..............T..G..T.........
LC008533_Hydatigera_sp._(France) ..................T..............G..A..T...........A..G..T...........A..............T.....T.........
NC061206_Hydatigera_sp._(China)  ...T........A.....T........A...........T........C..A..GT............................G...........G...
MW808981_Hydatigera_sp._(China)  ...T........A.....T........A...........T........C..A..GT............................G...........G...
NC021141_H._parva                ...T........A..T..T.....A..A.....G..A..T.....G..T..T..GT.............A..G..T..............T.........
NC021142_H._kepkogorski          ............A..T..T.....A...........A..T........T..T..GT.T.....G...........G.....C........T.....G...
```

```
                                 cox1 7120      7130      7140      7150      7160      7170      7180      7190      7200      7210
                                 -------+---------+---------+---------+---------+---------+---------+---------+---------+---------+--
ON055368_H._taeniaeformis        TTGGTTCCTTCCTTAGCTTTTTTAGTAGTTAGTATGTATTTAGGAGCGGGTATTGGGTGAACATTTTATCCTCCTTTATCATCTTCTTTATTTTCTGAGA
FJ597547_H._taeniaeformis        ...........A..........................................................................A...........A...G
JQ663994_H._kamiyai              ..A........T..............T..A..A........G..G.............A..G..T..............G.....G...........G..A.
NC037071_H._kamiyai              ..A........T..............T..A..A........G..G.............A..G..T..............G.....G...........G..A.
PP104554_H._kamiyai              C.A........AC.T...........T..A..............G..A.....A..A.....T..................G..C.....G.....A..A.
LC008533_Hydatigera_sp._(France) ..A........T..G.......GA.T..A..............G..T.........A..G..T..............G...........G.....A....
NC061206_Hydatigera_sp._(China)  ..........................T.....A...........T..T...G.A.....G..T...........G..G..T..G.....G..........
MW808981_Hydatigera_sp._(China)  ..........................T.....A...........T..T...G.A.....G..T...........G..G..T..G.....G..........
NC021141_H._parva                .....C.....A..G.TG........GA....G.....C.....T..T..G..A..A..G..T...........A.....T.....A..G......A.A.
NC021142_H._kepkogorski          C.AA.......A..G.........ACTA................T..A..GG.A..T.....T...........A..G..........C.G.....A..A.

                                 cox1 7220      7230      7240      7250      7260      7270      7280      7290      7300      7310
                                 -------+---------+---------+---------+---------+---------+---------+---------+---------+---------+--
ON055368_H._taeniaeformis        GTGTTGGTGTAGATTTTTTAATGTTTTCGTTACATTTGGCTGGTGCCTCTAGGTTATTTGGTTCTATAAAATTTATTTGCACTTTGTATAGAATTTTTAT
FJ597547_H._taeniaeformis        ..............................................T.....CC.........................T....................
JQ663994_H._kamiyai              .A..........................A..G.....A........T........G.....G........T.....C..T..A.........G........
NC037071_H._kamiyai              .A..........................A..G.....A........T........G.....G........T.....C..T..A.........G........
PP104554_H._kamiyai              .A.....C..................C..A.......A..A.....T........G.....A........T........T..A.........G..A.....
LC008533_Hydatigera_sp._(France) .A.....A....................A........A..A.....T.....A........G........T........T..A.....C............
NC061206_Hydatigera_sp._(China)  .G..A.....T...................T..................A..T..G.....G........C........T...........T..A.....
MW808981_Hydatigera_sp._(China)  .G..A.....T...................T..................A..T..G.....G........C........T...........T..A.....
NC021141_H._parva                ..A....G..G........G........T........A.....ATTA......G.T...A.C..A..T...........T.....A....CTG.G.....
NC021142_H._kepkogorski          .A.....A..T.....C...........A........A.....G..T.....T.........................A..T..A..A..............

                                 cox1 7320      7330      7340      7350      7360      7370      7380      7390      7400      7410
                                 -------+---------+---------+---------+---------+---------+---------+---------+---------+---------+--
ON055368_H._taeniaeformis        GACTAATATTTTTTCTCGTACGTCTATTGTACTTTGATCTTATTTGTTTACTTCTATTTTACTACTAATAACTTTGCCAGTTTTAGCAGCTGCTATTACA
FJ597547_H._taeniaeformis        .....................A..........................................T.GG.......A........................T
JQ663994_H._kamiyai              .....................T.....A..T.....G..A.....A..............G...T.G..........A..G........T.....C......
NC037071_H._kamiyai              .....................T.....A..T.....G..A.....A..............G...T.G..........A..G........T.....C......
PP104554_H._kamiyai              .....................T.....A..T.....G..A...C................GT..T..........A..G........T............C
LC008533_Hydatigera_sp._(France) .....................T.....A..T........A....................GT.GT..G.......A..G........T............C
NC061206_Hydatigera_sp._(China)  ......AG.C..........T......A...........A.....................T.GT..........A..T..C..G..G............T
MW808981_Hydatigera_sp._(China)  ......AG.C..........T......A...........A.....................T.GT..........A..T..C..G..G............T
NC021141_H._parva                ......A..A..C........A.....A..GT.G............................T..T..G.......A.....A..G...........A...
NC021142_H._kepkogorski          ......AG.............A.....A..G........C.....A.........A......T.GT..............T.....G...........A..C

                                 cox1 7420      7430      7440      7450      7460      7470      7480      7490      7500      7510
                                 -------+---------+---------+---------+---------+---------+---------+---------+---------+---------+--
ON055368_H._taeniaeformis        ATGTTATTATTTGATCGTAAATTTAGTTCTGCTTTTTTTGATCCTTTAGGTGGTGGTGATCCAGTTTTATTTCAGCATATGTTTTGATTTTTTGGTCATC
FJ597547_H._taeniaeformis        .....GC.............................................................................................
JQ663994_H._kamiyai              .....G........................................................T..A........A.........................
NC037071_H._kamiyai              .....G........................................................T..A........A.........................
PP104554_H._kamiyai              .....G..G...................................G.....G.....C.....T..A......................G........C....
LC008533_Hydatigera_sp._(France) ........G................................C..C.....G...........T..G..G...............................
NC061206_Hydatigera_sp._(China)  .....GC.G..........................C.....C.....G..............T..A........A...........G.............
MW808981_Hydatigera_sp._(China)  .....GC.G..........................C.....C.....G..............T..A........A...........G.............
NC021141_H._parva                .....G..G..........................C........G...........G........G..G.....A..C........G.............
NC021142_H._kepkogorski          .................A..........................A........A..A...........G.....A....................C....
                                                                                                                        JB3 (FORWARD)
```

```
                                  cox1 7520      7530      7540      7550      7560      7570      7580      7590      7600      7610
                                  -------+---------+---------+---------+---------+---------+---------+---------+---------+---------+--
ON055368_H._taeniaeformis         CTGAGGTGTATGTATTGATTCTTCCTGGGTTTGGTATTATTAGACATATATGTTTAAGAATTAGTATGTCTTCGGATGTGTTTGGTTTTTATGGTTTATT
FJ597547_H._taeniaeformis         ................A...........A.......................C...............................................
JQ663994_H._kamiyai               ....A..T.................A..A.......................C.....T..............T..........................
NC037071_H._kamiyai               ....A..T.................A................................T..............T..........................
PP104554_H._kamiyai               ....A..T.............................A....................T..............T..C........C..............
LC008533_Hydatigera_sp._(France)  .A..A..T.....G............................................T..............T.....A..............A..G..
NC061206_Hydatigera_sp._(China)   .G.....T.....T..A...........T.................C........G.................T.....A.................G..
MW808981_Hydatigera_sp._(China)   .G.....T.....T..A...........T.................C........G.................T.....A.................G..
NC021141_H._parva                 .......T.....T..A.................G.....A............................G.GAT....CT..................G..
NC021142_H._kepkogorski           .A.....T........A........A..A........A.....T.................A...........T...........G..............
                                JB3 (FORWARD)
                                  cox1 7620      7630      7640      7650      7660      7670      7680      7690      7700      7710
                                  -------+---------+---------+---------+---------+---------+---------+---------+---------+---------+--
ON055368_H._taeniaeformis         ATTTGCTATGTTTTCTATAGTTTGTTTAGGAAGAAGGGTGTGAGGACATCATATGTTTACTGTTGGGTTAGATGTAAAGACGGCTGTGTTTTTTAGTTCT
FJ597547_H._taeniaeformis         G............................................T..................................................C...
JQ663994_H._kamiyai               ...............A....................A..A..G..T.....C.................G.....T........C..A............
NC037071_H._kamiyai               ...............A....................A..A..G..T.....C.................G.....T........C..A............
PP104554_H._kamiyai               G.....C..............C..C.....T..T..A..A.....T.....C.......................T........................
LC008533_Hydatigera_sp._(France)  G..............A....................A..A..G..T.............................T........A..A........A...
NC061206_Hydatigera_sp._(China)   ...............G..T...........T.....T..A..G..G....................T........T.....T.....T............
MW808981_Hydatigera_sp._(China)   ...............G..T...........T.....T..A..G..G....................T........T.....T.....T............
NC021141_H._parva                 ..................T..G..............T..A.....C.................A..T........G.....T.................A
NC021142_H._kepkogorski           ....................................A........T.....C..............T........T.....T..............C..A

                                  cox1 7720      7730      7740      7750      7760      7770      7780      7790      7800      7810
                                  -------+---------+---------+---------+---------+---------+---------+---------+---------+---------+--
ON055368_H._taeniaeformis         ATAACTATGATTATTGGGGTTCCTACTGGTATAAAGGTTTTTACATGATTGTATATGTTGTTGAATGCTCGAGTCAAAAAGAGTGATCCTGTTTTATGAT
FJ597547_H._taeniaeformis         .................A..A.....A.........................................................................
JQ663994_H._kamiyai               ................................T..........................A..A........T..T......G..........G..G..G.
NC037071_H._kamiyai               ................................T..........................A..A........T..T......G..........G..G..G.
PP104554_H._kamiyai               .................T........A.....T.................A....................T..T......G..........G..G..G.
LC008533_Hydatigera_sp._(France)  ................................T..........................A...........T..T......G..........G..G..G.
NC061206_Hydatigera_sp._(China)   .....G.....A..A.....C..A..A..A..............G.....A..C....................T......G........A.A..G..G.
MW808981_Hydatigera_sp._(China)   .....G.....A..A.....C..A..A..A..............G.....A..C....................T......G........A.A..G..G.
NC021141_H._parva                 G....A........C..............G..............T.....A........AC.T...T.....TA.T..T...G..........AA.T....
NC021142_H._kepkogorski           ..T..............T..G........G..T.................A........A..A...........A..................G..G..G.

                                  cox1 7820      7830      7840      7850      7860      7870      7880      7890      7900      7910
                                  -------+---------+---------+---------+---------+---------+---------+---------+---------+---------+--
ON055368_H._taeniaeformis         GAATTGTTTCTTTTATTATTCTGTTTACGTTTGGCGGAGTTACTGGTATAGTGTTATCAGCTTGTGTTTTAGATAAAGTGTTACATGACACTTGATTTGT
FJ597547_H._taeniaeformis         ..................................T..G..............A.....G.............................T...........
JQ663994_H._kamiyai               ................A..AT.A...........T..G..............A.....T....................A..G.....T...........
NC037071_H._kamiyai               ................A..AT.A...........T..G..............A.....T....................A..G.....T...........
PP104554_H._kamiyai               .....A...........G..T.A.....A.....T.................A.....T........C....................T...........
LC008533_Hydatigera_sp._(France)  .....A..........AG.GT.A...........T..G..............A..G..C.............................T...........
NC061206_Hydatigera_sp._(China)   ................AG.G..A.....A.....G.....A...........T..G..T........A............C.G.....T..G........
MW808981_Hydatigera_sp._(China)   ................AG.G..A.....A.....G.....A...........T..G..T........A............C.G.....T..G........
NC021141_H._parva                 .............C..AG..T.A...........T..T..C...........T..........................T..G.....T...........
NC021142_H._kepkogorski           ..........A.....A..AT.......A.....T.................A..G..T.............................T.....G.....
```

```
                                  cox1 7920      7930      7940      7950      7960      7970      7980      7990      8000      8010
                                  -------+---------+---------+---------+---------+---------+---------+---------+---------+---------+--
ON055368_H._taeniaeformis         AGTAGCTCATTTTCATTATGTTATGTCGCTTGGTTCTTATATAAGAATAATAATAATGTTTATTTGATGATGACCATTAATAACTGGTTTGAGGTTGAAT
FJ597547_H._taeniaeformis         .................................C........................................................A.........
JQ663994_H._kamiyai               G..G..G..................T........G........G..T..................G.....G............A.......T..A...
NC037071_H._kamiyai               G..G..G..................T........G........G..T..................G.....G............A..A..T..A...
PP104554_H._kamiyai               ...........................TT.A.....A...........T....................G..............C.....A..A..A...
LC008533_Hydatigera_sp._(France)  T..G.......................T........A...........T....................G..G..............G..A..A..A...
NC061206_Hydatigera_sp._(China)   G..G.......................TT.A..G.....C........T..............A.....G..G........T......C....T......
MW808981_Hydatigera_sp._(China)   G..G.......................TT.A..G.....C........T..............A.....G..G........T......C....T......
NC021141_H._parva                 T..T..............C.........T.A.....A.....T..T..T..T.......................T.....T.....A..A.....A..A
NC021142_H._kepkogorski           T..........................AT.A........C........T..............A..G..G..G..T........A.....A..T..A...
                                                 JB45 (REVERSE)
                                  cox1 8020      8030      8040      8050      8060      8070      8080      8090      8100      8110
                                  -------+---------+---------+---------+---------+---------+---------+---------+---------+---------+--
ON055368_H._taeniaeformis         AAGTGTTTGTTGCAATGTCAGTGTATAGTTTCTCAAATTGGGTTTAATTTGTGTTTTTTTCCTATGCATTATTTTGGATTGTGTGGATTACCACGTCGTG
FJ597547_H._taeniaeformis         ........A.....G.................................................................A.....G.............
JQ663994_H._kamiyai               ...........A.......................G..A..A........A...................................G.............
NC037071_H._kamiyai               ...........A.......................G..A..A........A...................................G.............
PP104554_H._kamiyai               ........A.............................A....................C..A.................A.....G.............
LC008533_Hydatigera_sp._(France)  ........A..A..G....................G..A..A...........................................G.............
NC061206_Hydatigera_sp._(China)   ..............G..............G........C..T........A..C........................C.T.....C...........G.
MW808981_Hydatigera_sp._(China)   ..............G..............G........C..T........A..C........................C.T.....C...........G.
NC021141_H._parva                 ........A.....G...........TA.......GG.............A........C..A..............T........G.............
NC021142_H._kepkogorski           ........A..A........A......A..........A..A.....C..A..........................G..A.....G.............

                                  cox1 8120      8130      8140      8150      8160      8170      8180      8190      8200      8210
                                  -------+---------+---------+---------+---------+---------+---------+---------+---------+---------+--
ON055368_H._taeniaeformis         TTTGTATTTATGAATGTGCTTATAATTGAATAAATATAGTATGTACTGTTGGATCTTTTATATCGGCTTTTAGAGGATGTTTTTTTGTATTTATTTTATG
FJ597547_H._taeniaeformis         ............................................................................G.......................
JQ663994_H._kamiyai               ...............................T....................G..A........A........T.....C........T...........
NC037071_H._kamiyai               ...............................T....................G..A........A........T.....C........T...........
PP104554_H._kamiyai               ...............................T................................A........T.....C....................
LC008533_Hydatigera_sp._(France)  ...............................T........G...........G...........A........T..G..................C.G..
NC061206_Hydatigera_sp._(China)   .......G.............................T..G..C........T........T..T..C.....G..G...........G........G..
MW808981_Hydatigera_sp._(China)   .......G.............................T..G..C........T........T..T..C.....G..G...........G........G..
NC021141_H._parva                 .....G.G.....G.....A.....A...........T..T......A....G.........A.T........T.CT...........C........G..
NC021142_H._kepkogorski           .....G.............A...........T......A.G.....A.....T..A.................T..T....................G..

                                  cox1 8220      8230      8240      8250      8260      8270      8280      8290      8300      8310
                                  -------+---------+---------+---------+---------+---------+---------+---------+---------+---------+--
ON055368_H._taeniaeformis         AGAATCAATAGTGAGTTCTAATTATGTTTTAGGTTCATACGGTATTTCTGGGTGTATGGCTGATTTTTTTGTGAGACCAATGGCTTGTCATAAAGATTAT
FJ597547_H._taeniaeformis         G......................................T............................................................
JQ663994_H._kamiyai               ...G..T.....T..............A........T..T...........T........C...........T..............C............
NC037071_H._kamiyai               ...G..T.....T..............A........T..T...........T........C...........T..............C............
PP104554_H._kamiyai               ......T.....T.....C........AC.......T..T........A..C....................T.....G...........C.........
LC008533_Hydatigera_sp._(France)  ......T.....T..............G..G........T...........T....................T.....G........C............
NC061206_Hydatigera_sp._(China)   ...G..C.....C.....A..C...........G..T..T..GG.......T...........................T.A..C.....C.........
MW808981_Hydatigera_sp._(China)   ...G..C.....C.....A..C...........G..T..T..GG.......T...........................T.A..C.....C.........
NC021141_H._parva                 ......T..TA.T..AAA....A................T.....G.........G.T.T....C.....T.T.CT..T.....AA.......T..A...
NC021142_H._kepkogorski           ......G.....T..A..A...........G..A..G..T..A........T......A.............A..T..G......A...............
```

```
                                 cox1 8320      8330      8340      8350      8360      8370      8380      8390      8400      8410
                                 -------+---------+---------+---------+---------+---------+---------+---------+---------+---------+--
ON055368_H._taeniaeformis        TATTGTTATCCTTATAGAGTTGATTATACATATGGTGTTTATTATATGCGTTGAATTGATGATTGTACATATGTTTTTGCTCGTGGGTGTGCTGATGGTT
FJ597547_H._taeniaeformis        ..........................C................................................................T........
JQ663994_H._kamiyai              .................T........C..G........................G...................................AT......C.
NC037071_H._kamiyai              ........C........T........C..G........................G...................................AT......C.
PP104554_H._kamiyai              ..........................C...........G.........................................G..........T......C.
LC008533_Hydatigera_sp._(France) ..............C..G...........T........G...............G....................................T......C.
NC061206_Hydatigera_sp._(China)  ...........C.................T.T....AC...............GG.A.......................G....CT....T.A--..C.
MW808981_Hydatigera_sp._(China)  ...........C.................T.T....AC...............GG.A.......................G....CT....T.A--..C.
NC021141_H._parva                ...........G.....T...........T.......................G..A...........T.....G.....A..-...G--A..A------
NC021142_H._kepkogorski          ........C........T...........T.....C.................GG................C........G..........TA.---.C.

                                      8420      8430 tRNA 8440  Thr 8450      8460      8470           8480      8490      8500 16S-rRNA
                                 -------+---------+---------+---------+---------+---------+-  --------+---------+- --------+---------
ON055368_H._taeniaeformis        ATTTAGTTTAATAAAAATATGGGTTTTGTAGTCCTAAGATGATTTTAATCATTAACCTA--ATTTTAGTAGATACTAATAT-ATGTGTTTAGGTTTATTT
FJ597547_H._taeniaeformis        ...........................................................--.........A.....T....-..................
JQ663994_H._kamiyai              ............G.........................................G....---......A.A...T...G..-....A.............
NC037071_H._kamiyai              ............G.........................................G....---......A.A...T...G..-....A.............
PP104554_H._kamiyai              G...........T.........................................G....---........A...T.T.A..-....A..C......G...
LC008533_Hydatigera_sp._(France) G.....................................................G....---.....A..A..GTC..G..-..A.A........A....
NC061206_Hydatigera_sp._(China)  T..........A......G.......................A..AT......GG....--.........A...T.TGA..-GCT.A.............
MW808981_Hydatigera_sp._(China)  T..........A......G.......................A..AT......GG....--.........A...T.TGA..-GCT.A.............
NC021141_H._parva                -.........-.......G...........TA..C.......G..-.T...G..GT..TAT.C....AA.AG.GT.T.AT.TGA..A...T....A....
NC021142_H._kepkogorski          G.................G...........A.......................G....--......A..A...TA..GG.-GAT.A.............

                                8510      8520      8530      8540      8550      8560      8570      8580      8590      8600 16S-rRNA
                                 +---------+---------+---------+---------+---------+---------+--------- +---------+---------+-  -----
ON055368_H._taeniaeformis        GCCTTTTGCATCATGCTTATTGGATTATTTTAAAATATTTTGTTAACCAAAAAATCTAGATCTTATAATT-ATTTGTTTTGAACTTAAAAATC--TTAAG
FJ597547_H._taeniaeformis        ..........................G...........................................-......................--.....
JQ663994_H._kamiyai              ..........................GAA.....G......A...........G............G...-G..............TT.T-.T--..G..
NC037071_H._kamiyai              ..........................GAA.....G......A.C.........G............G...-G..............TT.T-.T--..G..
PP104554_H._kamiyai              ...........................A..G...G......AA...........................TGCCC...A.......T.GT-.T--.....
LC008533_Hydatigera_sp._(France) ...........................A......G......A...........G............G...-G..............TT.T-.T--..GG.
NC061206_Hydatigera_sp._(China)  ...........................G......G......T.A....G...G...............C.-GC............GTT.T-.A--..G..
MW808981_Hydatigera_sp._(China)  ...........................G......G......T.A....G...G...............C.-GC............GTT.T-.A--..G..
NC021141_H._parva                ..................TGA......GAA-.GTTA...C..GA....G....G.T........GA....-...A...........TG.T..TAA..T..
NC021142_H._kepkogorski          ..........................GA.A....T......AACT...G............T..G.G...-.C....A......ATTTT-.T--...G.

                                   8610      8620      8630      8640      8650      8660      8670      8680      8690      8700 16S-rRNA
                                 ---+ ---------+---------+---------+---------+---------+---------+---------+---------+---------+-----
ON055368_H._taeniaeformis        TAAA-GAATAATGAAAATAATTGTATGGTGTGATAAATTATTCGTTAGATTTTATATTTGTTTTACTGCTTTCTATTTAATAATATGTAATTGGCTATTA
FJ597547_H._taeniaeformis        ....-.................T.............................................................................
JQ663994_H._kamiyai              .T..-A......T.G...G..CA.................................................G.T....G.GT.....TG..........
NC037071_H._kamiyai              .T..-A......T.G...G..CA.................................................G.T....G..T.....TG..........
PP104554_H._kamiyai              .T..-A........G.GCG..............C..G...C...............................G.T...CG.G.C...CT...........
LC008533_Hydatigera_sp._(France) ....-.......T....C...C..................................................G......G........T...........
NC061206_Hydatigera_sp._(China)  .T..TA.G..GAT.G.G..G..T....T........G...........G.......GC......G......GAAG....G..........G.........
MW808981_Hydatigera_sp._(China)  .T..TA.G..GAT.G.G..G..T....T........G...........G.......GC......G......GAAG....G..........G.........
NC021141_H._parva                GT..-AGT..GGA......G..T....T.....A......G......A.........C....AA.......AA..-..GTA.T...A...A.A.T...A.
NC021142_H._kepkogorski          .T..-AG..G.AA.G.G.....A..G.A........G...................AC......GT......GA.A...GAGT...A...........G.
```

```
                                    8710      8720      8730      8740      8750      8760      8770      8780      8790        8800 16S-rRNA
                                 ----+---------+---------+---------+---------+---------+---------+---------+---------+ --------+-----
ON055368_H._taeniaeformis        GGTTAATTATAAGTTTATATTATTTTTTGTGTTGTGTTTTTATTGGGTTAAAAGTACCCATTATTTGTAAAGTTGTTATAAACTT-GAATTTGTGTTAAT
FJ597547_H._taeniaeformis        ............................C........................................................-.G............
JQ663994_H._kamiyai              ...C.G..GC.G..C......G.....AT.A.C..T..........................T.......G..............-AGT...A..A....
NC037071_H._kamiyai              ...C.G..GC.G..C......G.....AT.A.C..T....-.....................T.......G..............-AGT...A..A....
PP104554_H._kamiyai              ...C.G..GC.G.........GC....GT.A....T..........................T...A...G..............-AG....A.AA....
LC008533_Hydatigera_sp._(France) ...C.G..GC.G.........G.....AT.A....T..........................T......................-AGT.....AA....
NC061206_Hydatigera_sp._(China)  .....G...C.T.......C.....A...G..CTCA..AA..........G.........A.T.......G..............-AG.AA.T..AGGG.
MW808981_Hydatigera_sp._(China)  .....G...C.T.......C.....A...G..CTCA..AA..........G.........A.T.......G..............-AG.AA.T..AGGG.
NC021141_H._parva                .A...T.......G.........GAG...G...TCA.GAAA...A.......G.....TG..T...AA..G...........G..-A.GAA.T..G.GGA
NC021142_H._kepkogorski          A....G....T.AA..T....G.....AA.T.AA.T...A............G.......A.T...A...G..............TATGAAC...A.G..

                        16S-rRNA 8810      8820      8830      8840      8850      8860      8870      8880      8890      8900
                                 --  ---+----- ----+---------+---------+---------+---------+---------+---------+---------+---------+-
ON055368_H._taeniaeformis        TT--AATTAAGTG-TTTAGCAGTATATAAATAAAATTTAGATGATAATAATCAATAAATAAGTAATTCAATTATATTAATTATTTCTAGTAACTTTTCCG
FJ597547_H._taeniaeformis        ..--........A-..........................G..G....................C.................................
JQ663994_H._kamiyai              G.TA.....G..AA.A.G.......TG.T-....GG..G...ATC.G.G.................T......G........................
NC037071_H._kamiyai              G.TA.....G..AA...G.......TG.T-....GG......ATC.G.G.................T......G........................
PP104554_H._kamiyai              A.AA.....GA.AA...G.......T...-............AT.G.........G..........T......G.............A..G........
LC008533_Hydatigera_sp._(France) A.TA..........AG...G..........-....GA......AT........................T......G........................
NC061206_Hydatigera_sp._(China)  GAATGG....A.TTA..G.T..C..TCGTT...TG.....TGAT......................T.......C.............A.GG.......
MW808981_Hydatigera_sp._(China)  GAATGG....A.TTA..G.T..C..TCGTT...TG.....TGAT......................T.......C.............A.GG.......
NC021141_H._parva                GA--......T.TTA.AG.....-..A.TG...T.G....TA.T.T......TCAT........G..TT......C............AGGGTC......
NC021142_H._kepkogorski          GGC-...A.....TG..G...AC..TAG.-...T....G.TATTC.....................T..G................CAGGG.........

                        16S-rRNA 8910      8920      8930      8940      8950      8960      8970      8980      8990      9000
                                 --------+---------+---------+---------+---------+---------+---------+---------+---------+---------+-
ON055368_H._taeniaeformis        TCTGTTTATTAAAAACATTGCCAATTTAAAATTTAGTTGGTAGTACCTGCCCAGTGTTGTTAATAAATGGCCGCAGTATATTGACTGTGCAAAGGTAGCA
FJ597547_H._taeniaeformis        ..............................T.....................................................................
JQ663994_H._kamiyai              .....................T........T....A..A......................T.....C................................
NC037071_H._kamiyai              .....................T........T....A..A......................T.....C................................
PP104554_H._kamiyai              .....................T.............A........................-.......................................
LC008533_Hydatigera_sp._(France) .....................T.............A..A.............................................................
NC061206_Hydatigera_sp._(China)  ...................T.T........TA...A..A..............A.....C....T...................................
MW808981_Hydatigera_sp._(China)  ...................T.T........TA...A..A..............A.....C....T...................................
NC021141_H._parva                ...................T.T..C..T..GAA...........G.....T.....C.T.-GG.....A...............................
NC021142_H._kepkogorski          ...................T.T.....T...C...A..A..............A.......T......................................

                        16S-rRNA 9010      9020      9030      9040      9050      9060      9070      9080      9090      9100
                                 --------+---------+---------+---------+---------+---------+---------+---------+---------+---------+-
ON055368_H._taeniaeformis        TAATTAATTGCCTTTTAATTGGGGGCTTGTTTGAATGGTTTGACGAGATAAGTTTTAATATTTAATATTTGAATGAAATTAATGTTAAGGGTTCAGAATC
FJ597547_H._taeniaeformis        .......................................................................TG...........................
JQ663994_H._kamiyai              ............C........................................G...........C.....TG....T..G..A................
NC037071_H._kamiyai              ............C........................................G...........C.....TG....T..G..A................
PP104554_H._kamiyai              ....................................................CG................ATG...........................
LC008533_Hydatigera_sp._(France) .....................................................G...........C.....TG....T.....A................
NC061206_Hydatigera_sp._(China)  ............C......................................A.A.G........G.....TGT....T..G..A..G.............
MW808981_Hydatigera_sp._(China)  ............C......................................A.A.G........G.....TGT....T..G..A..G.............
NC021141_H._parva                ..........T.............A..........A.........T....GA..A.........G...A.A.T.......G..AA.GTT...........
NC021142_H._kepkogorski          ................................................A...AGCG..............AGT....T.....A................
```

```
                                  16S-rRNA 9110      9120      9130      9140      9150      9160      9170      9180      9190      9200
ON055368_H._taeniaeformis         CTTATATATTAATAAGACGGAAAGACCCTAGGATCTTTTGTTTTTTGTTTGGGGCAAATTTTTATAGTTTATAAAGATTGTGACCCTAA-TGGGTTATTG
FJ597547_H._taeniaeformis         .............................................................G...........G.A.............-..........
JQ663994_H._kamiyai               .......................................A.....-...............G....A......G.A.............-..........
NC037071_H._kamiyai               .......................................A.....-...............G....A......G.A.............-..........
PP104554_H._kamiyai               .....G.................................A.....-...............G....A......G.A.............-..........
LC008533_Hydatigera_sp._(France)  .......................................A.....-...............G...........G.A....C........-.......A..
NC061206_Hydatigera_sp._(China)   ....ATAG...............................A.....-...............A.G.TT.G..C.GTA...A.........A...T......
MW808981_Hydatigera_sp._(China)   ....ATAG...............................A.....-...............A.G.TT.G..C.GTA...A.........A...T......
NC021141_H._parva                 .GC..GAT.....................G........AAG....-.C.........G....AT.GT....AT..A....A......TGT...T...A..
NC021142_H._kepkogorski           ....AGAT..............................CA.....-...................GA........T...TA......T.-...T...G..

                                  16S-rRNA 9210      9220      9230      9240      9250      9260      9270      9280      9290      9300
ON055368_H._taeniaeformis         GGTTAAGTTACCCTAGGGATAACAGAGTAATAATTAATGAGAGATCATATCGAATTAATTGTTTGCTACCTCGATGTTGACTTAGGTTTAAGACTTAGGT
FJ597547_H._taeniaeformis         ..............................................................................................G.......
JQ663994_H._kamiyai               ...A..........................................T.........................................-.GAG.......
NC037071_H._kamiyai               ...A..........................................T.........................................-.GAG.......
PP104554_H._kamiyai               A..A..........................................T.........................................-.G.G.......
LC008533_Hydatigera_sp._(France)  A..A........................G.................T.........................................-.G.G.......
NC061206_Hydatigera_sp._(China)   AA.A........................G...G.............T.........................................-TC.G.......
MW808981_Hydatigera_sp._(China)   AA.A........................G...G.............T.........................................-TC.G.......
NC021141_H._parva                 A............C........................A.......T......G..................................--.TG.......
NC021142_H._kepkogorski           A..A.....................T..G...G.............T...................C.....................-.GAGT......

                                  16S-rRNA 9310      9320      9330      9340      9350      9360      9370      9380      9390
ON055368_H._taeniaeformis         GTAGTAGTTTAAGTCGTTGGTCTGTTCGACCTTAAAA-CCTCCATGAGTTGAGTTAAGACCGGCGTGAGCCAGGTCGGTTCTTATCTATTGTAGAAATTT
FJ597547_H._taeniaeformis         ..............T......................A..............................................................
JQ663994_H._kamiyai               ..............T..A...............T...-..............................................................
NC037071_H._kamiyai               ..............T..A...............T...-..............................................................
PP104554_H._kamiyai               .............CT..A...................-....................................................A.........
LC008533_Hydatigera_sp._(France)  .C............T..A...............T...-..............................................................
NC061206_Hydatigera_sp._(China)   .C........G...T..G...................-...T..........................................................
MW808981_Hydatigera_sp._(China)   .C........G...T..G...................-...T..........................................................
NC021141_H._parva                 .C......C..G.CTT.A..............AG...-..................T.......................................G.....
NC021142_H._kepkogorski           ....CC......ACT..A...................-......................................................A........

                                  9400      9410      9420      9430      9440 tRNA 9450 Cys 9460      9470      9480      9490 12S-rRNA
ON055368_H._taeniaeformis         ATCAGTACGAAAGGATAGTAAATTTTTTTATTGAGTATGAAAT-AAAATTAGTTTTGCAAAAACTAATTAGAATTAGGTTAATTCCATACTTTAATTGTT
FJ597547_H._taeniaeformis         ........................................T..-......G.................................................
JQ663994_H._kamiyai               ...........................................G.....C...............G.........GA.......................
NC037071_H._kamiyai               ...........................................G.....C...............G.........GA.......................
PP104554_H._kamiyai               .........................................----....................G.........GAA...............G......
LC008533_Hydatigera_sp._(France)  ................................A......T.C.-...G.................G..........AA..........G.....G.....
NC061206_Hydatigera_sp._(China)   .....................G..........A...G...T.--...G............................T.T.G........G....GG.....
MW808981_Hydatigera_sp._(China)   .....................G..........A...G...T.--...G............................T.T.G........G....GG.....
NC021141_H._parva                 ...............C........................GT.--GTT...................A.T....ATTT.AT.............T.....
NC021142_H._kepkogorski           ....................................G.A...--G....................G..........A-.A.....T.C............
```

```
                                  9500      9510      9520      9530      9540      9550       9560      9570      9580 12S-rRNA 9590
                                  -+---------+---------+---------+---------+---------+----- ----+---------+---------+--     -------+--
ON055368_H._taeniaeformis         TAATTGTGGCAATAGAAAGTTTATTCTTTATTATCTGCATAAATAGTTATTAGATGT-TATGATGAAATTATTAAGTTTTGATTT-----ATTAGCAGTT
FJ597547_H._taeniaeformis         .......................................................A.-........G...G.............C-----..........
JQ663994_H._kamiyai               .....A..................C....................AA.T..G.....-.G..T.A.G...G.........TG.A------..........
NC037071_H._kamiyai               .....A..................C....................AA.T..G.....-.G..T.A.G...G.........TG.A------..........
PP104554_H._kamiyai               .....A.......................................AA.T....G...A.G..T.A..........A....TG.A------..........
LC008533_Hydatigera_sp._(France)  .....A..................C....................AA.T..T.G...-.G.CT.A.T...G.........T..A------..........
NC061206_Hydatigera_sp._(China)   .....A.......GA.......G.CT...G...............T...C.TTG-..A.T.A.AT.....G..........G.G------..........
MW808981_Hydatigera_sp._(China)   .....A.......GA.......G.CT...G...............T...C.TTG-..A.T.A.AT.....G..........G.G------..........
NC021141_H._parva                 .....A...............-.G.T.C....TA.........ACTA..A.TT..TGAG..AGAT.TTAGC...GA...GAT.AAGTTTA..........
NC021142_H._kepkogorski           .....A...............-.....................ATTA..A..TG-A.TCGATG...C.A.G........CAT.A------..........

                         12S-rRNA 9600      9610      9620      9630      9640      9650      9660      9670      9680      9690
                                  -------+---------+---------+---------+---------+---------+---------+---------+---------+---------+--
ON055368_H._taeniaeformis         AGTTTTTTGTCAAAATTTGTGTTTTAAAAGAATAAAGAATATAAAGGGGATAGGACACAGTGCCAGCATCTGCGGTTAATCTGTTTTCTTTGCTTTTAAA
FJ597547_H._taeniaeformis         ..........T.........................................................................................
JQ663994_H._kamiyai               ..........T..........................GG...G...................................G..................T.G
NC037071_H._kamiyai               ..........T..........................GG...G...................................G..................T.G
PP104554_H._kamiyai               ..........T........C......T..........GG..........................................................T.G
LC008533_Hydatigera_sp._(France)  ..........T........C........G........GG...G...................................G..................T.G
NC061206_Hydatigera_sp._(China)   ..........T......G........G........GAGGCGA............G...............C...........................TG
MW808981_Hydatigera_sp._(China)   ..........T......G........G........GAGGCGA............G...............C...........................TG
NC021141_H._parva                 .T........T.....CAA.......T.......G.AGC..A............T.......................T.................CT.G
NC021142_H._kepkogorski           .A........T........C......G........G.....A......................................................CT.C

                          12S-rRNA 9700      9710      9720      9730      9740      9750      9760      9770      9780
                                  ----- --+ ---------+---------+---------+---------+---- -----+---------+---------+---------+---------
ON055368_H._taeniaeformis         TAGAG-GTG-ATTAAATTAATAATAAGTAAAAGTGGGTTAAATTTGTTTATTGT-GTTTTTAAGTGATTTATTTTACATAAAATAGGTTTTATGACAGGG
FJ597547_H._taeniaeformis         .....-...-......................A.....................-.................C...T.......................
JQ663994_H._kamiyai               .....-...-G........T............T...................AA-A.........A.......C..A.....G.G.A.............
NC037071_H._kamiyai               .....-...-G........T............T...................AA-A.........A.......C..A.....G.G.A.............
PP104554_H._kamiyai               .....-..A-...T.....T...C..A.....T...................AA-.......TA.A..........T.....G.G...............
LC008533_Hydatigera_sp._(France)  .....-..A-.........T...C........T...................TA-.A.....TA.A..........A.....G.G...............
NC061206_Hydatigera_sp._(China)   ....A-A.T-G........G..C....C....A..........C.....G...C-AG....GGA.A.....C...TT.....G.G.T..AA.........
MW808981_Hydatigera_sp._(China)   ....A-A.T-G........G..C....C....A..........C.....G...C-AG....GGA.A.....C...TT.....G.G.T..AA.........
NC021141_H._parva                 ....ATA.AT...T.....T...CTT.A....T.A..........T...T.G..T.A.G..G.A.AG..A.G....AGG...G.TT.G............
NC021142_H._kepkogorski           ....A-A.A-.G.......T.....G......T.A.................A.-.A.A...T............TGGAG..G...A.ACA.........

                                9790      9800      9810      9820      9830      9840      9850      9860      9870      9880 12S-rRNA
                                  +---------+---------+---------+---------+---------+---------+---------+---------+---------+---------
ON055368_H._taeniaeformis         ATTAGATACCCCATTAATACTATTGGTAATATATCTTAGTTTTGTAACTAAAATAATTTGGCAGTGAGCGATTCTTATTAGGGGAAGGTGTGGTGTAAAG
FJ597547_H._taeniaeformis         ...........................................A........................................................
JQ663994_H._kamiyai               ........................AA......G..........A................................T.......................
NC037071_H._kamiyai               ........................AA......G..........A................................T.......................
PP104554_H._kamiyai               ....................C...AA......G..........A................................T.......................
LC008533_Hydatigera_sp._(France)  ........................AA......G..........A................................T.......................
NC061206_Hydatigera_sp._(China)   ....................CT..A.....G............A........................T.......T.........A.....A.......
MW808981_Hydatigera_sp._(China)   ....................CT..A.....G............A........................T.......T.........A.....A.......
NC021141_H._parva                 ...................T.T.CTTA.T.TG.........AAA........................T......................AA.A.....
NC021142_H._kepkogorski           ........................A..................A........................T...............................
```

```
                                 9890      9900      9910      9920      9930      9940      9950      9960      9970      9980 12S-rRNA
ON055368_H._taeniaeformis        GATGGTCCGCCTAATAATTTACTTTTATTATATTGGTGTATATCTGGTTTAATATTATTGTTGAGTAA-CAGAAGTTGTGTAATATTATTTAAGCCAAGT
FJ597547_H._taeniaeformis        ...................C................................................-...............................
JQ663994_H._kamiyai              ....A.......................................................C...A.GG-.............G.G...............
NC037071_H._kamiyai              ....A.......................................................C...A.GG-.............G.G..........T....
PP104554_H._kamiyai              .............................................................CA.A.G.-.............G...A.............
LC008533_Hydatigera_sp._(France) ............................................................C...A.GG-.............G.................
NC061206_Hydatigera_sp._(China)  ..........................G.......................T.........C.T.A.GG-.............T...A.............
MW808981_Hydatigera_sp._(China)  ..........................G.......................T.........C.T.A.GG-.............T...A.............
NC021141_H._parva                ....A..........................G..........................C...T.....GT.T.G.........GT...............
NC021142_H._kepkogorski          ..C.A.............................................................G.-.............G...A.............

                                 9990      10000     10010     10020     10030     10040     10050     10060     10070     10080 12S-rRNA
ON055368_H._taeniaeformis        CTATGTGCTGTTTATAAAAGTATTCATGCGTTACATTAATAAATGTTAGATTGTGATATTTGATAATTTAGGACTTAAAAGTAATGTTTAATAAGTTAGT
FJ597547_H._taeniaeformis        ....................................................................................................
JQ663994_H._kamiyai              ..........................................G.A..TA.....A.....A...G...........................T.A.....
NC037071_H._kamiyai              ..........................................G.A..TA.....A.....A...G...........................T.A.....
PP104554_H._kamiyai              ...............GG..............................TA.....A.....A...T...........................C.......
LC008533_Hydatigera_sp._(France) ............................................A..TA.....A.....G........................A......T.A..G..
NC061206_Hydatigera_sp._(China)  ...........A....G....................T......A..TA.....A.G...AA..T...................................
MW808981_Hydatigera_sp._(China)  ...........A....G....................T......A..TA.....A.G...AA..T...................................
NC021141_H._parva                ................G.......T............G....GGTGCCTGG...A....AG.T...............T......A..A........T..
NC021142_H._kepkogorski          .....................................T......A..TA.....A.....GA.A.....................A........A.....

                                 10090     10100     10110     10120     10130     10140       10150     10160     10170     10180 12S-rRNA
ON055368_H._taeniaeformis        AAACGTGAAAAGAGTTTAGCTCAGGTACACACCGCCCGTCACCCTCGATGAT--TATTGAGGTAAGTCGTAACAAGGTAACTTTAAATGAATTTGAAGTT
FJ597547_H._taeniaeformis        ....................................................--..............................................
JQ663994_H._kamiyai              ...T......TA.....................................T..--..............................................
NC037071_H._kamiyai              ...T......TA.....................................T..--..............................................
PP104554_H._kamiyai              ...T......TA.....................................T..--...............................G..............
LC008533_Hydatigera_sp._(France) ...T......TA.....................................TTA--..............................................
NC061206_Hydatigera_sp._(China)  .G.......G.A.....................................T..GT.G.......................G....................
MW808981_Hydatigera_sp._(China)  .G.......G.A.....................................T..GT.G.......................G....................
NC021141_H._parva                TG.TA....GTA...........T.........................T..--..............................................
NC021142_H._kepkogorski          G..T......TA...........T.........................T..--..............................................

                                   10190 12S-Rrna 10200     10210     10220     10230     10240     10250     10260     10270 cox2 10280
ON055368_H._taeniaeformis        AGTTACTGGTTA----ATAAATTTAATCAGTTAGAATGAAACTATCATTGTTGTATTATGATATTGTATGTTATATAATAGCTGTGTGTATGTTTATAAT
FJ597547_H._taeniaeformis        ............----.................A..................................................................
JQ663994_H._kamiyai              G...........----................GA.......T..........A...........A...................................
NC037071_H._kamiyai              G...........----................GA.......T......................A...................................
PP104554_H._kamiyai              G..........G----.....A...........A.......T....T..A..A.........................................C.....
LC008533_Hydatigera_sp._(France) G...........----...G........G....A.................................G................................
NC061206_Hydatigera_sp._(China)  ..........----------G...........GA.......T.......A..A..............G..C...................C...C..TT.
MW808981_Hydatigera_sp._(China)  ..........----------G...........GA.......T.......A..A..............G..C...................C...C..TT.
NC021141_H._parva                .........A.---------T.AGT.C.....GT.......T.G..T..A........C.....A..T..C.....T........A...G.A......T.
NC021142_H._kepkogorski          .......A.AAGTAATG..G.G.CT.CT....GA.......T....G.................A..G.................T...........TC.
```

```
                                 cox2 10290          10300          10310          10320          10330          10340          10350          10360          10370          10380
                                 -------+---------+---------+---------+---------+---------+---------+---------+---------+---------+--
ON055368_H._taeniaeformis        TTGTTTTGTTTATATAATGCTAGGTTGAAATTTGTTTATGAGTAAGGGTAGTGTTAAATTTGGTGGAGAAAATCAATTTGTTGAGCTTTCATGAACAATT
FJ597547_H._taeniaeformis        ...........................G........................................................................
JQ663994_H._kamiyai              .............T.T...T.G.....G....................C..C.......................G........................
NC037071_H._kamiyai              .............T.T...T.G.....G....................C..C.......................G........................
PP104554_H._kamiyai              ...............T...T.G...........A........G.....................................................G...
LC008533_Hydatigera_sp._(France) .............T.T...T.G....................C................................G........A...............
NC061206_Hydatigera_sp._(China)  G..............T...T..A...................C................................GG.......AT.G.....G..G...
MW808981_Hydatigera_sp._(China)  G..............T...T..A...................C................................GG.......AT.G.....G..G...
NC021141_H._parva                A..................T..TT...G.....A.......A..GT.....G........................AC.........G.T......GG.A
NC021142_H._kepkogorski          ...............T...T.GA...................G.....A.....G........A..G..G......G....G..............T...

                                 cox2 10390          10400          10410          10420          10430          10440          10450          10460          10470          10480
                                 -------+---------+---------+---------+---------+---------+---------+---------+---------+---------+--
ON055368_H._taeniaeformis        ATACCTACAATGGTTGTTTTAGTCTTATGTGCTTTAAATGTTAAATTTATAACTAGTGATTTGGATTGTTTTTCTAGTGAAACTATTAAGATTATAGGTC
FJ597547_H._taeniaeformis        G.............C........T..G.....C..G..........................A..............A......................
JQ663994_H._kamiyai              G.G....................T..G........G................................................................
NC037071_H._kamiyai              G.G....................T..G........G................................................................
PP104554_H._kamiyai              G....A..T..............T..G........G.....A..T........G........A....................A................
LC008533_Hydatigera_sp._(France) G.G...........C........T..G...................................A....................A..............G.
NC061206_Hydatigera_sp._(China)  ..C.....T........GC....T..G..............A..T........C........A..C..............G..............T..A.
MW808981_Hydatigera_sp._(China)  ..C.....T........GC....T..G..............A..T........C........A..C..............G..............T..A.
NC021141_H._parva                G.T.....T.....A..G...A.A......T...............................A...........A..C..G...........AG.T....
NC021142_H._kepkogorski          G......................T.................A..T........G........A.................G..A........A..T..C.

                                 cox2 10490          10500          10510          10520          10530          10540          10550          10560          10570          10580
                                 -------+---------+---------+---------+---------+---------+---------+---------+---------+---------+--
ON055368_H._taeniaeformis        ATCAGTGATATTGGACTTATGAATACCCGGATGGTTATTATGATTCTTTTATGACTAAGGATTGTTTTCTGGTTGATAAGCCATTGCGTATGTTTTATGG
FJ597547_H._taeniaeformis        .........................T..A...........C...........................T...............................
JQ663994_H._kamiyai              ..........C.....A........T........G.................A...............T.............T.................
NC037071_H._kamiyai              ..........C.....A........T........G.................A...............T.............T.................
PP104554_H._kamiyai              .............A..A........T..A.....C.................T...............T.A..G........T..............C..
LC008533_Hydatigera_sp._(France) .............A..G.....G..T..A.....A..C..............................T.A...........T..A..............
NC061206_Hydatigera_sp._(China)  ....A.....C..A........G..T..A.........................CT............T.A..A..C.....T..A..............
MW808981_Hydatigera_sp._(China)  ....A.....C..A........G..T..A.........................CT............T.A..A..C.....T..A..............
NC021141_H._parva                .......G.......G......G..TT.T......GT.........G...T..T..............T.A..............A............A.
NC021142_H._kepkogorski          .C.............GG........T..T..G..A.C.............T..GT.............T.............T..A..............

                                 cox2 10590          10600          10610          10620          10630          10640          10650          10660          10670          10680
                                 -------+---------+---------+---------+---------+---------+---------+---------+---------+---------+--
ON055368_H._taeniaeformis        TGTTCCGTATCATTTAGTTGTTACTTCAGCTGATGTTATTCATTCATTTTCTGTGCCATCTTTAAATTTAAAGATGGATGCTATACCTGGGCGTTTAAAT
FJ597547_H._taeniaeformis        C..C..................................................A................................A..A.........
JQ663994_H._kamiyai              ......A.......................A.......................T..........................A........A.....G...
NC037071_H._kamiyai              ......A.......................A.......................T..........................A........A.....G...
PP104554_H._kamiyai              ..................G...........A....................C..T..........................C........A.........
LC008533_Hydatigera_sp._(France) ..............................A...................................................A........A.....G...
NC061206_Hydatigera_sp._(China)  G..C..T.....A........A..A..T..A..............G.....G..T..T..A..........................G..T.........
MW808981_Hydatigera_sp._(China)  G..C..T.....A........A..A..T..A..............G.....G..T..T..A..........................G..T.........
NC021141_H._parva                G.....T.........A....A.....T...........C.....T........C..T..A.......................T.....T.........
NC021142_H._kepkogorski          ...A..T........G..G.....G..G..A........A.....T.....A..............C..G.....................A..G......
```

```
                                    cox2 10690     10700     10710     10720     10730     10740     10750     10760     10770     10780
                                    -------+---------+---------+---------+---------+---------+---------+---------+---------+---------+--
ON055368_H._taeniaeformis           CATATGTTTTTTTGTCCTTCTCAACATGGTTCTTTTATAGGTTATTGTGCTGAGTTATGTGGGGTGAATCATGGTGTTATGCCTATAGTTATAGAAGTAA
FJ597547_H._taeniaeformis           ............................................C.............................C.........................
JQ663994_H._kamiyai                 ..............................................................T...........A..G........T........G..G.
NC037071_H._kamiyai                 ..............................................................T...........A..G........T........G..G.
PP104554_H._kamiyai                 .......................G.............................AC.......T..A........G..G........T........G..T.
LC008533_Hydatigera_sp._(France)    .....................................................A........T...........A...........T.............
NC061206_Hydatigera_sp._(China)     ....................C..G........A........G..............G..C..T..T.................G..............T.
MW808981_Hydatigera_sp._(China)     ....................C..G........A........G..............G..C..T..T.................G..............T.
NC021141_H._parva                   ...T.............G.....G........A.................A.....G........T......A.GA..........T..A..T.....T.
NC021142_H._kepkogorski             ................................A....................A........C..A........AA.......A.....G.....G....

                                    cox2 10790 tRNA 10800 Glu 10810     10820     10830     10840     10850      10860     10870 nad6 10880
                                    -------+---------+---------+---------+---------+---------+---------+----- ----+---------+ ---------+
ON055368_H._taeniaeformis           TAGATACAATGGACAAGTTTTGTTTTAGTATAAGTATTGATTATTTTAACTTTTCGTGTTAAGGATAGTTTTT-AACTAAACAAAAGTA-GTGGTTAGCG
FJ597547_H._taeniaeformis           ..............T....................G.....................................-...............-........TA
JQ663994_H._kamiyai                 .......G.....T...................T....A..................................T..............G-..TA....T.
NC037071_H._kamiyai                 .......G.....T...................T....A..................................T..............G-..TA....T.
PP104554_H._kamiyai                 .............T...................T....A..................................TG.............G-..T.....T.
LC008533_Hydatigera_sp._(France)    .......T.....T...................T....A..................................TG.............G-A.T.....AT
NC061206_Hydatigera_sp._(China)     ........G.TA.T...................T.GG.T..................................TG..........G..G-A.TA.A..TA
MW808981_Hydatigera_sp._(China)     ........G.TA.T...................T.GG.T..................................TG..........G..G-A.TA.A..TA
NC021141_H._parva                   .T...GT..AAA.G...................T.---T.........G.........C.G.A.........A-...........TT..T..TA....AA
NC021142_H._kepkogorski             .............T.....................TG.A.......................A..........-G.............G-A.TA....AT

                                    nad6      10890     10900     10910     10920     10930     10940     10950     10960     10970
                                    -- -------+---------+---------+---------+---------+---------+---------+---------+---------+---------
ON055368_H._taeniaeformis           TC-TTATTAAGTTTATATTTATTTAATTTATTTCTGTTTTCTTTGATTAGAAATTGTATATATTATTGTATAATTTTGATTATTAAAGCTTTGTTGTCGA
FJ597547_H._taeniaeformis           .T-.........................................A.......G.............................................A.
JQ663994_H._kamiyai                 .T-.....G............C.A.....G...T..........AG...CT...........C..............A..............A..A..A.
NC037071_H._kamiyai                 .T-.....G............C.A.....G...T..........AG...CT...........C..............A..............A..A..A.
PP104554_H._kamiyai                 .T-..G........G.....G..A.....G...T.......A...G...CT.....C........C..........................A.....A.
LC008533_Hydatigera_sp._(France)    .T-.................G..A.....G...T..........AG...CT......G.G...........G....................AC....A.
NC061206_Hydatigera_sp._(China)     .T-..G..G.....T..............GA..T..........T...TCT........T...........TC.C..A...G..........A.....T.
MW808981_Hydatigera_sp._(China)     .T-..G..G.....T..............GA..T..........T...TCT........T...........TC.C..A...G..........A.....T.
NC021141_H._parva                   .TGC.G..TG..GCG.............C.A..A.A......A.T.G.....G.......ATA......T..T.A..AG..T.G......A.......TG
NC021142_H._kepkogorski             .T-...........G..CC.......A...A..T.A..C.....A..A..GG.......C...........G...C.T..C..A.........A.T..T.

                                   10980     10990     11000     11010     11020     11030     11040     11050     11060     11070 nad6
                                    +---------+---------+---------+---------+---------+---------+---------+---------+---------+---------
ON055368_H._taeniaeformis           GCTATATTGTGTATGAAGTAATGGGATTTAGTTGATATTCTTTAATTTTTTGTCTTATATATGTAGGTGGTGTTTATATATTATTTATCTTTGTGTCTTT
FJ597547_H._taeniaeformis           .........................................................................G..C...........T.....T.....
JQ663994_H._kamiyai                 .T.....A..A.T...G..G...........G................................G..C.....G..C..T........T...........
NC037071_H._kamiyai                 .T.....A..A.T...G..G...........G................................G..C.....G..C..T........T...........
PP104554_H._kamiyai                 .T..........T......G.....G.....A.........C.......C..............G..C.....G.....T........T.....A.....
LC008533_Hydatigera_sp._(France)    .T........A.T...G..G.....G.....G..G.............................T.....A..G...G.T........T.....A.....
NC061206_Hydatigera_sp._(China)     .T......A.A..C.....TT.A..G...........C...............T.A..T...A.T........G..CG.T........T...........
MW808981_Hydatigera_sp._(China)     .T......A.A..C.....TT.A..G...........C...............T.A..T...A.T........G..CG.T........T...........
NC021141_H._parva                   .G.TA..ATGC....T.A..TAT..G.AC.....G..CA....GT.A......T.G......A....G.....A.......CG.....T.....A..AG.
NC021142_H._kepkogorski             .T........A..C..G.............G......C.......C............T.....T.....G..G............G.T.....A.....
```

```
                                  11080     11090     11100      11110     11120     11130     11140     11150     11160     11170  nad6
                                  +---------+---------+-----  ----+---------+---------+---------+---------+---------+---------+--------
ON055368_H._taeniaeformis         TTTTAACCCTAATGATAGAGTTATGA-TTAGTTGAGGGTTTAATTTAGTGAGATTAGTGTTGATTTTTTTTGTTTTTGGTGTGTCTGTATATACGTTTTG
FJ597547_H._taeniaeformis         .....................C....-...........................................................A......T......
JQ663994_H._kamiyai               ......T......A....G...G.TG-...............G....A.AG.G.........GC..........G.......A.A...............
NC037071_H._kamiyai               ......T......A....G...G.TG-..........A....G....A.AG.G.........TC..........G.G.....A.A...............
PP104554_H._kamiyai               ......T..........A......T.-.A.....G..CA.A.GC...A.TG.G....C............C.C.G..T....A.A.........A.....
LC008533_Hydatigera_sp._(France)  ......T......A....G.....TG-.A.....G..A.........T....T.........G...........G.G.....A.AC..G...........
NC061206_Hydatigera_sp._(China)   .....G...A........T.G.G.AG-.A.T..AT..T..AG....G..T..G..G...GGTG.......GTG..GG.T...T...A.T.T..TT....A
MW808981_Hydatigera_sp._(China)   .....G...A........T.G.G.AG-.A.T..AT..T..AG....G..T..G..G...GGTG.......GTG..GG.T...T...A.T.T..TT....A
NC021141_H._parva                 .....GA......AG...TTA.G.TGC.A.A..A.AAA...G...GT...GTG..G.----AT.A..G.....GG..AT.T.AGG.T.GATGGGA.A..A
NC021142_H._kepkogorski           ......A......AAG........TG-...........A...GG..TA.A.C.A.G...GCAGC.......A..AGG.T...T....CG.T...A....T

                                   11180     11190     11200     11210     11220     11230     11240     11250     11260     11270  nad6
                                  -+---------+---------+---------+---------+---------+---------+---------+---------+---------+--------
ON055368_H._taeniaeformis         TGAGTTGATTGAATTTGAATTTAGAAGATGTGTGTGTAAATTTAAAGAGATTTGATTGTATGTATGTTTTGGTTTGATGTTGATGTTTGGGTTTGTTGTT
FJ597547_H._taeniaeformis         ..................................................................C..C..............................
JQ663994_H._kamiyai               .AG.........G..............T.........GG......GG..A....G..A.....T........A........A........A.........
NC037071_H._kamiyai               .AG.........G..............T.........GG......GG..A....G..A.....T........A........A........A.........
PP104554_H._kamiyai               .AGA..AG....G..............T.....A....GG....G............A.....T........G..A..................A.....
LC008533_Hydatigera_sp._(France)  .AGA...G....G.....G........T..........G.....G............A.....T........G........A.....C......A..A..
NC061206_Hydatigera_sp._(China)   .A.T..ATCA..G.....GA.G.....T.A.T......G.........AG...........A.T...............C.A............A.AA..
MW808981_Hydatigera_sp._(China)   .A.T..ATCA..G.....GA.G.....T.A.T......G.........AG...........A.T...............C.A............A.AA..
NC021141_H._parva                 .TCT..AG.AAGTG.G..G.....TGA..A.T.A....C..C..G........A.TA....A....C........T.CT..A........A..GT.G..G
NC021142_H._kepkogorski           .A.TA..T.GA.T.....G.....G..T...A.T....GG....G............A............A.G..A..T..A........T...A.A...

                                   11280 nad6 11290     11300     11310     11320     11330     11340     11350     11360     11370  tRNA Tyr
                                  -+---------+---------+---------+---------+---------+---------+---------+---------+---------+----   -
ON055368_H._taeniaeformis         TTAAGAATGTTGATGTCTTATAGAGTTGGGTTTTATCGTTAAAGTTCTAGCTTAACATATTTAATGTGGAGGGTTGTAAACTCTTTTAAGGTTTAT---A
FJ597547_H._taeniaeformis         .....................G....C................T....................................................---.
JQ663994_H._kamiyai               .....T...C.A..............A..A.............T......T...G.............A.........................GG---.
NC037071_H._kamiyai               .....T...C.A..............A..A.............T......T...G.............A.........................GG---.
PP104554_H._kamiyai               .....T...........A..C.....A..T.............T......T...G.............A..........................G---.
LC008533_Hydatigera_sp._(France)  ...........A..............A..A.............T......T.................A.........................GG---.
NC061206_Hydatigera_sp._(China)   ..G..TC.A..A.....A.G..AGAA...............G.T........................A.........................TG---.
MW808981_Hydatigera_sp._(China)   ..G..TC.A..A.....A.G..AGAA...............G.T........................A.........................TG---.
NC021141_H._parva                 ..GTC.T..G..T.TAGG.G..AGT.GAAA........A...--C...G.T.................A..A...................CC..GCCA.
NC021142_H._kepkogorski           ........AG..T..GGGA.....AG.AAT........A..G.T................G.......A.........................GG---.

                                     11380        11390     11400     11410 tRNA 11420 Leu 11430     11440     11450     11460      11470
                                  ----+-----   --+-----------+---------+---------+---------+---------+---------+---------+------ ---+--
ON055368_H._taeniaeformis         CCAGTTAGGAAT--ATATGTAAAGATGCCAGAATTAATGGGATTAATTTAGGATTAATTTATGATTTTTTATCTCTTTATTAATACGCATAAA-AACCCG
FJ597547_H._taeniaeformis         ..........T.--..........G.....................................................................-...T.-
JQ663994_H._kamiyai               ....C.......--T.T.......G..........G.................................-..........GT.A....TG...C......
NC037071_H._kamiyai               ....C.......--T.T.......G..........G.................................-..........GT.A....TG...C......
PP104554_H._kamiyai               ....C.......--T.G................C...................................-..........GT.A....TG...C......
LC008533_Hydatigera_sp._(France)  ....C.......--..........G.............................................-..........GT.A....TG...C......
NC061206_Hydatigera_sp._(China)   ....C......ACGT.G...............T..T.................................-.........C...A.G...G...-......
MW808981_Hydatigera_sp._(China)   ....C......ACGT.G...............T..T.................................-.........C...A.G...G...-......
NC021141_H._parva                 ....G..AATTAAAT.T.T.TG.ATAA..GC.G--G..CAAGCCG.A.A.AA...T.AGC..T...CGGCT.GAT.C.GCGGT..TT..A...-..TTTA
NC021142_H._kepkogorski           ..........TA--.G...................T.....G..........G................-...........G.A.....G...C......
```

```
                                           11480     11490          11500     11510     11520     11530     11540     11550     11560
                                        ---- ---+---------+--- --- --- +---------+---------+---------+---------+---------+---------+-------
ON055368_H._taeniaeformis               -AATA-ATTATTTATTTTTTATT-CGG-GTT-TTTATGCGTATATTTCATAAAAAAAAAATTTTTGACATATATATATACGTCAAAAAATTTTTTTTATG
FJ597547_H._taeniaeformis               ----------------------------------------------------------------------------------------------------
JQ663994_H._kamiyai                     -....-..C.AC.T...A.....-...-...G...CA....T..C-------------------------------------------------------
NC037071_H._kamiyai                     -....-..C.AC.T...A.....-...-...G...CA....T..CA...-........GT.................C..T.........AA......T..
PP104554_H._kamiyai                     -.G..-.CA.AAAT..GA...C.-...-...G...CA....T..CA.T.-.........T.....A.....G.......T..T......AA......T.A
LC008533_Hydatigera_sp._(France)        -..C.-....G..T.AA....G..-...-...G...CA....T..CA.T.-................A...............T..T......C.......T.A
NC061206_Hydatigera_sp._(China)         T....-...--..T...A.....A...-...T..--------------------------------------------.-----------------.CCCC
MW808981_Hydatigera_sp._(China)         T....-...--..T...A.....A...-...T..--------------------------------------------.-----------------.CCCC
NC021141_H._parva                       ATT.GT.AAGA.GCCAGAAA...AT..T...AA..TA.AA.TA.--.-------------------------------------.T.TG.CT...G.C.CT.T
NC021142_H._kepkogorski                 T.T..-.AC.AAA--AAA..TA.A...-...G...C.....T..C-.T.A.......C.A...............C..T..T.....TG.......T.A

                                        11570     11580       11590     11600     11610       11620      11630      11640 tRNA 11650 Ser 11660
                                        --+---------+-- --------+---------+---------+----- ----+---------+---------+---------+---------+-----
ON055368_H._taeniaeformis               AAATATACGCATAAA-AACCCGAATAAAAAATAAATAATTATTCGGGTT-TTTATGCGTATACTCATAAAAAATTTATGTTTGATTTGAAATCAAATTTAT
FJ597547_H._taeniaeformis               -------------------------------------------------------------------------------------.................
JQ663994_H._kamiyai                     ----------------------------------------------------------------.........-....CA.....................
NC037071_H._kamiyai                     .TG..A....TG...C..........T...A.GT.G...........G...CA....T............-....CA.....................
PP104554_H._kamiyai                     .TG..A....TG...C.......G...C...A.TT.G...........G...CA....T............-.....A.....................G.
LC008533_Hydatigera_sp._(France)        .TG..A....TG...C........C...TT.A..C....G........G...CA....T............-...........................
NC061206_Hydatigera_sp._(China)         TT---------------------------------------------------------------..........T-..G...................
MW808981_Hydatigera_sp._(China)         TT---------------------------------------------------------------..........T-..G...................
NC021141_H._parva                       .-------.----------------.T..--------------------------------.........C..----G.AC...............G...G.
NC021142_H._kepkogorski                 ...A.A.....G...C......T.....TTT.TTTGTT.A..A.......G...C.....T............-..A..A......................

                                          11670     11680      11690      11700      11710     11720      11730 tRNA 11740 Leu 11750      11760
                                        ----+---------+--------+- --------+------+---------+---------+---------+---------+---------+----
ON055368_H._taeniaeformis               TGTTTTTAATAACAATATGAGTTCCT-ATATTTATTTATGTGTAGTTATGTCAGAATTATATGAGTTAGTTTTAAGCATTAATTATGGAATTTCCTAACT
FJ597547_H._taeniaeformis               ..........................-.........................................................................
JQ663994_H._kamiyai                     .......................T..T..GA.A..G-TGA.........................................T.....G...
NC037071_H._kamiyai                     .......................T..T..GA....A-TGA.........................................T.....G...
PP104554_H._kamiyai                     .......................TTG---.A....--TA.........................................T.....G...
LC008533_Hydatigera_sp._(France)        ..........................-..GA.A..ACTA.........................................T.....G...
NC061206_Hydatigera_sp._(China)         .......GG.G.....G.....------.A.GGCG-.AA.....C..................................T.....GG..
MW808981_Hydatigera_sp._(China)         .......GG.G.....G.....------.A.GGCG-.AA.....C..................................T.....GG..
NC021141_H._parva                       ........T..........G...A-----.A.A..------....C..................................T......G..
NC021142_H._kepkogorski                 ...C....T.G............TTA---.A..T..GT.A-....C..................................T......G..

                                                  11770     11780 tRNA 11790 Arg 11800     11810      11820      11830 nad5 11840      11850
                                        ---            --+---------+---------+---------+---------+ ---------+-------+---------+---------+---
ON055368_H._taeniaeformis               ACT----------AACATATAGAAGACTTATGTTACGGCCATAAGAATGGTAATGT-TATACCGTATGTTTATTATGATGATTTTATTTTGTTTTAGTAT
FJ597547_H._taeniaeformis               ...--AATCT---...........................................-.....................AG...................G.
JQ663994_H._kamiyai                     ...TGAATGT---G..........C.......................A..T.A.-....T..........---...A...................G.
NC037071_H._kamiyai                     ...TGAATGT---G..........C.......................A..T.A.-....T..........---...A...................G.
PP104554_H._kamiyai                     ...AAAGTAT---........A.TA....................G.....T..-...........................A.....G.........G.
LC008533_Hydatigera_sp._(France)        ...AAAATAT---........A.G.C.......................TAT.-..........................A.....G..........G..G.
NC061206_Hydatigera_sp._(China)         ...GTGATGTTTGG......CA.T.....................G...GT.T.A.GC.T.A........C......AG.......CA....AG..--
MW808981_Hydatigera_sp._(China)         ...GTGATGTTTGG......CA.T.....................G...GT.T.A.GC.T.A........C......AG.......CA....AG..--
NC021141_H._parva                       .T.--T-------GG.....TTG.A...................TG.A...A..-.T..T......C....---.T.AG.A.......GCAGGGTC.T.
NC021142_H._kepkogorski                 ...--GGT-----........TTTA........................G.AT.--T.......................T.T.........ACA..A...T.
```

```
                                  nad5 11860     11870     11880      11890     11900     11910     11920     11930     11940     11950
                                  ------+---------+---------+---------  +---------+---------+---------+---------+---------+---------+--
ON055368_H._taeniaeformis         AATGCTTTTTGTTTTGGTAATGTTTGGGTTTTTATT-AAATTATAGTTATGTACTAAAACTTAGATTATTAAGGTTAAGAGGGTGAAAATGATTAGTCAA
FJ597547_H._taeniaeformis         ...........C.............A..........-................................................G..............
JQ663994_H._kamiyai               ...AT.....A..C.A.....T.....T..G.....-G......G.....A..A....GT...A...GC....A......A..........G.....T..
NC037071_H._kamiyai               ...AT.....A..C.A.....T.....T..G.....-G......G.....A..A....GT...A...GC....A......A..........G.....T..
PP104554_H._kamiyai               G...T.....AC...C.....T.....T..G.....-...C...G.....A..T....G....A....C..G.A...G.GA................T..
LC008533_Hydatigera_sp._(France)  GG.............A.....T.....T..G..G..-.............A..A....GT...A.C.G.....T......A..........G.....T..
NC061206_Hydatigera_sp._(China)   ----T.....A....A........GA.T..G.....-........A..T.A.TT...GGT.G.ATG........C..G.TA.T..G.........A.A..
MW808981_Hydatigera_sp._(China)   ----T.....A....A........GA.T..G.....-........A..T.A.TT...GGT.G.ATG........C..G.TA.T..G.........A.A..
NC021141_H._parva                 ...TT.A.G.A....A...T.T.G.TTTA.......T.....GGGT.G....G...G.GT..C-T..T...TCT.....GA.T.AT.G.......A.A..
NC021142_H._kepkogorski           .GCTT.A...T.AA.TA.GG.T..A.....A.....-...A.GGG....CA.TT...GTT...AT.....T..AC..G.TA.A........G.....T..

                                  nad5 11960     11970     11980     11990     12000     12010     12020     12030     12040     12050
                                  -------+---------+---------+---------+---------+---------+---------+---------+---------+---------+--
ON055368_H._taeniaeformis         ATTTATATTTGATAAGGTGACTATTTGCGTTTTAATGATGTTGATTATATGCTTCTTGTATGCTTATTTTTATACTAGTCATTATTTTTGTAATGATCGT
FJ597547_H._taeniaeformis         ...........................T...............C........................................................
JQ663994_H._kamiyai               ....G.G...........A......A................AG.A........T..A..C................A....................C.
NC037071_H._kamiyai               ....G.G...........A......A................AG.A........T..A..C................A....................C.
PP104554_H._kamiyai               ....G...........A.A......A.......G........A........T..T..A........C..........A....................CA
LC008533_Hydatigera_sp._(France)  ..................A........T..C...........A...........T..A.....A..................................C.
NC061206_Hydatigera_sp._(China)   G....AT...........AT.AT.G...A..............G..........T..A..C..G..C.....C...G...........G.......G.T.
MW808981_Hydatigera_sp._(China)   G....AT...........AT.AT.G...A..............G..........T..A..C..G..C.....C...G...........G.......G.T.
NC021141_H._parva                 T..C.AT.......TT..TT.A..G.G.......T.A......GG...C..T..T..T....T.AGC.................C....C...A...TTA
NC021142_H._kepkogorski           .....GTC..........A.G.T.AA.T..A..GG.T.....A..A.....T..T..T.....G..C.....C..A...........C...C....AGA.

                                  nad5 12060     12070     12080     12090     12100     12110     12120     12130     12140     12150
                                  -------+---------+---------+---------+---------+---------+---------+---------+---------+---------+--
ON055368_H._taeniaeformis         GCTGGTTGAGATTTGTTACAAATTATTGTTTTATTTGTTATTGTAATGGGTGTGTTAGTATGCACTGGTGATTTTTTAAGTACATTGATTTTTTGAGAAT
FJ597547_H._taeniaeformis         .......................................G.A...........A..G.....T....................TC...............
JQ663994_H._kamiyai               .T.A..........A..G.GT.....AA..........AGC...T.....AA.T..G..G..T................C...T..A........G....
NC037071_H._kamiyai               .T.A..........A..G.GG.....AA..........AGC...T.....AA.T..G..G..T................C...T...........G....
PP104554_H._kamiyai               ATAA....G.....A....GG.....A.....G.....A.....T.....AA.T..G..G..T............C...C...T..A........G....
LC008533_Hydatigera_sp._(France)  AT.A...T......A..G..G.....A.....G.....AG....T.....AA.T........T.....C..........C...T..A........G..G.
NC061206_Hydatigera_sp._(China)   .T......G.....A...A.GG....AACG..G.....AG....T......A.T.....G.CT.....G........G..A..T..A........G....
MW808981_Hydatigera_sp._(China)   .T......G.....A...A.GG....AACG..G.....AG....T......A.T.....G.CT.....G........G..A..T..A........G....
NC021141_H._parva                 TTGT.A.T......A.ATA.G.....A..A........GG........A.CT.......G.CTT.A.........G.G..A..G..A.............
NC021142_H._kepkogorski           ATCA...T..G.......A.G.....AAC.........GG....T......A.AC.GA...TT...AA.....A...G.C...T..A..A........G.

                                  nad5 12160     12170     12180     12190     12200     12210     12220     12230     12240     12250
                                  -------+---------+---------+---------+---------+---------+---------+---------+---------+---------+--
ON055368_H._taeniaeformis         ATCTTGGGGTTGTTAGATTTTTTTTGATTTTGTTTTATGATAAATATTTAAGTTTGCGTTCATCGGCTATTACTTTAGTTTCTTCTCGTTTTGGTGATGT
FJ597547_H._taeniaeformis         ...............................A..............................................................G.....
JQ663994_H._kamiyai               .......T..A..A...................................G..............A.T...A........C..............G..C..
NC037071_H._kamiyai               .......T..A..A...................................G..............A.T...A........C..............G..C..
PP104554_H._kamiyai               .......T..G.....G..............A..............C..G..............A..A.................G..............
LC008533_Hydatigera_sp._(France)  .......T..A......................................G..............A..C..........................G.....
NC061206_Hydatigera_sp._(China)   .C.....T..A..A..C........A........C...........C..G...C.A.....T..T.T............G....................
MW808981_Hydatigera_sp._(China)   .C.....T..A..A..C........A........C...........C..G...C.A.....T..T.T............G....................
NC021141_H._parva                 ..T.A.....G........C.....A.....A..C.CTCA.....T............A.....AAT.G.G..A.....A..A..A..G.....A.....
NC021142_H._kepkogorski           .......A.....G.................A..C....................A.....T..A.TA.....A.....C........G.....C.....
```

```
                                 nad5 12260     12270     12280     12290     12300     12310     12320     12330     12340     12350
                                 -------+---------+---------+---------+---------+---------+---------+---------+---------+---------+--
ON055368_H._taeniaeformis        TTGCTTGTTTTTGATAATTGGGTTAAGATGTTTTACTTTTAATAATATTATTCCTGCTTTAATTTATTTATTTTTTGTTGTGTTTTCTAAGAGTGCTGGT
FJ597547_H._taeniaeformis        ....C..............................................................C...........................GA..A
JQ663994_H._kamiyai              C..T..A.....AT.............C..............A........C...........C.....G.....GA.A..T..................
NC037071_H._kamiyai              C..T..A.....AT.............C..............A........C...........C.....G.....GA.A..T..................
PP104554_H._kamiyai              ...T..A.....AT.....A.A....AT..............A.G.G.............G..A..CC.G.....GA.A..T..............C...
LC008533_Hydatigera_sp._(France) ...TA.A.....AT.G.....A.....C..............A......G................C..G......A.A..T..............C...
NC061206_Hydatigera_sp._(China)  ...T.........T..........G..T....A..TGA....AGGAG...........G...CA..C..C..C..G.....A..................
MW808981_Hydatigera_sp._(China)  ...T.........T..........G..T....A..TGA....AGGAG...........G...CA..C..C..C..G.....A..................
NC021141_H._parva                G..T..A......T.....A.T....AT.A....TTA.A.......TGGT.A..A..AG.T..A.....T...C..A.AA.T..GA...........A..
NC021142_H._kepkogorski          ......A.....AT....A..T..G..T..C.AC.AC.A...A.G...GG.GTTG.GC...G.A.....C......A.AT....................

                                 nad5 12360     12370     12380     12390     12400     12410     12420     12430     12440     12450
                                 -------+---------+---------+---------+---------+---------+---------+---------+---------+---------+--
ON055368_H._taeniaeformis        TTTCCTTTTATAAGATGGTTGTTAGAAGCTATGCGTGCTCCTACCCCAGTTAGATCTTTAGTTCATTCTTCTACATTGGTAGCTGCTGGGATTTGATTTA
FJ597547_H._taeniaeformis        ....................................................................................................
JQ663994_H._kamiyai              .................A..........................T........G..A...........A........A..G........A..........
NC037071_H._kamiyai              .................A..........................T........G..A...........A........A..G........A..........
PP104554_H._kamiyai              ..............T..A..A.................G.....T...........A..G........A........A...........A..........
LC008533_Hydatigera_sp._(France) .................A..A..G....................T...........A...........A........A..G........T..........
NC061206_Hydatigera_sp._(China)  .................A....................C.....T..T.....G..A.....G........C..G........A.....A........C.
MW808981_Hydatigera_sp._(China)  .................A....................C.....T..T.....G..A.....G........C..G........A.....A........C.
NC021141_H._parva                .....A.....T..T......C....G..G.....A..G..A..T.....A.....G..G..............T..A........A..TG.A..G...G
NC021142_H._kepkogorski          ..............T.....A.......................A........T..A....................A...........A..........

                                 nad5 12460     12470      12480     12490          12500     12510     12520     12530     12540
                                 -------+---------+---- -----+---------+------     ---+---------+---------+---------+---------+------
ON055368_H._taeniaeformis        CTATGCGTTATGATTTATTCAA-TTTTATTAAAGATATAATTTGA-----TTTTCTAATCTGTTATTAATTAGCATATTTATAACTGGTGTAAGAAGAAT
FJ597547_H._taeniaeformis        ...................T..-......................-----.........T.............T..........................
JQ663994_H._kamiyai              T.........C........T..-............G...GC....-----..C.....AT.T..GC.T.....T......G.T........G........
NC037071_H._kamiyai              T.........C........T..-............G...GC....-----..C.....AT.T..GC.T.....T......G.T........G........
PP104554_H._kamiyai              T..................T..-................GC....-----........AT.C..GC.T.....A........T.................
LC008533_Hydatigera_sp._(France) T...............G..T.G-................GC....-----.........T.T..GC.T.....T........T.................
NC061206_Hydatigera_sp._(China)  T......C......C....T..A....T.C.T.AC.C..GCG..------......GGGT.....G.T.....A.....C..T.....C...........
MW808981_Hydatigera_sp._(China)  T......C......C....T..A....T.C.T.AC.C..GCG..------......GGGT.....G.T.....A.....C..T.....C...........
NC021141_H._parva                T.............A.T..GG.T.A..TA.C...T....GG..A.TCAGG......GTAG.....A.TT.A..A..TA.A..T......T.GT.TG.GT.
NC021142_H._kepkogorski          T..................T..-A...T...............AT-----.........T.A..G..G.....A...........C...A..........

                                  12550     12560     12570     12580     12590     12600     12610     12620     12630     12640 nad5
                                 ---+---------+---------+---------+---------+---------+---------+---------+---------+---------+------
ON055368_H._taeniaeformis        GTTTTTTTTAGACTTAAAGAAGATTGTGGCATTGTCTACTTGTAATAATATTTGTTGATGTGTATTATACTTAATTTGTGGGGGAGTTGTGTTATCGTTG
FJ597547_H._taeniaeformis        ............T.......................C................................T.......................G..A...
JQ663994_H._kamiyai              A...........T..G...........A..G..A..A...........A..............T........G........T..T...ACT........A
NC037071_H._kamiyai              A...........T..G...........A..G..A..A...........A..............T........G........T..T...ACT........A
PP104554_H._kamiyai              ......C.....T..G...........A.....A.................C...........T......C......A...T..G...A.T......A.A
LC008533_Hydatigera_sp._(France) ......C.....T..G..............G................................T.................T..G.....T..G..A..A
NC061206_Hydatigera_sp._(China)  ............T............A.A...C.T.....A.....A..A..............GC....T........A..TAA...CA.T..G..T..A
MW808981_Hydatigera_sp._(China)  ............T............A.A...C.T.....A.....A..A..............GC....T........A..TAA...CA.T..G..T..A
NC021141_H._parva                A...........T..G........AA.T..T..A.....A.....A..C............A..C.............T..TAA...AACA.....A..A
NC021142_H._kepkogorski          ............T..G.........A.A..T..A..G.....C........A.....G.....G.....T.......A...TAA....C.T.....A..A
```

```
                                         12650     12660     12670     12680     12690     12700     12710     12720     12730     12740 nad5
                                      ---+---------+---------+---------+---------+---------+---------+---------+---------+---------+------
ON055368_H._taeniaeformis             TTTCAACTTATTAGTCATGGAGTGTCTAAGTGTATATTGTTTATGTTAGTTGGTGATGTAATGAGAGGAAGAGGTGGATCTCAGGCTAGTAAATGTGTGT
FJ597547_H._taeniaeformis             .................................G...................G..............................................
JQ663994_H._kamiyai                   ....................G..A..............A..................A.......T.....T...............C............
NC037071_H._kamiyai                   ....................G..A..............A..................A.......T.....C...............C............
PP104554_H._kamiyai                   .....G..............G..A.........G.GC...................A.......T.....T..A.......A...................
LC008533_Hydatigera_sp._(France)      .....G..........C..G..A.........G...A....................A.......T.....C...........A...................
NC061206_Hydatigera_sp._(China)       ....................G..C.............C............................C..T..C..A..T..A..A.....A.....C....
MW808981_Hydatigera_sp._(China)       ....................G..C.............C............................C..T..C..A..T..A..A.....A.....C....
NC021141_H._parva                     ......T.GT.A........T..T...........C..A......C.TA.C.....A.......T..T..T.....T...........A..T.....A.
NC021142_H._kepkogorski               ..............A.....G..T...............A..............G..........T.....TA.C........A...........A.....

                                         12750     12760     12770     12780     12790     12800     12810     12820     12830     12840 nad5
                                      ---+---------+---------+---------+---------+---------+---------+---------+---------+---------+------
ON055368_H._taeniaeformis             ATAGAACTCGATTTTATGGTAATTGAAACTTGTTTAGATTATTAATAATAATTTTAGGTCTTTCAGGATTACCTTTTATAGGTGTGTTTTTTACTAAGCA
FJ597547_H._taeniaeformis             ..........................................G.........................................................
JQ663994_H._kamiyai                   .C...........................T.............A..........C..........T...............G...................
NC037071_H._kamiyai                   .C...........................T.............A..........C..........T...............G...................
PP104554_H._kamiyai                   ................C............T..A.......GC.G..............A.....T..GC..............................
LC008533_Hydatigera_sp._(France)      .............................T..A.......G..G..................T..G.................A...............
NC061206_Hydatigera_sp._(China)       ....T........................T..A....TG...G.G...G..........G.....T..T.................T............
MW808981_Hydatigera_sp._(China)       ....T........................T..A....TG...G.G...G..........G.....T..T.................T............
NC021141_H._parva                     ..GT.T..ACC..C.........A......AC.A......G....T..TGCGG..ACT..GT.AG.T..TG.T........T..G..A...............
NC021142_H._kepkogorski               .............................T..A.....G...G.TG..G...................G.....A.....C.....A...............

                                         12850     12860     12870     12880     12890     12900     12910     12920     12930     12940 nad5
                                      ---+---------+---------+---------+---------+---------+---------+---------+---------+---------+------
ON055368_H._taeniaeformis             TTATTTATTATCAGGACTTATTGGTGTTGTTAATGTTTTTGAATTGACGTTTATCTTGATTTGTATGTTTTTATCTTACTTTTATTCATTTCGTTTATGT
FJ597547_H._taeniaeformis             .............A...................................G.T..................................C........
JQ663994_H._kamiyai                   ............TA..T.A..G..G.....A...........G..A..A..AG.T..A...........C........T........C..C........
NC037071_H._kamiyai                   ............TA..T.A..G..G.....A...........G..A..A..AG.T..A...........C........T........C..C........
PP104554_H._kamiyai                   ...C........TA..T.A..G..G...A.............G..A.TA..AG.T..A.CA.......A..C.G.....T..........C.........
LC008533_Hydatigera_sp._(France)      ......G.....TA..T.G..G..G...A.C.......C..G..A.TAC.GG.T..A.C...................T.....C..........G...
NC061206_Hydatigera_sp._(China)       ......GC....TATGT.A.....G...AG...CA.AG..ATGACATGAA.AG.T..ATCA...........G.....T..A..C..G........G...
MW808981_Hydatigera_sp._(China)       ......GC....TATGT.A.....G...AG...CA.AG..ATGACATGAA.AG.T..ATCA...........G.....T..A..C..G........G...
NC021141_H._parva                     C.T...G..G..TATGA.G..A.....A.AA...A..A..AT.G.ATT.A.GT.T.CTT.G.......GG.....C..T..A..C..G.....A......
NC021142_H._kepkogorski               .......C....TATGT.AT.A..G...A.....A.A.G.AG...T.TAA.G..T..AG.A..............C..T..G..C..G............

                                         12950     12960     12970     12980     12990     13000     13010     13020     13030     13040 nad5
                                      ---+---------+---------+---------+---------+---------+---------+---------+---------+---------+------
ON055368_H._taeniaeformis             ACTGTAATAACTAATTTAAAGTCCAGTAGTACATGTGGGGTGTTATTTTTTTATGAATCTGGTTTGATAGTGTGTGGGTGGTTGTTCATAAAATTTTATA
FJ597547_H._taeniaeformis             ........T.............T...A.........T..A.......................C......A.............................
JQ663994_H._kamiyai                   GTG..G................T...A.....T...A...C........................A..T.CA....A..A..A..T..T........C.
NC037071_H._kamiyai                   GTG..G................T...A.....T...A...C........................A..TACA....A..A..A..T..T........C.
PP104554_H._kamiyai                   ..............C.......T...A.....T...A...C..................C..A..C..A........A..A..T..T.............
LC008533_Hydatigera_sp._(France)      ..A..GG...............T...AC....T...A..C..........C........C..A..T..A........AC.A..T..T.............
NC061206_Hydatigera_sp._(China)       .GA..G..T.G..........GTT.....CC.C.T...T..A..G..........A..........A..T..T.....T.........T..C.........
MW808981_Hydatigera_sp._(China)       .GA..G..T.G..........GTT.....CC.C.T...T..A..G..........A..........A..T..T.....T.........T..C.........
NC021141_H._parva                     G..A.....TT...A..T...AGT...TC..G..TC..A..A..G.......T.A....G..GGCT..T..C.....T..A..A..T............G
NC021142_H._kepkogorski               .TA...T..............GTT..C.A.T.T.TA..T..AC......CA..C.....G..G..A...ACCA.A..A..A..A..TG....T....T..
```

```
                                    13050     13060     13070     13080     13090     13100     13110     13120     13130     13140 nad5
                                    ---+---------+---------+---------+---------+---------+---------+---------+---------+---------+------
ON055368_H._taeniaeformis           TATTTTTTATTATAGATGAAAGCATAGGTGTTGGGTTTATATTAAGTGTGATGTTAATAGCATTTCAAGTGTTTTCTTGAGTAGTAAGTTTACTATTATA
FJ597547_H._taeniaeformis           ........C..G..............................CA..........A.....................G...........A....
JQ663994_H._kamiyai                 ......A...C..G........A..T..C........AG.........A......T.GTT.......T.AG.......G.CG..T.....TT..A.T..
NC037071_H._kamiyai                 ......A...C..G........A..T..C........AG.........A......T.GTT.......T.AG.......G.CG..T.....TT..A.T..
PP104554_H._kamiyai                 .............G........A..T........T.C.G.G.....AA.A..TC.GT.GTT.......T..G......TGA..........TT..A....
LC008533_Hydatigera_sp._(France)    .............G........AG.T............G........A.A......C.GTTG......T.AG..................T..A.T..
NC061206_Hydatigera_sp._(China)     ...C...C..GT.G.....GGTG.........AAT...G..G.T...T.AG..C....TTTG.CC...A.A..A....T.T.TC..G.C.GGG.C.....
MW808981_Hydatigera_sp._(China)     ...C...C..GT.G.....GGTG.........AAT...G..G.T...T.AG..C....TTTG.CC...A.A..A....T.T.TC..G.C.GGG.C.....
NC021141_H._parva                   ..G.G.C.G.GC.G.......ATG.GTA.T.G..TA.A..CAAT.....TGGT...G.TTT.......A.T..G..CGTTA.TA...T..ATT.GAGT..
NC021142_H._kepkogorski             C.........GT..........AG.T...G....AT..CT.GC.....A.A.AT...T..TT.G.G..GA.CA....A.T.A..T......G.T.GG.G..

                                    13150     13160     13170     13180     13190     13200     13210     13220     13230     13240 nad5
                                    ---+---------+---------+---------+---------+---------+---------+---------+---------+---------+------
ON055368_H._taeniaeformis           TGATAGGAAATTGTTTAGATCGTGAAGGAGAAGGTTGTTTGGTTGCGATAATTTAGTGGAATTTTGTTATAAAATTTTTTATACCATTGTTTTTTACTCT
FJ597547_H._taeniaeformis           ......A.....A.......A..........A.....C.....T..........................G.......C..............T...
JQ663994_H._kamiyai                 ......A...A.........TA.....A.....A.....C.....T...............G....C.....G.........CAT...A.A.....T...
NC037071_H._kamiyai                 ......A...A.........TA.....A.....A.....C.....T...............G....C.....G.........CAT...A.A....GT...
PP104554_H._kamiyai                 ...C..A.....A......G.TA.....A.....A..........T...............G.....................CA....ACA.C....T...
LC008533_Hydatigera_sp._(France)    ......A.....A......GT.......A.....A..A.......T.........G......A.G..........G.........CAT.....G.....T..C
NC061206_Hydatigera_sp._(China)     ......A..C..A......AAA..G..A.....A..A.....G..T......A.....A..G.GC.T....GCTG...C....CGT..AA.AGC...T.T.
MW808981_Hydatigera_sp._(China)     ......A..C..A......AAA..G..A.....A..A.....G..T......A.....A..G.GC.T....GCTG...C....CGT..AA.AGC...T.T.
NC021141_H._parva                   ...G..A..TA.T..C..TAAT.....A..T..T..A........T...............GCA.......CG.GA.......AGTGA..AAAC..TGT.
NC021142_H._kepkogorski             .........T.A.T......AAA.....A.........A.....G.........C.....T...A.G.T.....GTGGG......TAT..CA.GAG...T.T.

                                    13250     13260     13270     13280     13290     13300     13310     13320     13330     13340 nad5
                                    ---+---------+---------+---------+---------+---------+---------+---------+---------+---------+------
ON055368_H._taeniaeformis           AGTTTATTGTTTTTTTCGTTGAGATAATTTAATGATGTGTTTATTTGGTAATATGGGCCGAAAAAGGATTGTGAATAGGTTTAAATGAATTTTATTAAACA
FJ597547_H._taeniaeformis           ...C.G..........................................................G....A............................T.
JQ663994_H._kamiyai                 ..AC.G..A..........G....G...G.....A........C..A..A..A...........A.....A....AA..........C...G..G..TG
NC037071_H._kamiyai                 ..AC.G..A..........G........G.....A........C..A..A..A...........A.....A....AA..........C...G..G..T.
PP104554_H._kamiyai                 ..C..G..A..........G........G..................A.....A..A...A.......A.....A....AA.............GC.T..T.
LC008533_Hydatigera_sp._(France)    ..G.....A..........G........G...G.A..................A..A...........A.....A....AA........GGC.........T.
NC061206_Hydatigera_sp._(China)     ...C.............A...............G...A.C......T....A..A...........A..AA.T...G.AC...G.....GG...TC.TG.AG
MW808981_Hydatigera_sp._(China)     ...C.............A...............G...A.C......T....A..A...........A..AA.T...G.AC...G.....GG...TC.TG.AG
NC021141_H._parva                   ..AC.T..T........A.........AAA.G.TT.TAA...T..GACAGGAT.A..G.T.........GAAA..A.TT......T...T.A....A....AT
NC021142_H._kepkogorski             ..G.....A.CAC....A...............G.A.....G...TA...AT.A..........AAG..T.A.G..TA.......A..G.....C.....TG

                                    13350     13360     13370     13380     13390 nad5 13400     13410     13420     13430      13440
                                    ---+---------+---------+---------+---------+---------+---------+---------+---------+------ ---+-----
ON055368_H._taeniaeformis           TATTGGTTATCGGAGTTTTTAGCTTTATATTTTATTTGGTGCTTATTTAATTTGATATGGTAAAATTTTAGGTTCGTCTTGCGTTGTAAA-TTTTGTTCG
FJ597547_H._taeniaeformis           ..........T..G...........................................................................CA....G.......A
JQ663994_H._kamiyai                 ..C.A..AGCTA.TA.......T..............AA.AA....C.....------------------.....TA.T....A.C.C...A........A
NC037071_H._kamiyai                 ..C.A..AGCTA.TA.......T.....G........AA.AA....C.....------------------.....TA.T....A.C.C...A........A
PP104554_H._kamiyai                 ....A..GG.T..GA.....G.T......A.A.....A.CTA....A..G..------------------.....T..TG...AC.TA...G.......TA
LC008533_Hydatigera_sp._(France)    ....A..A..TA.TA.A.....T................A.CA....A.....------------------.....TA.T....A.C.CG..G........A
NC061206_Hydatigera_sp._(China)     .GA.AA....A...A.....G.A..A..C.......C.A...G.G..G..G...-..T.AA.TTT..A......ATCG.C....GC.AC...G.......T.
MW808981_Hydatigera_sp._(China)     .GA.AA....A...A.....G.A..A..C.......C.A...G.G..G..G...-..T.AA.TTT..A......ATCG.C....GC.AC...G.......T.
NC021141_H._parva                   .C..A..AT.TA..A....CG.TA.C.CGC.....G..A.AT..T.G...CACA.------.--------.AA.AATGT..ATA.AT..T.G.AACAAATA
NC021142_H._kepkogorski             .GC.T...T.ATC.AGAG.G..T......TC...T...AA.AA.A..G..GA.CA.---AT.--T---.CGC...AAATCA....GTAC..G.......T.
```

```
                                    13450     13460     13470     13480                  13490      13500      13510     13520     13530
                                  ----+---------+---------+---------+-------             --+-------- -+-- -------+---------+---------+
ON055368_H._taeniaeformis         TCAATAAATAATATTTATTTATTATAATAACAAATATAAATA-------------TAAAAAATAGA-TGTA-ATATATACAACATATATAAGATAAGATA
FJ597547_H._taeniaeformis         ..........................................-------------...........-..C.-............................
JQ663994_H._kamiyai               .T..A..G..................................AATATAAAATATG..TG...CGA.G.AC.T........................T...
NC037071_H._kamiyai               .TG.A..G..................................AATATAAAATATG..TG...CGA.G.AC.T........................T...
PP104554_H._kamiyai               .T..A.....................................------TAAAATA..TG...CG..G.TGTA........................T...
LC008533_Hydatigera_sp._(France)  .T..A.....................................--TATAAAAAATA..TGG..CG..G...-.........................T...
NC061206_Hydatigera_sp._(China)   ...............
MW808981_Hydatigera_sp._(China)   ...............
NC021141_H._parva                 .-....T.C..ACA.G..AC..A......TAGT......CC.-------------..T.--------CCCTAGGG....T..T---.....T....T-..
NC021142_H._kepkogorski           .TG.A.....................................-----------------------------A...A.C.TGGA.CG-.AGT.....T...

                                        13540     13550      13560     13570     13580      13590      13600     13610
                                  ---- -----+---------+---- -----+---------+---------+-------- -+-----     ----+---------+
ON055368_H._taeniaeformis         TATA-GGGGGGGTATATACCCCCCC-TATATATCTTATCTTATATATGTTGTATATATAA-TATATAG-----ATATATCTCTCTATA------------
FJ597547_H._taeniaeformis         ....-.....T-------.A.....-..................................-.......-----...............------------
JQ663994_H._kamiyai               ....--..................TA.......--........................T-.......TATAG......A........TGTTATGTAGTA
NC037071_H._kamiyai               ....--.......G..........TA.......--........................T-.......TATAG......A........TGTTATGTAGTA
PP104554_H._kamiyai               ....TA.......G..........TA.......A.........................T-.......-----......A........TGTTATGTATAT
LC008533_Hydatigera_sp._(France)  ....--..................TA.......--........................T-.......---AT......A........TGTTATGTA-TA
NC061206_Hydatigera_sp._(China)
MW808981_Hydatigera_sp._(China)
NC021141_H._parva                 ....CCCTA........GGTAA.AT-....AG.---.CTA...GCTAACCA..GCC...TGC......T----.CT...AATGT....TGTTCA
NC021142_H._kepkogorski           ....TA.......GA.........TA.......---..AA.......A.A.-.......TC.......-----......A...A....GAT---------

                                                     13620     13630     13640     13650     13660     13670     13680      13690
                                     --          -------+---------+---------+---------+---------+---------+---------+------ ---+-----
ON055368_H._taeniaeformis         ---TA-----------ATAGAGAGATATATCTATATATTAAATAAACTATAAGTCATCAATTATTTATTATAGTTAATAGTTATTTAATT-TATATATTA
FJ597547_H._taeniaeformis         ---.C-----------...................................................C......................-.G......G
JQ663994_H._kamiyai               TCA..CTACATAACAT.......T...........C--..T..........................G.TAGA....C............-...G.....
NC037071_H._kamiyai               TCA..CTACATAACAT.......T...........C--..T..........................G.TAGA....C............-...G.....
PP104554_H._kamiyai               ACA..--A-----CAT.......T.............CA.T..........................G.TA......C............-...G.....
LC008533_Hydatigera_sp._(France)  ACA..----------T.G...T.T.............G..T.C........................A.TG.T.A.GC............-...G.....
NC061206_Hydatigera_sp._(China)
MW808981_Hydatigera_sp._(China)
NC021141_H._parva
NC021142_H._kepkogorski           ATA..-TC-----TAT.....T.T....C.A.........T........C.................G.TGTA.A.G.TA.AT..A....AC.....C..

                                    13700     13710      13720     13730     13740     13750      13760         13770     13780
                                  ----+---------+-------- -+---------+---------+---------+-------- -+---------    +---------+----- ---
ON055368_H._taeniaeformis         GTACAGTTGTGAAGACTTAGTGG-TTGTTTGATGACTTATAGTTTATTTAATTTGATATGGTAA-AATTTTAGGTT----CGTCTTGCGTTGTAAA-TTT
FJ597547_H._taeniaeformis         A..G...................-....C...................................-...........----..........CA....G...
JQ663994_H._kamiyai               T...CCC............TA..G..T.G..................A...........TA.TTT..G......CGTTGA...T..A.A....G.GA...
NC037071_H._kamiyai               T...CCC............TA..G..T.G..................A...........TA.TTT..G......CGTTGA...T..A.A....G.GA...
PP104554_H._kamiyai               ...TCC......G.....GT...-....G..................A...........A..T--.GG...----------..TG...AC.TA...G...
LC008533_Hydatigera_sp._(France)  T...CC.CA..........TA..G..A.G................G.A......-.......TTT..G....-------...CGA...A.C.CG..G...
NC061206_Hydatigera_sp._(China)
MW808981_Hydatigera_sp._(China)
NC021141_H._parva
NC021142_H._kepkogorski           ...TC----...T.....GA...-....C.........G........A....AG.....ATGT.C.CGC.G.--..T-AA..CGAAAT....ATT.A...
```

```
                                    13790     13800      13810
                                     -+---------+--------- +----
ON055368_H._taeniaeformis            TGTTCGTCAATAAATAATATT-TATTT
FJ597547_H._taeniaeformis            .....A...............G.G..CT
JQ663994_H._kamiyai                  ....TA.T..A.TGGG-..A.A.T
NC037071_H._kamiyai                  ....TA.T..A.TGGG-..A.A.T
PP104554_H._kamiyai                  ....AAATG.A.TTG--..G.
LC008533_Hydatigera_sp._(France)     .....A.T..A..GGGG..A.A..
NC061206_Hydatigera_sp._(China)
MW808981_Hydatigera_sp._(China)
NC021141_H._parva
NC021142_H._kepkogorski              .A..GCATG..GTT.--.GA.G
```

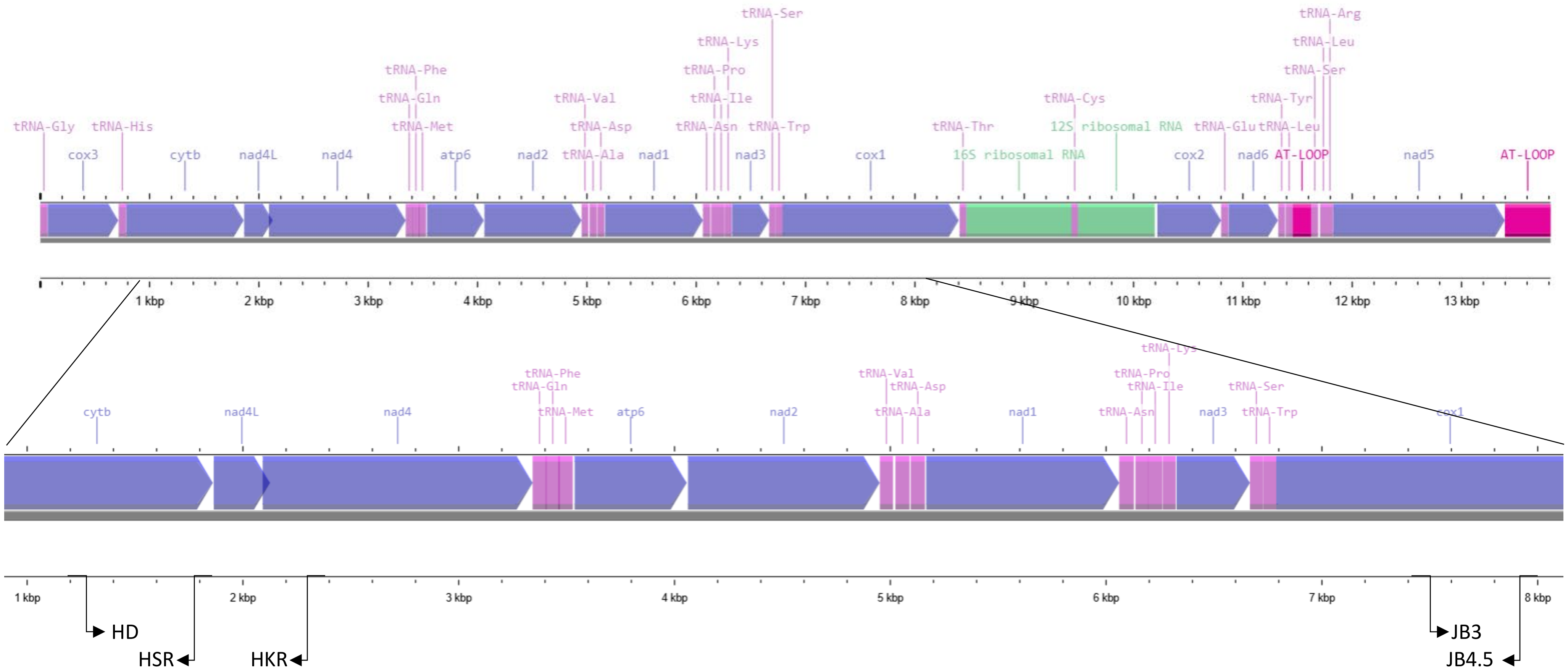

Supplement: Supplementary file 1 [file animals-15-03340-s001.zip › animals-3937711-supplementary.pdf]
